# High Levels of Sample-to-Sample Variation Confound Data Analysis for Non-Invasive Prenatal Screening of Fetal Microdeletions

**DOI:** 10.1371/journal.pone.0153182

**Published:** 2016-06-01

**Authors:** Tianjiao Chu, Suveyda Yeniterzi, Svetlana A. Yatsenko, Mary Dunkel, Patricia A. Shaw, Kimberly D. Bunce, David G. Peters

**Affiliations:** 1 Department of Obstetrics, Gynecology and Reproductive Sciences, University of Pittsburgh, Pittsburgh, PA, United States of America; 2 Center for Fetal Medicine, Magee-Womens Research Institute, Pittsburgh, PA, United States of America; Warwick University, UNITED KINGDOM

## Abstract

Our goal was to test the hypothesis that inter-individual genomic copy number variation in control samples is a confounding factor in the non-invasive prenatal detection of fetal microdeletions via the sequence-based analysis of maternal plasma DNA. The database of genomic variants (DGV) was used to determine the “Genomic Variants Frequency” (GVF) for each 50kb region in the human genome. Whole genome sequencing of fifteen karyotypically normal maternal plasma and six CVS DNA controls samples was performed. The coefficient of variation of relative read counts (cv.RTC) for these samples was determined for each 50kb region. Maternal plasma from two pregnancies affected with a chromosome 5p microdeletion was also sequenced, and analyzed using the GCREM algorithm. We found strong correlation between high variance in read counts and GVF amongst controls. Consequently we were unable to confirm the presence of the microdeletion via sequencing of maternal plasma samples obtained from two sequential affected pregnancies. Caution should be exercised when performing NIPT for microdeletions. It is vital to develop our understanding of the factors that impact the sensitivity and specificity of these approaches. In particular, benign copy number variation amongst controls is a major confounder, and their effects should be corrected bioinformatically.

## Introduction

Definitive prenatal diagnosis of genetic disease is currently performed via amniocentesis (AF) or chorionic villus sampling (CVS). These are invasive procedures that carry an inherent risk of miscarriage, fetal morbidity and parental anxiety[1–7]. Because of these risks, there has been considerable interest in the development of non-invasive tests that can be performed on a maternal blood sample in early gestation. One promising approach involves the analysis of cell-free fetal DNA in maternal plasma, which can serve as a substrate for fetal genotyping[8]. These nucleic acids are thought to arise from apoptotic cells known as syncytiotrophoblastic microparticles that are shed from the placental villus into the uterine circulation[9–12]. Progress in this field has rapidly accelerated following the demonstration that whole genome sequencing of maternal plasma DNA can be used for the non-invasive prenatal testing (NIPT) of common aneuploidy, including successful translation of these methods into the clinical setting[13–15].

Other chromosomal abnormalities such as microdeletions and microduplications occur with approximately twice the frequency of aneuploidy[16, 17] and are, therefore, of major clinical significance. Because of this, a number of methods for the diagnosis of microdeletions and microdeletions have been developed. However, the majority of these anomalies are smaller in size than the limits of detection of metaphase chromosome analysis. Thus, array comparative genomic hybridization (aCGH) has emerged as a powerful tool for their high-resolution detection. Unfortuately, however, aCGH must be performed utilizing AF or CVS.

In 2011 we demonstrated proof of concept that NIPT can be used for the non-invasive detection of fetal microdeletions[18] and this has since been confirmed by at least three other groups[19–21]. NIPT for microdeletions and microduplications is technically challenging, with complications arising from the potential for false positive and false negative results, unanticipated findings, and relatively high cost due to the amount of sequence data required. Furthermore, our understanding of the utility of this approach in a broad sense is limited. In light of this we tested the hypothesis that inter-individual variation in genomic copy number of control DNA samples is a confounding factor in the non-invasive prenatal detection of fetal microdeletions via the sequence-based analysis of maternal plasma DNA. We furthermore describe the analysis of a 318.46kb interstitial chromosome 5p microdeletion and highlight a number of key observations relating to NIPT in this context.

## Materials and Methods

### Human tissue samples

**Fig 1:**
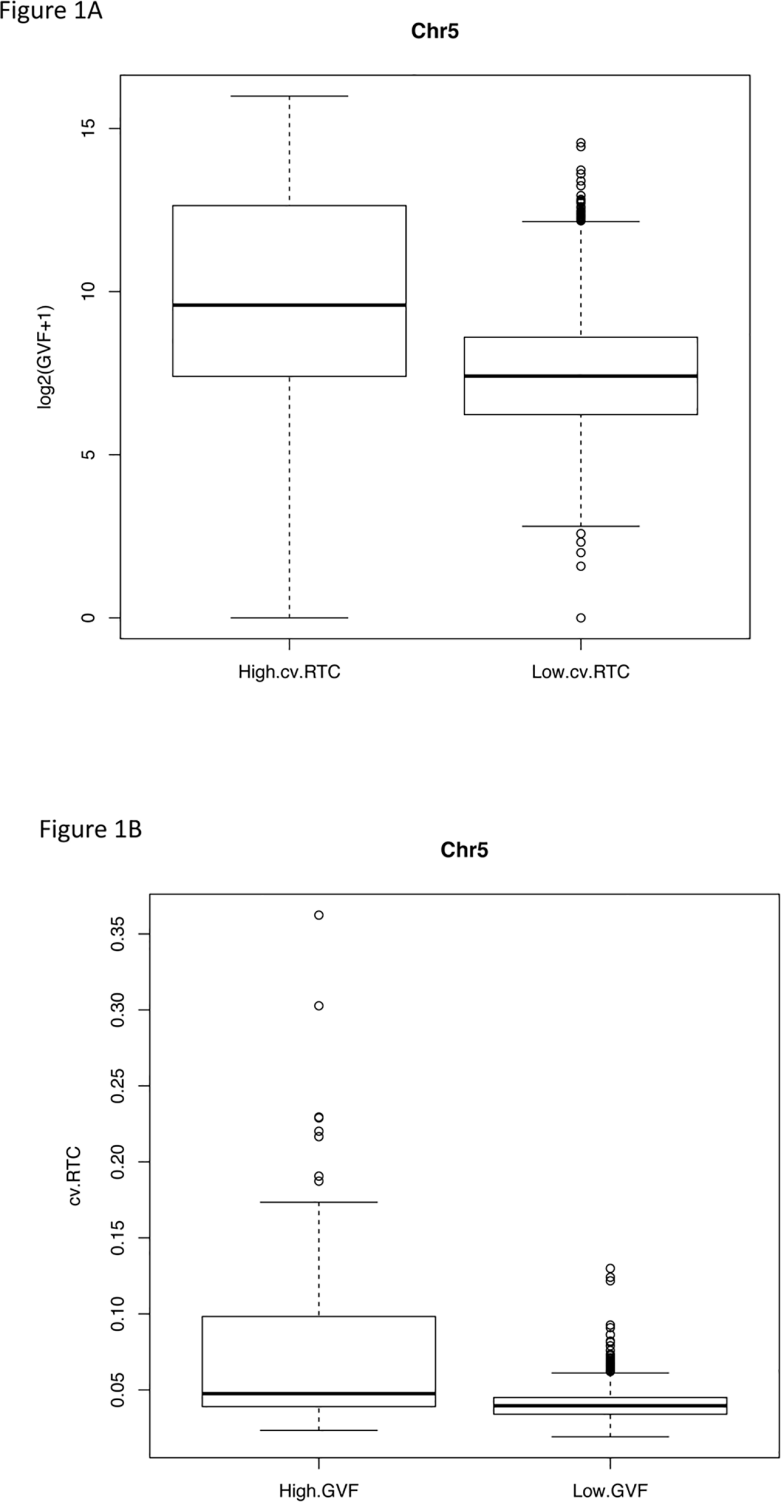
Comparative Genomic Hybridization Analysis of Affected Fetal Sample (First Pregnancy). Array CGH profile showing an interstitial deletion in the short arm of chromosome 5. Top: Ideogram of chromosome 5. The deleted 5p15.33 region is indicated by a red rectangle. Below: A magnified view of the 5p subtelomeric region. Positions are displayed according to GRCh37/hg19 Genome Browser. Shaded blue area indicates a loss in DNA copy number detected by 22 oligonucleotide probes (blue dots), located in the interval chr5:174,979–493,441 and encompassing an approximately 318 kb segment.

All patients provided written informed consent for use of their samples and clinical data. These women received prenatal care at Magee-Womens Hospital within the years 2008–2013, and were recruited from clinics and private practices. This protocol was approved by the University of Pittsburgh Institutional Review Board (Protocol # IRB PRO07090033). A family presented to Magee-Womens Hospital of UPMC at the Medical Genetics Department. The mother was Gravida eight, Para three, and was seen at 19.0 weeks gestational age due to an increased risk for Down Syndrome (1:42) and open neural tube defect (1:160) by multiple marker screen. An amniocentesis was performed, and during ultrasound for the procedure, bilateral club feet and an echogenic bowel were noted. Karyotype and FISH analysis from the amniocentesis revealed 46,XX. Comparative genomic hybridization (CGH)-based microarray analysis revealed a 318.46kb loss of 5p15.33 (Fig 1).

### DNA extraction and sequencing

Plasma handling, DNA extraction and DNA sequencing were performed as previously described[18].

### Data analysis

Data were analyzed using the previously described GCREM approach[22]. Briefly, GCREM is a linear mixed effect model for the read counts of different genomic regions in a DNA library, where the GC content is an independent variable with a library specific random coefficient. This linear mixed effect model is fitted using a set of libraries with known normal genomes to estimate the region specific corrections for a list of genomic regions of interest. Then we apply the corrections to the target library, and fit a linear model using only the regions that are believed to be normal in the target library. We then predict the read count for the regions of interest. By comparing the observed read counts with the predicted read counts, we can test if the suspected regions indeed carry insertions or deletions.

## Results

Summary statistics for sequencing libraries described herein are shown in S1 Table.

### High variance in sequencing read counts amongst normal samples strongly correlates with the frequency of genomic copy number variants

We hypothesized that the locus-specific variation of sequence read counts in libraries from clinically normal samples caused by benign copy number variation will negatively affect the performance of sequencing based copy number alteration (CNA) detection algorithms. Common to all sequencing based CNA detection algorithms, the test for the presence of a CNA is dependent on three parameters: T: a measurement of the normalized read count of the region of interest in the target sample; C: the average of the normalized read count in that region over a group of normal control samples; S: the standard deviation of the normalized read count for that group of normal control samples. The test statistic usually can be expressed as (T–C) ⁄ S. With benign copy number variants in the group of normal control samples, we could see a biased C and an inflated S, both of which will reduce the sensitivity and specificity of the CNA detection algorithm.

**Fig 2:**
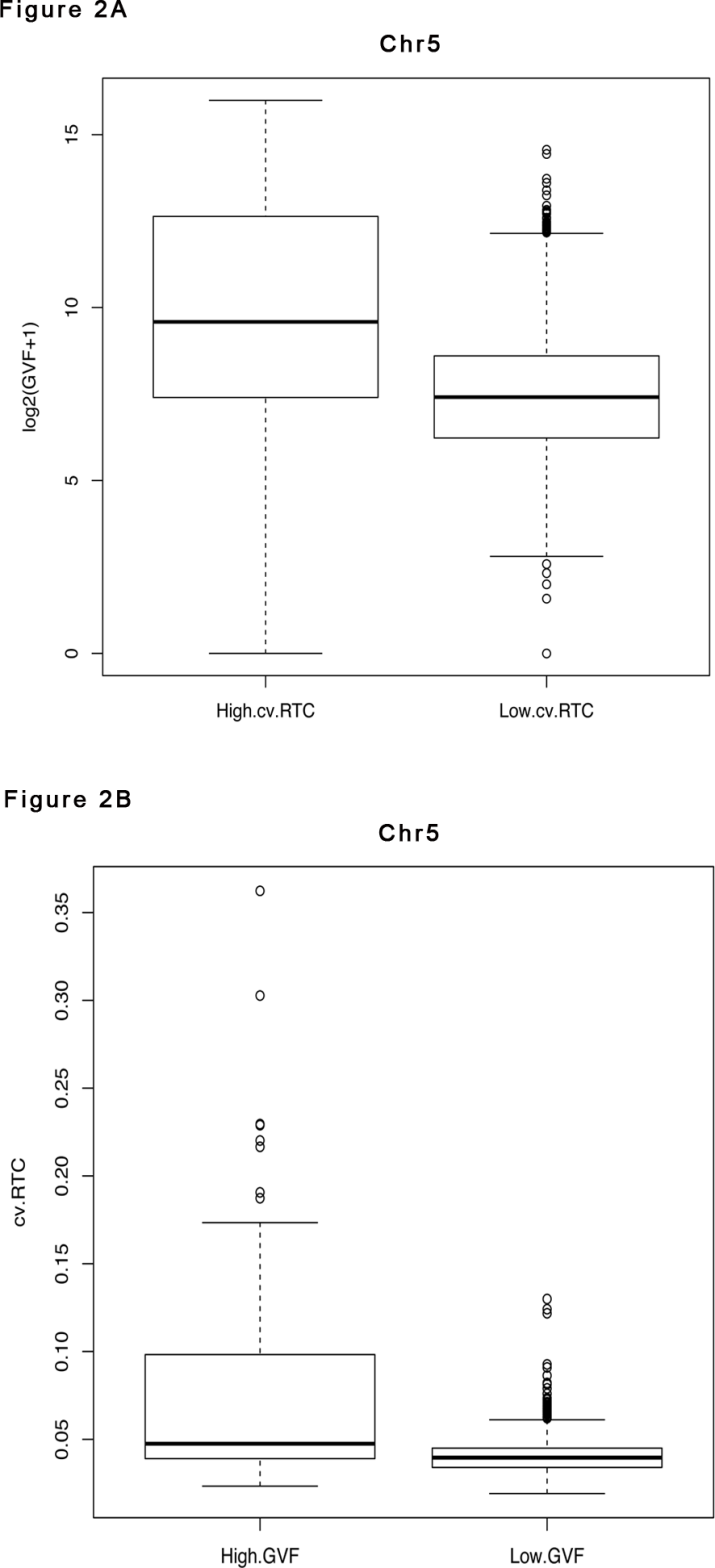
Boxplots of Genomic Variants Frequency (GVF) and coefficient of variation of Relative Read Counts (cv.RTC). (A) Boxplots of log2(GVF+1) for regions with high cv.RTC (above 95% quantile of the cv.RTC of all regions on chromosome 5) and low cv.RTC (below 95% quantile of cv.RTC) in maternal plasma control libraries on chromosome 5 (representative). (B) Boxplot of cv.RTC of the maternal plasma control libraries for regions with high GVF (above 95% quantile of the GVF of all regions on chromosome 5) and low GVF (below 95% quantile of GVF) on chromosome 5 (representative).

**Fig 3:**
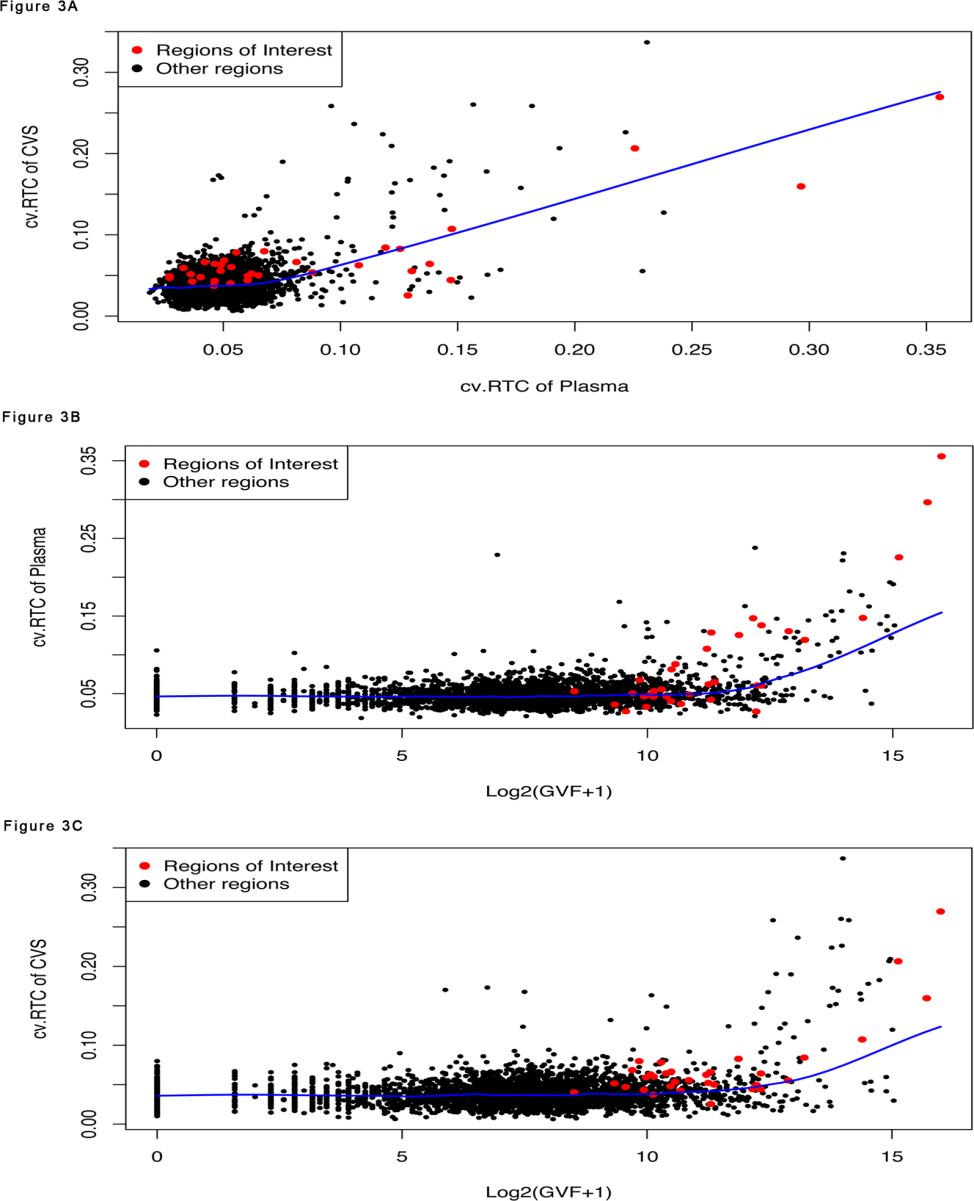
Plots of Coefficient of Variation of the Relative Read Count (cv.RTC) for Chromosome 5. (A) The cv.RTC of the 50kb regions in the CVS control libraries (y axis) against that in the maternal plasma control libraries (x axis) for chromosome 5. (B) The cv.RTC of the 50kb regions in the plasma control libraries (y axis) against the log2(GVF+1) of the 50kb regions reported in the DGV database (x axis) for chromosome 5. (C) The cv.RTC of the 50kb regions in the CVS control libraries (y axis) against the log2(GVF+1) of the 50kb regions reported in the DGV database (x axis) for chromosome 5. In all three plots, red dots represent 50kb regions around the microdeletion (see Fig 3), black dots represent other 50kb regions in chromosome 5.

To examine the relation between the variation of sequence read counts in libraries from clinically normal samples and benign copy number variation, we selected fifteen maternal plasma libraries from normal pregnancy samples that had been corrected for GC bias (S1 Fig). We divided the human reference genome (GRCh37) into 50kb non-overlapping regions, and calculated the relative read count (RTC) for each 50kb region, which is defined as the ratio of the read count in a given 50kb region to the median of the read counts in all 50kb regions in the whole genome (excluding regions with no aligned reads in all libraries). We also downloaded the Database for Genomic Variants (DGV)[23]. We first counted, for each 5kb region in human genome, the number of structural variants reported in the DGV database overlapping with that 5kb region (NDGV). Then we calculated, for each 50kb region, the Genomic Variants Frequency (GVF) as the sum of NDGV over the ten 5kb regions inside the 50kb region. Recall that GVF is the number of genomic variants observed in each 50kb region. It is log2 transformed so that its distribution is closer to a Gaussian distribution. We found a strong dependency between the coefficient of variation of the RTC (cv.RTC) across the fifteen libraries for each 50kb region, and the risk of structural variation, measured as log2(GVF+1), for that 50kb region. For example, in each autosome, regions with high cv.RTC (> = 95% quantile of the cv.RTC of all 50kb regions in that chromosome) have GVF values that are significantly higher than regions with low cv.RTC (< 95% quantile) (Wilcoxon two sample test) (Figs 2A and 3B, S2 Fig and S2A Table). On the other hand, the cv.RTC in regions with high GVF [log2(GVF+1) > = 95% quantile of log2(GVF+1) of all 50kb regions in each chromosome] is significantly higher than in regions with low GVF [log2(GVF+1) < 95% quantile] (Figs 2B and 3B, S2 Fig and S3A Table).

The high sample-to-sample variation observed in the fifteen plasma samples in certain regions is not coincidental. We further analyzed the six unrelated control CVS obtained between 10 and 13 weeks gestation, and found high sample-to-sample variation in the same regions. More precisely, after correction for GC bias, for each of the 22 autosomes, we found that the cv.RTC of the 50kb regions in the CVS samples is significantly and positively correlated with that in the plasma samples (t test, p values < 2.2e-16) (Fig 3A, S2 Fig), and shows a strong dependency with the risk of structural variation (log2(GVF+1)) in the corresponding regions (Fig 3C, S2 Fig, S2B and S3B Tables). We also explored other data preprocessing options, including deriving RTC from the read count data without GC content correction, as well as using different parameters for the alignment, e.g., allowing mismatch vs not allowing mismatch, and found similar results (Table 1). Interestingly, the CVS samples tend to have smaller coefficients of variation than the plasma samples. Allowing mismatch and correcting for GC content can also reduce the coefficient of variation, especially for the plasma samples (Table 1).

**Table 1:** The 25%, 50%, 75%, and 97.5% quantiles of the coefficient of variation of RTC in chromosome 5. For CVS, the coefficient of variations were estimated from 6 libraries. For maternal plasma, the coefficient of variations were estimated from fifteen libraries unrelated to the CVS samples.

Sample	GC correction	Allow mismatch	25.00%	50.00%	75.00%	97.50%
CVS	Yes	No	0.0286	0.0370	0.0471	0.0784
CVS	Yes	Yes	0.0231	0.0297	0.0369	0.0632
CVS	No	No	0.0293	0.0381	0.0487	0.0871
CVS	No	Yes	0.0239	0.0307	0.0386	0.0738
Plasma	Yes	No	0.0383	0.0464	0.0563	0.1131
Plasma	Yes	Yes	0.0324	0.0391	0.0475	0.1027
Plasma	No	No	0.0463	0.0554	0.0676	0.1214
Plasma	No	Yes	0.0402	0.0481	0.0593	0.1151

Note that the samples included in the DGV database are not necessarily representative of the population from which we collected the plasma and CVS control samples. This makes the strong correlation between the cv.RTC of the plasma and CVS control libraries on the one side, and the GVF derived from the DGV database on the other side even more remarkable.

### The effect of high variance of read counts on the ability to detect a fetal microdeletion by sequencing maternal plasma DNA

The effect of high variance of the read counts on the non-invasive prenatal diagnosis of fetal microdeletion is best illustrated by the following case study. A plasma DNA sample (PL741) from a mother carrying a fetus with a confirmed paternally inherited ~318.46Kb interstitial deletion on chromosome 5 between 174,979–493,441 (hg19) (Fig 1) was obtained at 32.4 weeks gestation and subjected to paired end (100 x 2) whole genome sequencing on the Illumina HiSeq 2000.

**Fig 4:**
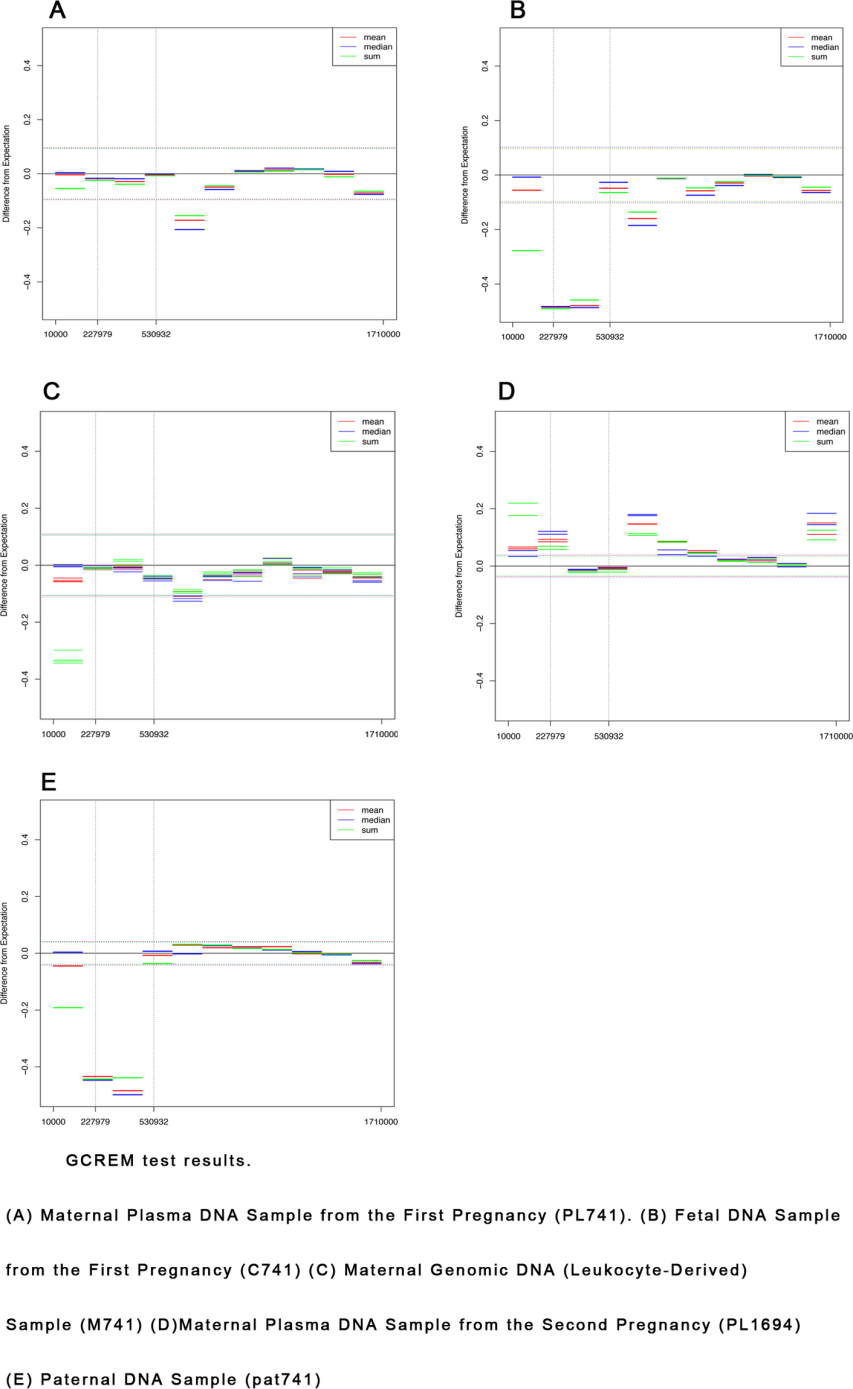
GCREM Tests of Chromosome 5 Microdeletion. (A) GCREM test of the maternal plasma library (data is from 1 of a total of 13 HiSeq 2000 lanes) from the first pregnancy. (B) GCREM test of the fetal amniocyte library from the first pregnancy. (C) GCREM test of the maternal genomic libraries (data is from 4 HiSeq 2000 lanes) from the first pregnancy. (D) GCREM test of the maternal plasma library from the second pregnancy (PL1694). (E) GCREM test of the paternal genomic (leukocyte-derived) library (pat741). For all plots, Y axis is the difference between observed RTC and expected RTC. Positive values mean potential gain, negative values mean potential loss. X axis is the position on the chromosome. Each horizontal bar represents a 150kb region. Dotted lines are standard deviation of the expected RTC.

We first tested for the presence of the microdeletion in PL741 using the fifteen confirmed karyotypically normal control plasma samples discussed above. As shown in Fig 4A, there was no clear evidence that the PL741 microdeletion was detectable using this approach.

We also sequenced DNA from fetal amniocytes (C741), maternal leukocytes (M741), and a paternal sample (Pat741) obtained from the affected PL741 pregnancy. We used the six karyotypically normal CVS tissue samples discussed above as controls, so that the pure maternal, paternal, and fetal samples from pregnancy PL741 could be compared with “like”, high molecular weight nuclear DNA, controls rather than plasma-derived DNA. As shown in Fig 4B, we were clearly able to detect copy number loss in the confirmed 318.46Kb deleted region in the pure fetal (C741). As expected, consistent with paternal transmission of this mutation, we found in the paternal sample (Pat741) an almost identical pattern of copy number loss (Fig 4E) to the pure fetal sample, but no evidence of copy number loss in the maternal leukocyte sample (M741) (Fig 4C).

In order to confirm that fetal DNA in the PL741 plasma was indeed at a detectable level and within the expected range of sensitivity of our assay we analyzed the sequencing data from the PL741 plasma sample to determine the percentage of fetal genome equivalents. We used a method that we developed for this purpose, which is a modification of our previously published work[24]. Briefly, we identified SNPs that are homozygous in the maternal sample and heterozygous in the fetal sample. The frequency of the paternally inherited allele to the maternal allele then is half of the fetal frequency. Using generalized linear model, our estimation of the fetal DNA frequency in the first plasma data is approximately 15%, which is well within the expected normal range for such samples.

Eventually, we were able to obtain a chorionic villus sample from a subsequent pregnancy involving the same family. Again, microarray analysis was performed (not shown) and the same chromosome 5 microdeletion was identified. A plasma sample (PL1694) was obtained at 11 weeks. GCREM analysis of the maternal plasma (PL1694) from the second pregnancy showed the same results as those (PL741) from the first pregnancy in that the 318.46Kb deletion was not detected (Fig 4D).

A close examination of the fifteen plasma and six CVS control samples showed that, despite corrected for GC bias (S1 Fig), the read counts of the regions of interest in these controls displayed extremely high sample-to-sample variation. We found that, across the control samples, the coefficient of variation of the RTC in the regions of interest are much higher than other regions of chromosome 5. For example, for the RTC derived from the GC corrected read count data, the lower quartile, median, upper quartile, and 97.5% of the coefficient of variation for all chromosome 5 regions are 0.038, 0.046, 0.056, and 0.113 respectively. However, among the 31 regions of interest (located in chr5:10000–1710000), 17 of them have a coefficient of variation greater than the upper quartile, and 11 of them have a coefficient of variation greater than the 97.5% quantile.

## Discussion

We present an analysis of sequenced normal maternal plasma and CVS DNA samples that were used as controls in the context of NIPT for fetal microdeletions and microduplications. We found that in some genomic regions, these control samples exhibited extremely high variance, as observed in both the CVS and plasma control data. The high variance in these regions is not incidental, because the distribution of high variance regions across the whole genome is strikingly similar between the fifteen maternal plasma libraries and the six unrelated CVS libraries. More importantly, we found a strong correlation between the distribution of the high variance regions in both the plasma and CVS libraries and the distribution of reported structural variation in the DGV database. Statistical tests showed that the RTCs of the regions where the DGV database reported a high degree of structural variation have significantly higher coefficient of variation than the regions with fewer reported structural variations.

The presence of regions with high risk of structural variations in the control libraries represents a serious challenge to the application of NIPT technologies for the detection of microdeletions and microduplications. While the number of high-risk regions may be small, the chance that a structural variation will occur in such regions is high. Using the data from the DGV database, about 7% of the 50kb regions were found to have a GVF of 2000 or higher, but these account for 70% of total GVF reported in the database.

To illustrate, we used those fifteen plasma samples as controls to investigate a maternal plasma sample with microdeletion on the short arm of chromosome 5. Because of the extremely high variance in the control samples over this region, we were unable to detect this via non-invasive methods, despite the comprehensive analysis of plasma DNA using proven statistical tools.

Based on the results presented above, in order to apply NIPT technology to the detection of a broad spectrum of microdeletions and microduplications, the influence of structural variations in the control plasma samples would need to be mitigated. To do this, we would ideally need to obtain a subset of plasma samples with no structural variants, at least over the regions of interest, so that these samples could serve as the controls for the microdeletion and microduplication tests. However, given the diversity of the human genome, it is difficult, if not impossible, to find samples containing sequence over a large region that is identical to the reference genome. Instead, we recommend correcting the effect of structural variants on a given maternal plasma-sequencing library such that a corrected library will have a read count distribution over the regions of interest similar to that of a sample of the reference genome.

Briefly, this would require matched maternal plasma (MP), maternal blood cells (MBC), and CVS samples. By deep sequencing of the maternal and fetal genomes, preferably enriched over the regions of interest, we can assemble the maternal and fetal genomes against the reference genome. Importantly, because the mother and the fetus share a copy of chromosome sets, we can derive the haplotype information for both the mother and the fetus over the regions of interest. By sequencing the maternal plasma samples, we can also estimate the percentage of fetal DNA in maternal plasma[24]. Then we can determine either analytically or by simulation how the structural variants in the maternal and fetal genomes affect, in maternal plasma sequencing libraries, the distribution of reads over the reference genome. In turn we can correct the maternal plasma libraries to remove these effects so that the read distribution in the corrected libraries would be similar to that from libraries generated from the reference genome. The availability of control plasma samples with corrected read counts over the regions of interest will greatly increase the range of application of the NIPT technology from regions with low risk of copy number variation to regions of higher risk of copy number variation, so that it can be offered as a viable and risk-free alternative to invasive diagnostic procedures.

## Supporting Information

S1 Fig GC Correction Curves for Fifteen Karyotypically Normal Control Plasma Samples and 6 Karyotypically Normal CVS Samples. The curves represent a nonparametric estimation of the relationship between the read count in a genomic region and the GC content of that genomic region. The X axis is the GC content of 50kb genomic regions. The Y axis is the number of reads aligned to a 50kb region.
(PDF)

S2 Fig Correlation Between Read Count Variance and Frequency of Structural Variation for All Chromosomes. There are 22 groups of plots in this file for the 22 autosomes. Each group has three plots. In each group, the first plot is the cv.RTC of the 50kb regions in the CVS control libraries against that in the maternal plasma control libraries for each autosome. The second plot is the cv.RTC of the 50kb regions in the plasma control libraries against the log2(GVF+1) of the 50kb regions reported in the DGV database. The third plot is the cv.RTC of the CVS control libraries against the DGV database.
(PDF)

S1 Table Summary statistics for Sequencing Libraries. (XLS)

S2 Table **(A).**Comparison of the number of structural variants reported in the DGV database between regions in fifteen maternal plasma control libraries displaying high and low cv.RTC **(B).** Comparison of the number of structural variants reported in the DGV database between regions in six CVS controls libraries displaying high and low cv.RTC.
(DOCX)

S3 Table (A).Comparison of the coefficient of variation of relative read count between regions in fifteen plasma controls libraries displaying high and low numbers of reported structural variants in the DGV database. **(B).** Comparison of the coefficient of variation of relative read count between regions in six CVS controls libraries displaying high and low numbers of reported structural variants in the DGV database.
(DOCX)
