# Correction: High Levels of Sample-to-Sample Variation Confound Data Analysis for Non-Invasive Prenatal Screening of Fetal Microdeletions

**DOI:** 10.1371/journal.pone.0163578

**Published:** 2016-09-20

**Authors:** Tianjiao Chu, Suveyda Yeniterzi, Svetlana A. Yatsenko, Mary Dunkel, Patricia A. Shaw, Kimberly D. Bunce, David G. Peters

Fig 1 is an incorrect duplication of Fig 2. The authors have provided a corrected version here.

**Fig 1:**
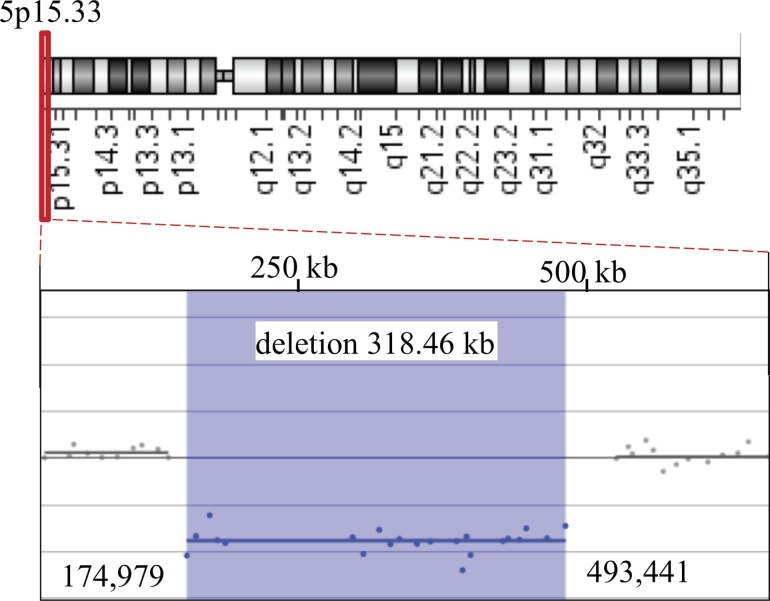
Comparative Genomic Hybridization Analysis of Affected Fetal Sample (First Pregnancy). Array CGH profile showing an interstitial deletion in the short arm of chromosome 5. Top: Ideogram of chromosome 5. The deleted 5p15.33 region is indicated by a red rectangle. Below: A magnified view of the 5p subtelomeric region. Positions are displayed according to GRCh37/hg19 Genome Browser. Shaded blue area indicates a loss in DNA copy number detected by 22 oligonucleotide probes (blue dots), located in the interval chr5:174,979–493,441 and encompassing an approximately 318 kb segment.
