# Supplementary material for: High Levels of Sample-to-Sample Variation Confound Data Analysis for Non-Invasive Prenatal Screening of Fetal Microdeletions
Source: PLoS One. 2016 Jun 1;11(6):e0153182. doi: 10.1371/journal.pone.0153182 (PMC4889033; doi:10.1371/journal.pone.0153182)
Supplement: S1 Fig — The curves represent a nonparametric estimation of the relationship between the read count in a genomic region and the GC content of that genomic region. The X axis is the GC content of 50kb genomic regions. The Y axis is the number of reads aligned to a 50kb region. (PDF) [file pone.0153182.s001.pdf]

Figure S1.

Plasma control samples are designated with the prefix “PL”.
Chorionic villus control samples are designated with the prefix “CVS”

PL1438

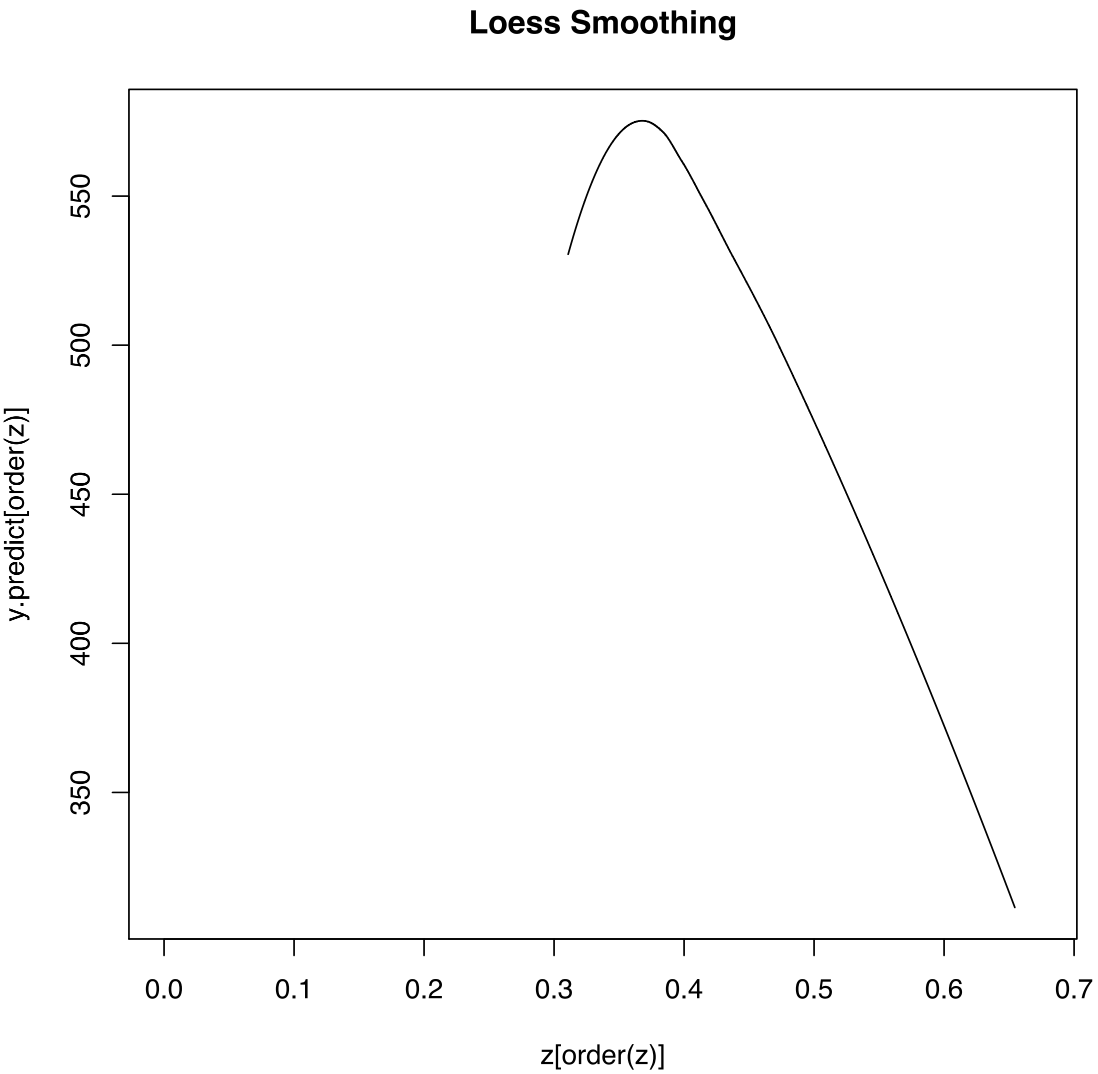

PL1421

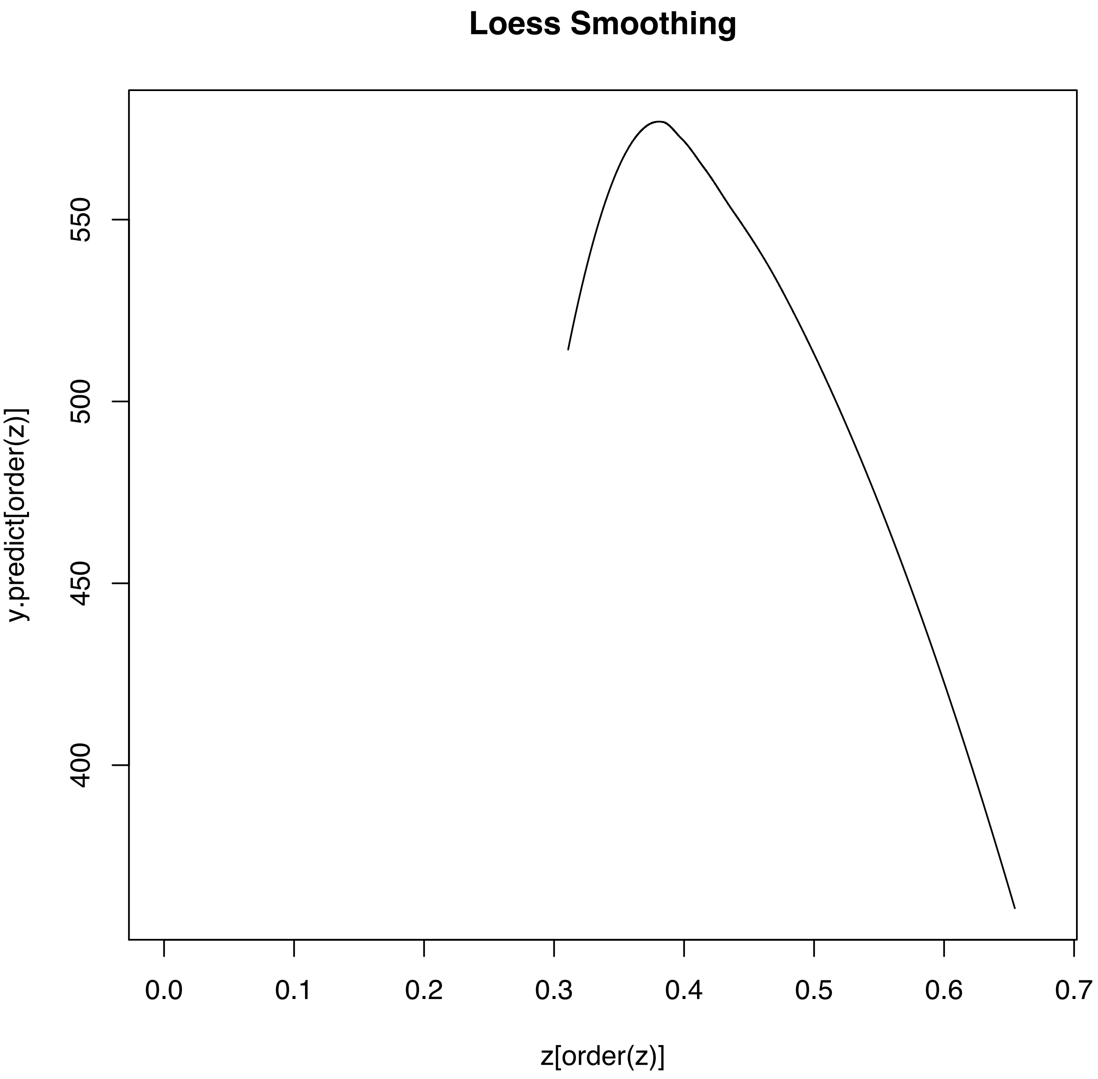
Loess Smoothing
y.predict[order(z)]
400
450
500
550
0.0
0.1
0.2
0.3
0.4
0.5
0.6
0.7
z[order(z)]

PL1411

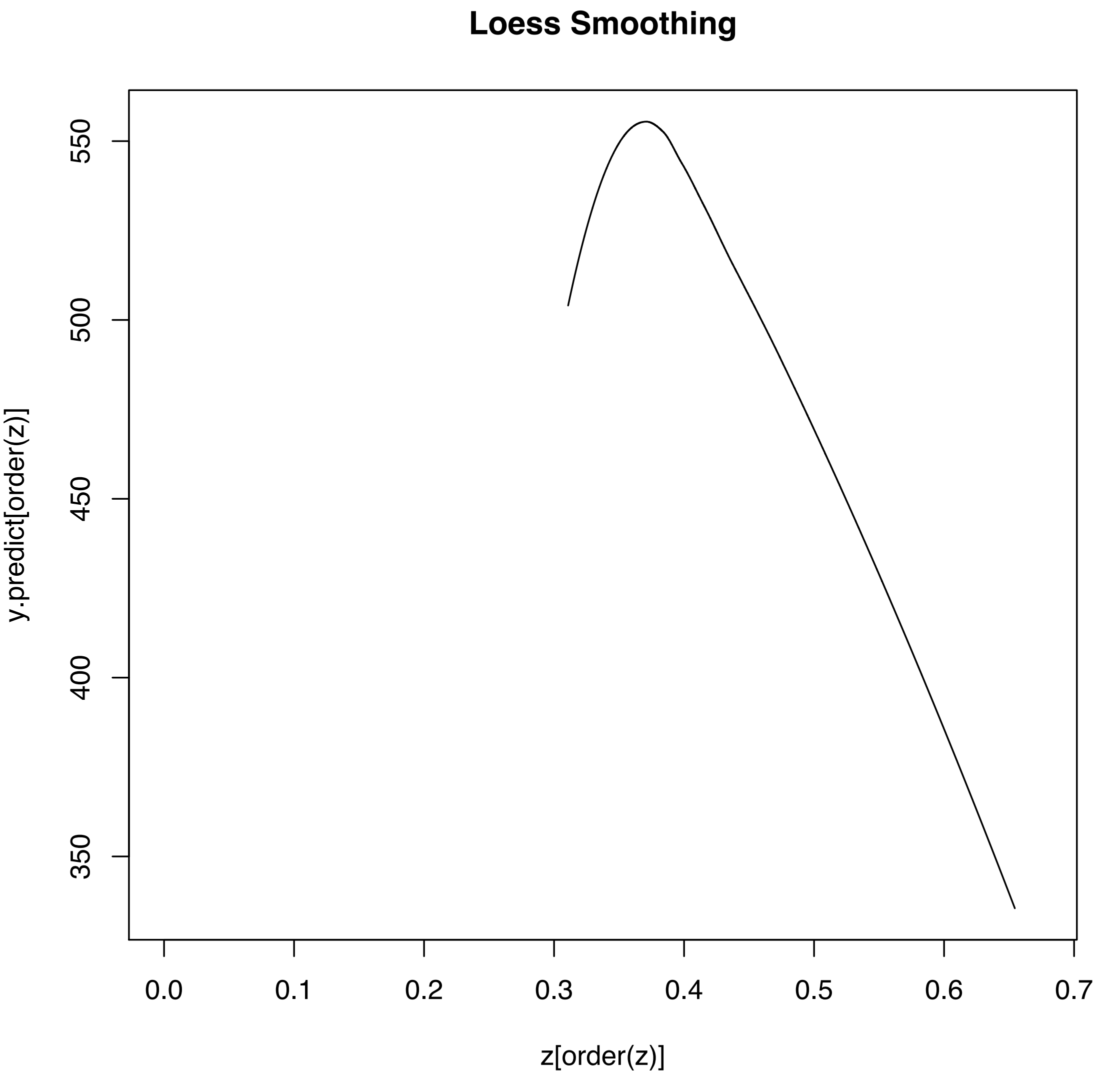
Loess Smoothing
y.predict[order(z)]
350
400
450
500
550
0.0
0.1
0.2
0.3
0.4
0.5
0.6
0.7
z[order(z)]

PL1405

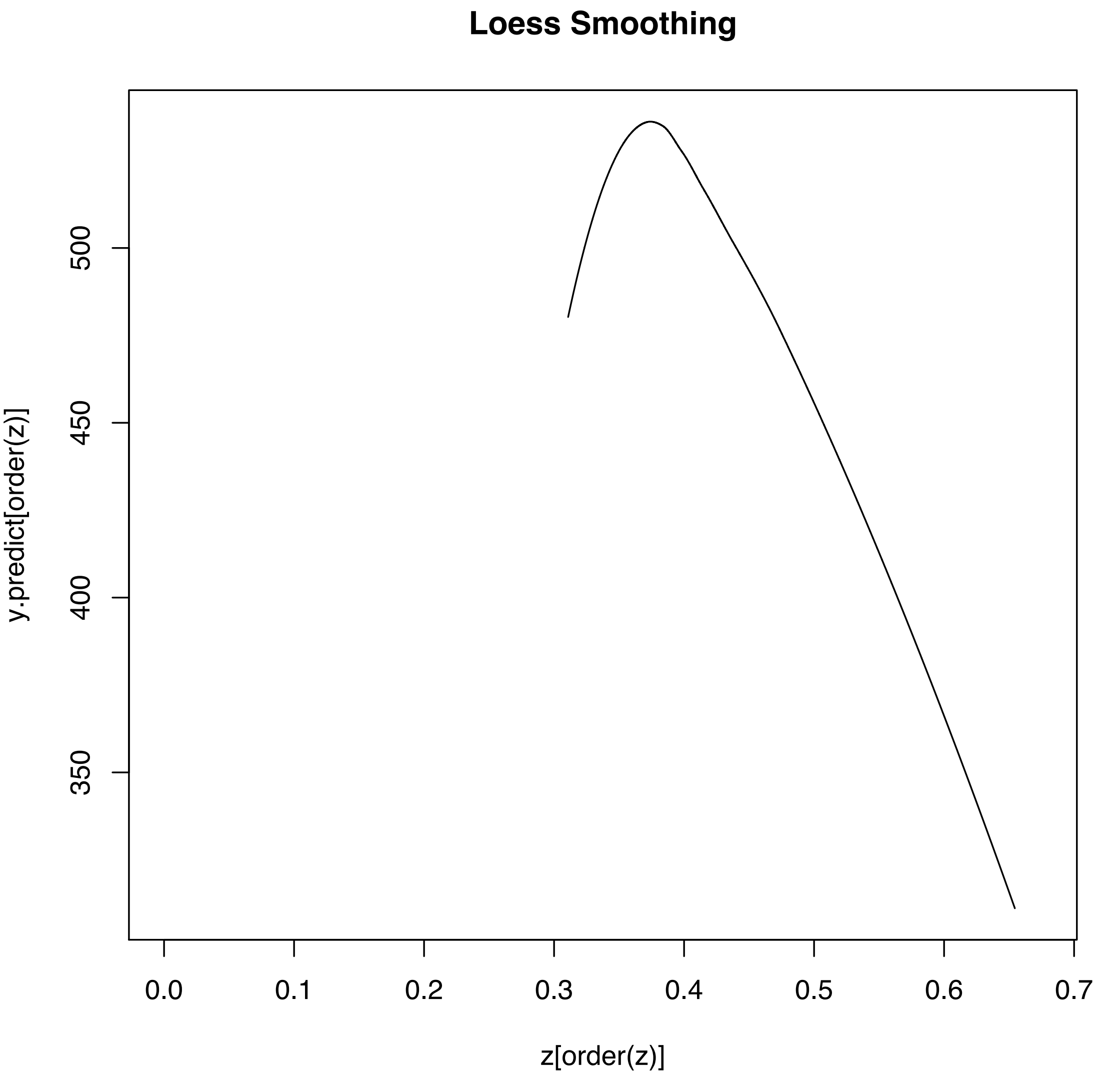

Loess Smoothing
y.predict[order(z)]
500
450
400
350
0.0
0.1
0.2
0.3
0.4
0.5
0.6
0.7
z[order(z)]

PL1390

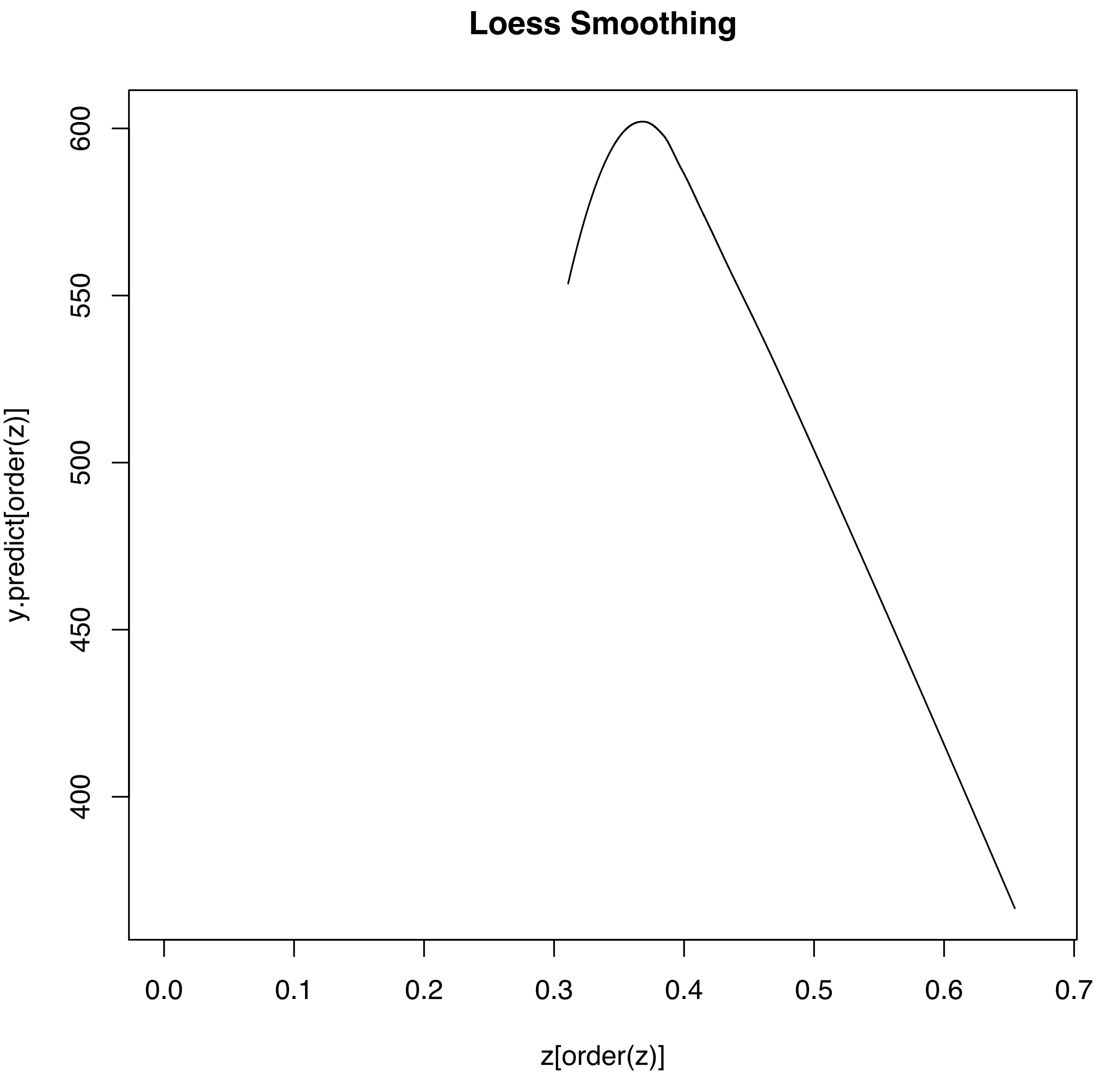
Loess Smoothing
600
550
500
450
400
y.predict[order(z)]
0.0
0.1
0.2
0.3
0.4
0.5
0.6
0.7
z[order(z)]

PL1388

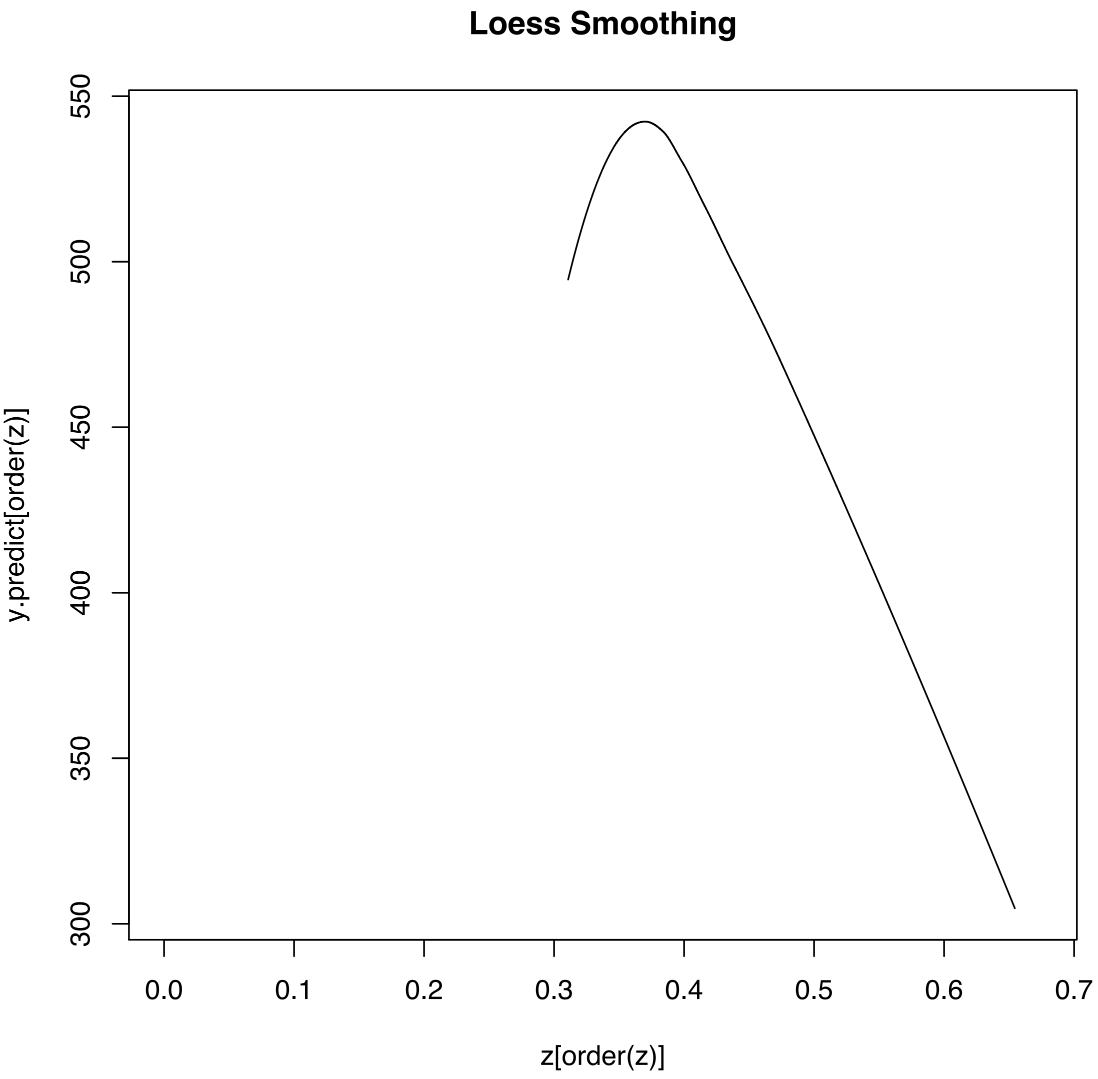

Loess Smoothing
y.predict[order(z)]
550
500
450
400
350
300
0.0
0.1
0.2
0.3
0.4
0.5
0.6
0.7
z[order(z)]

PL1351

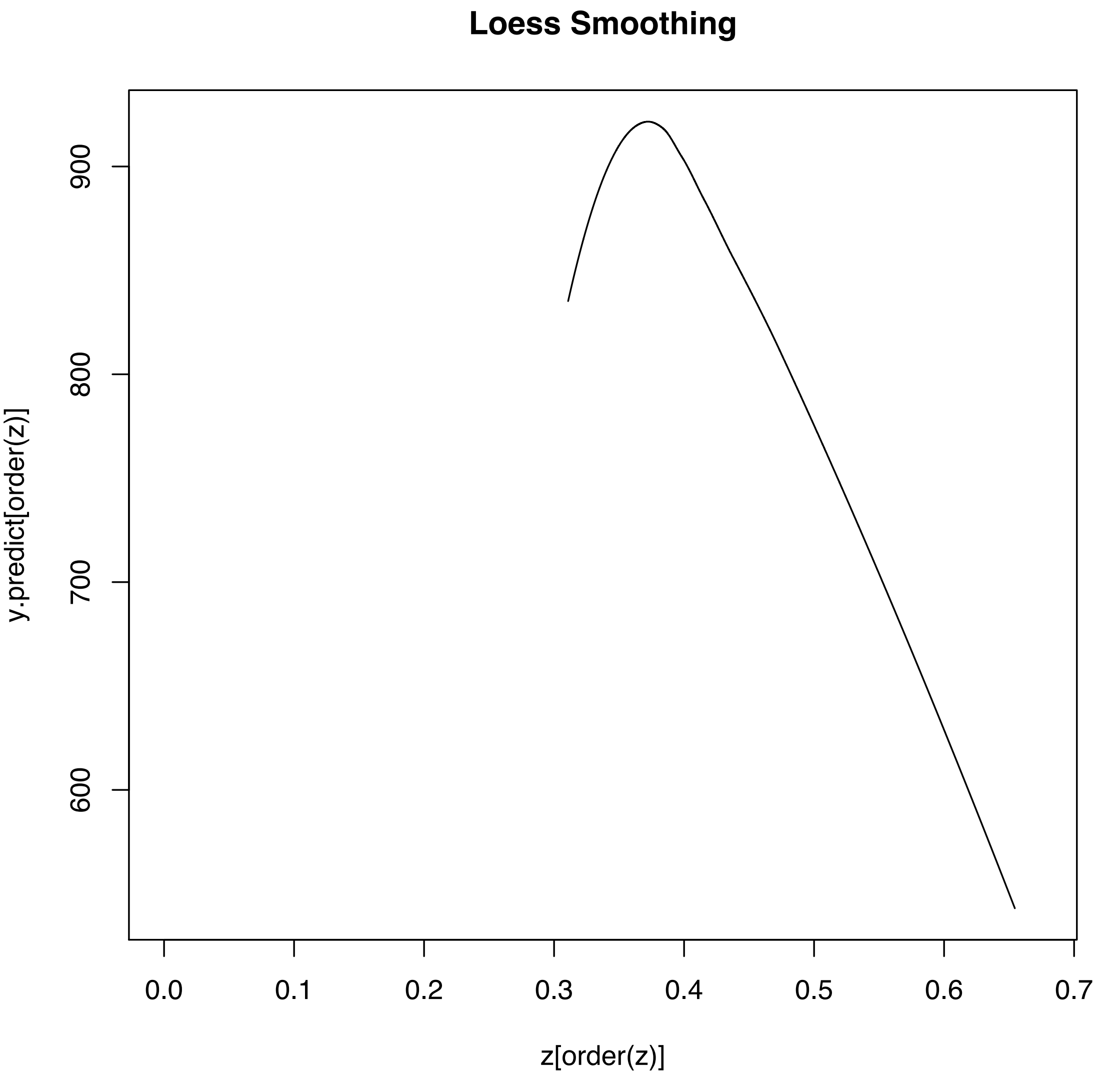

Loess Smoothing
y.predict[order(z)]
900
800
700
600
0.0
0.1
0.2
0.3
0.4
0.5
0.6
0.7
z[order(z)]

PL1307

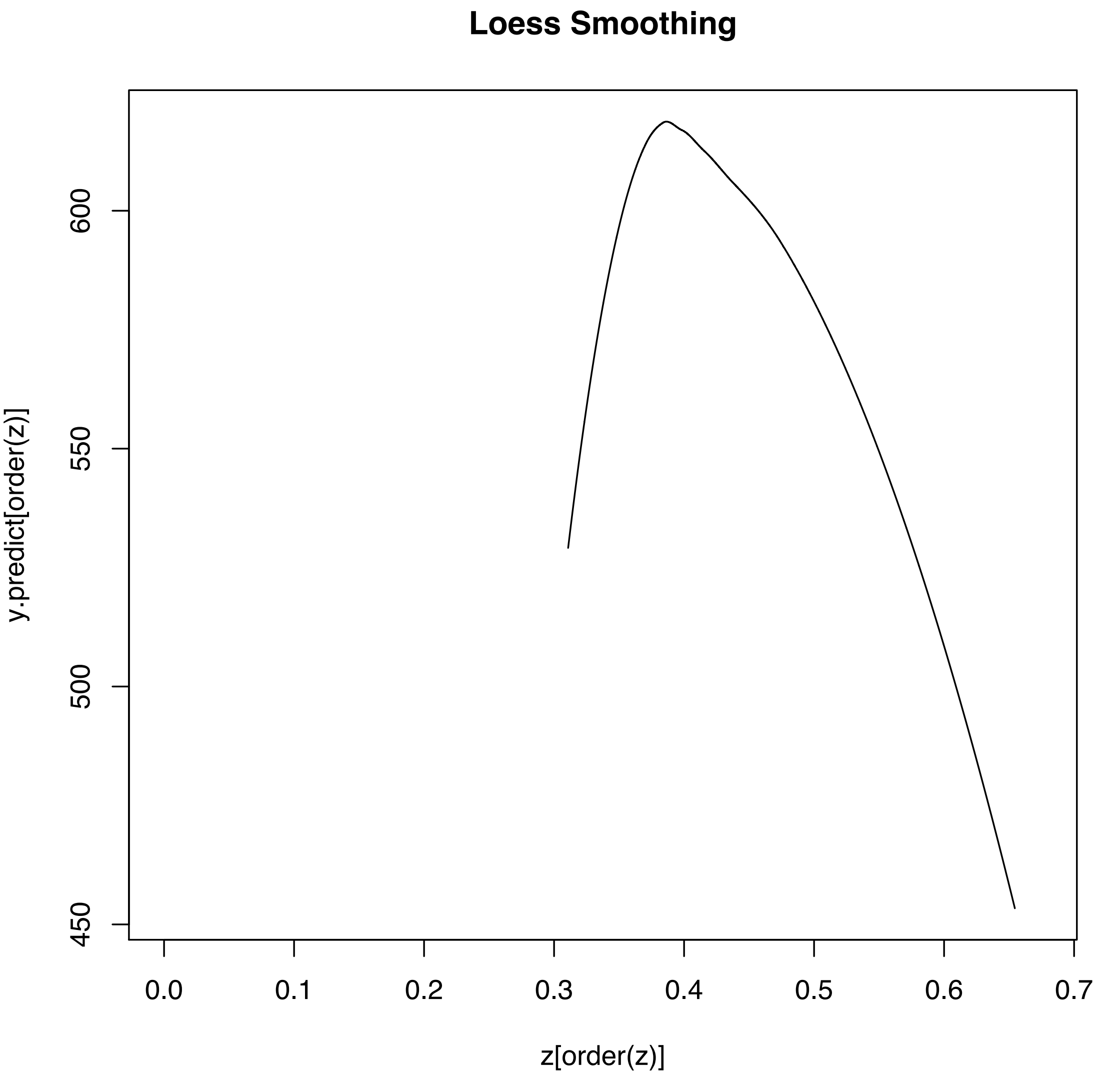

Loess Smoothing
600
550
500
450
y.predict[order(z)]
0.0
0.1
0.2
0.3
0.4
0.5
0.6
0.7
z[order(z)]

PL1303

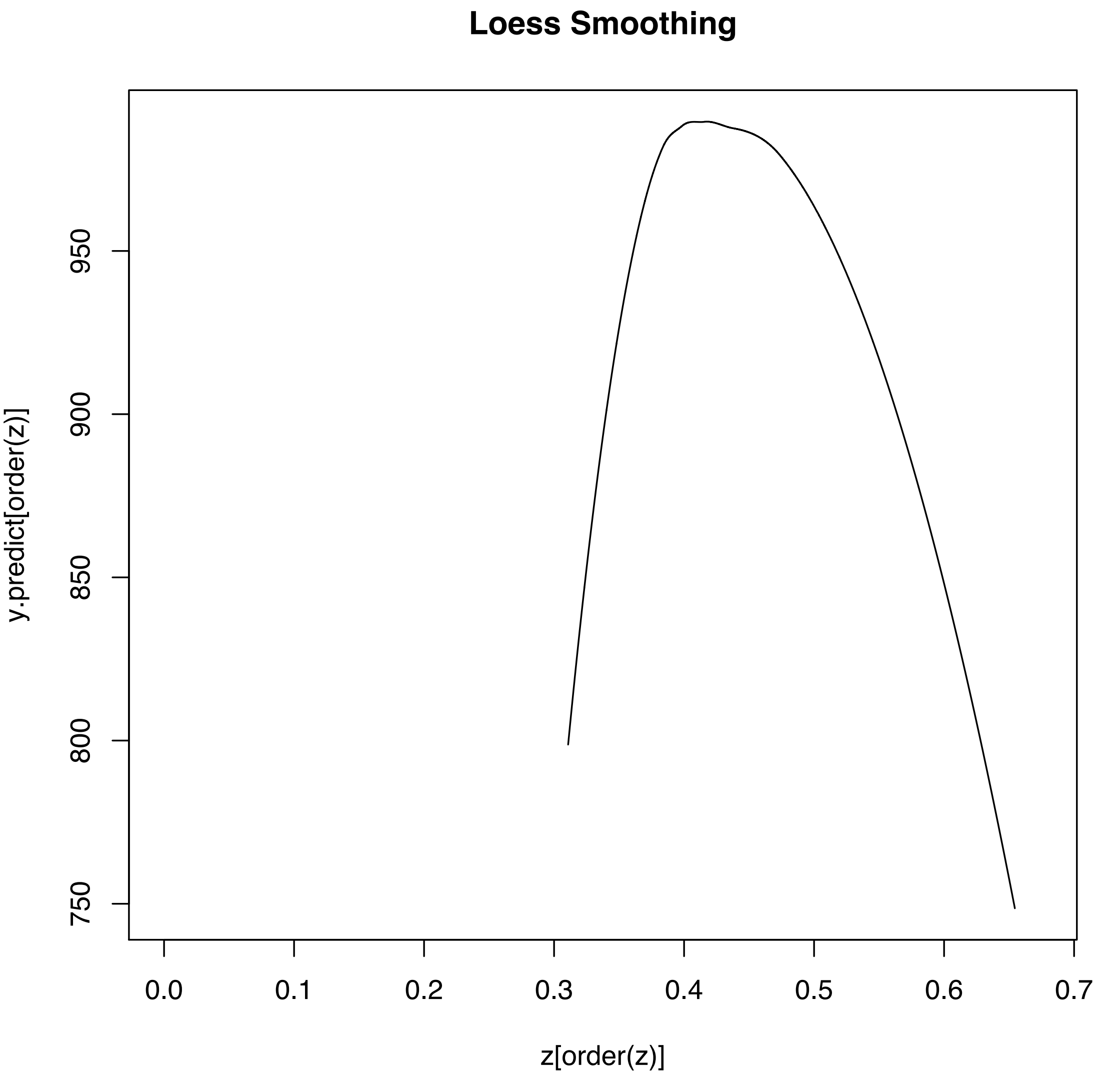
Loess Smoothing
950
900
850
800
750
y.predict[order(z)]
0.0
0.1
0.2
0.3
0.4
0.5
0.6
0.7
z[order(z)]

PL1268

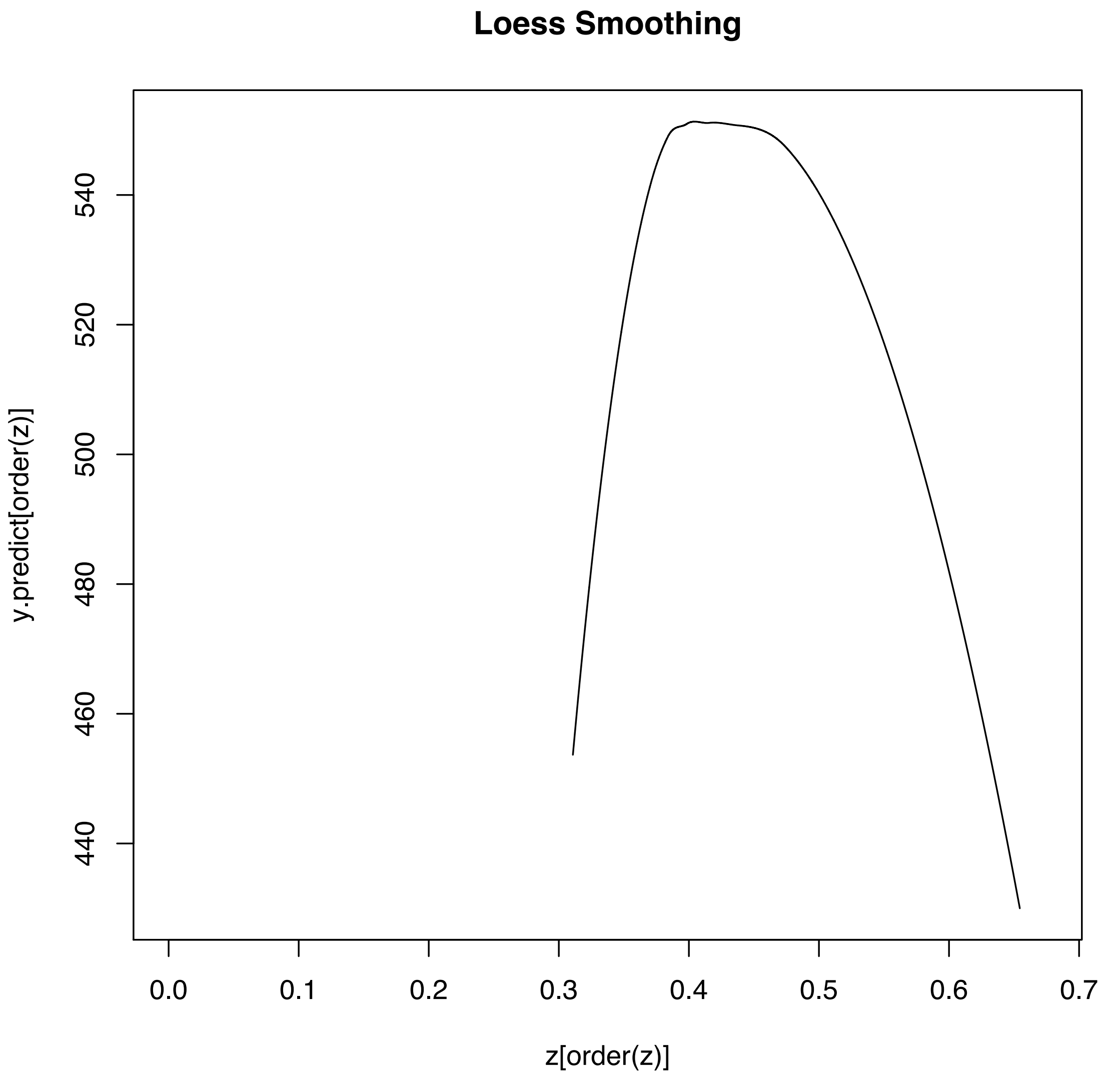

Loess Smoothing
y.predict[order(z)]
440
460
480
500
520
540
0.0
0.1
0.2
0.3
0.4
0.5
0.6
0.7
z[order(z)]

PL1265

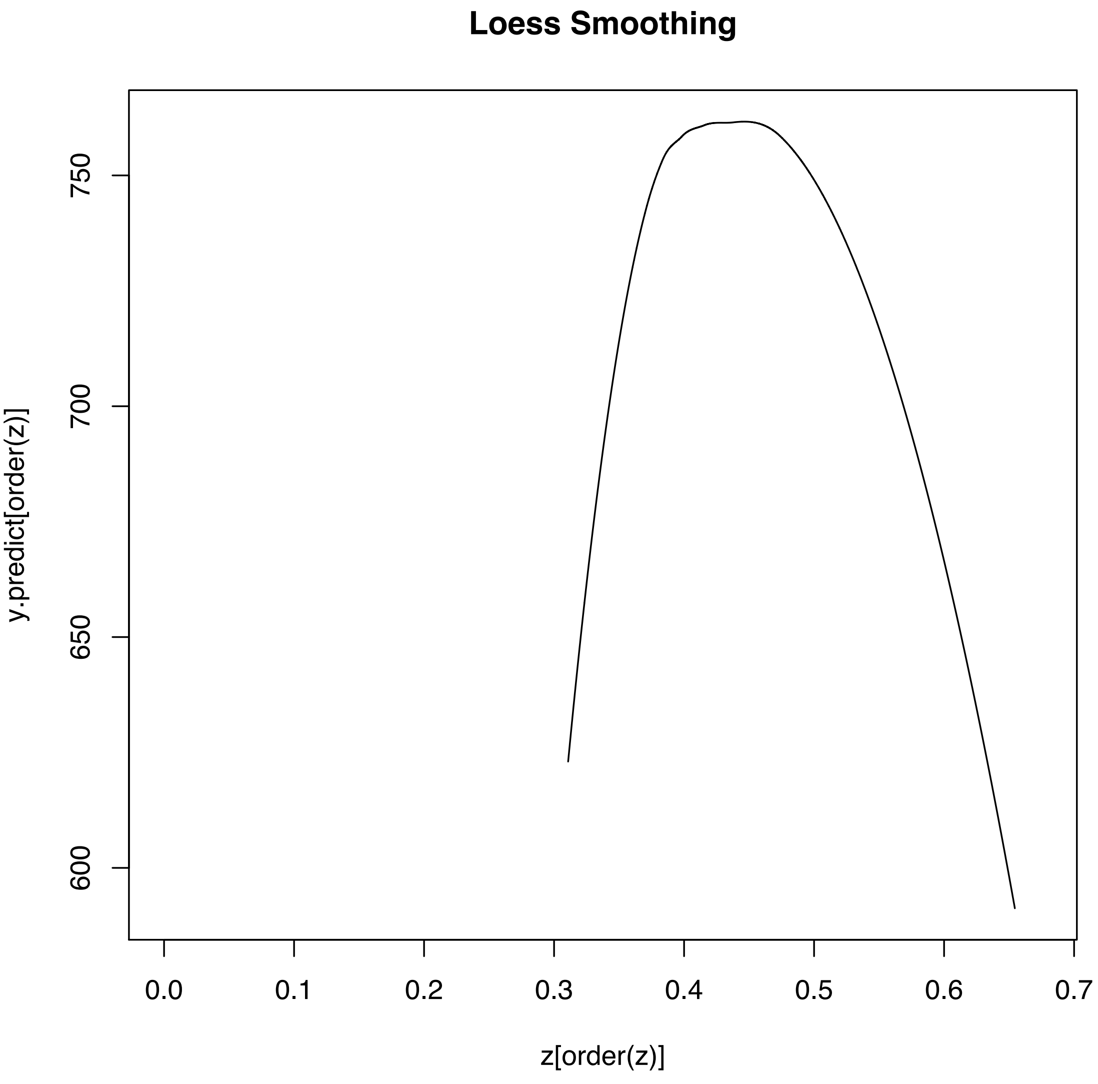

Loess Smoothing
750
700
650
600
y.predict[order(z)]
0.0
0.1
0.2
0.3
0.4
0.5
0.6
0.7
z[order(z)]

PL1236

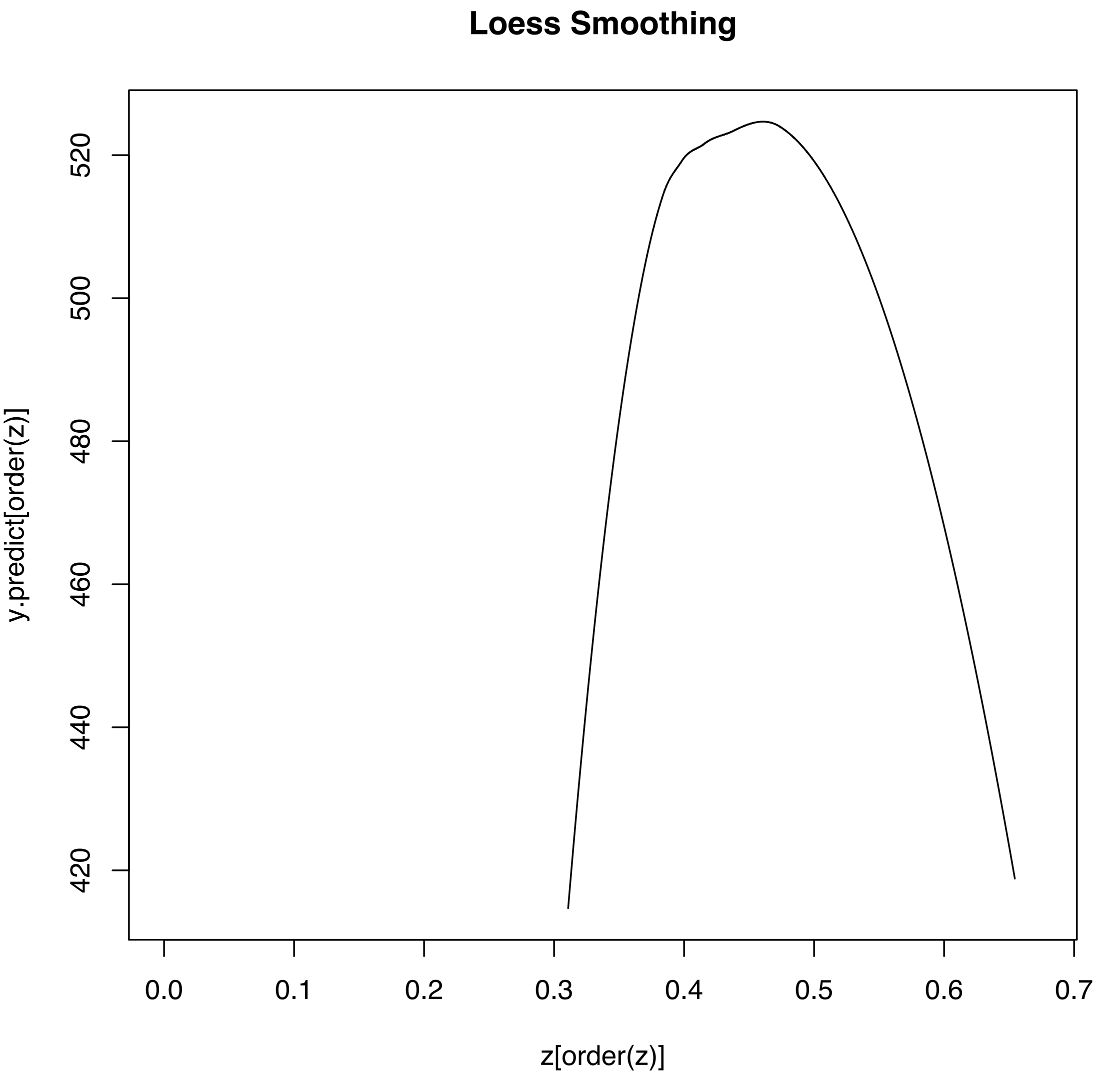

Loess Smoothing
520
500
480
460
440
420
y.predict[order(z)]
0.0
0.1
0.2
0.3
0.4
0.5
0.6
0.7
z[order(z)]

PL1157

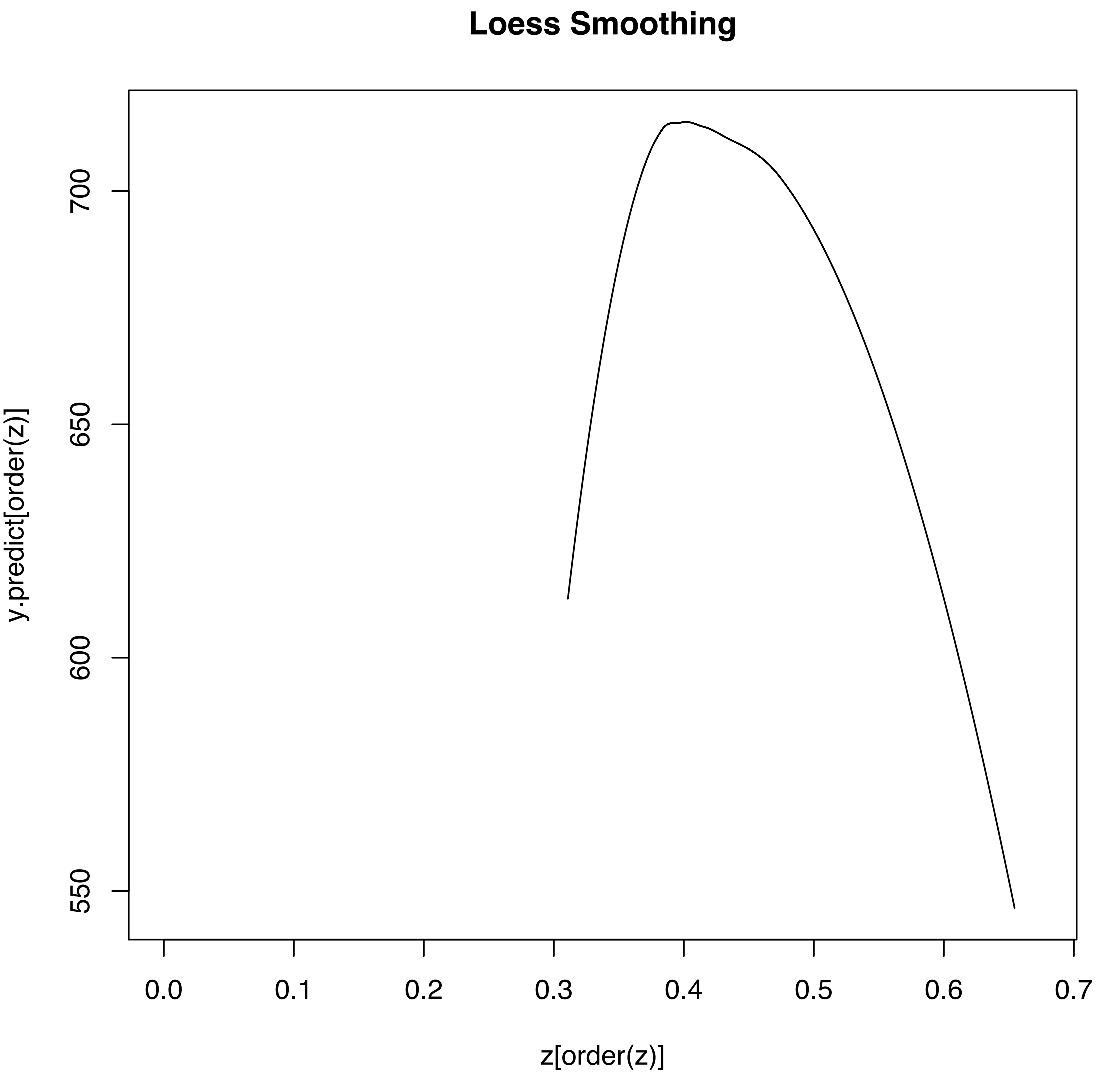

Loess Smoothing
y.predict[order(z)]
700
650
600
550
0.0
0.1
0.2
0.3
0.4
0.5
0.6
0.7
z[order(z)]

PL1139

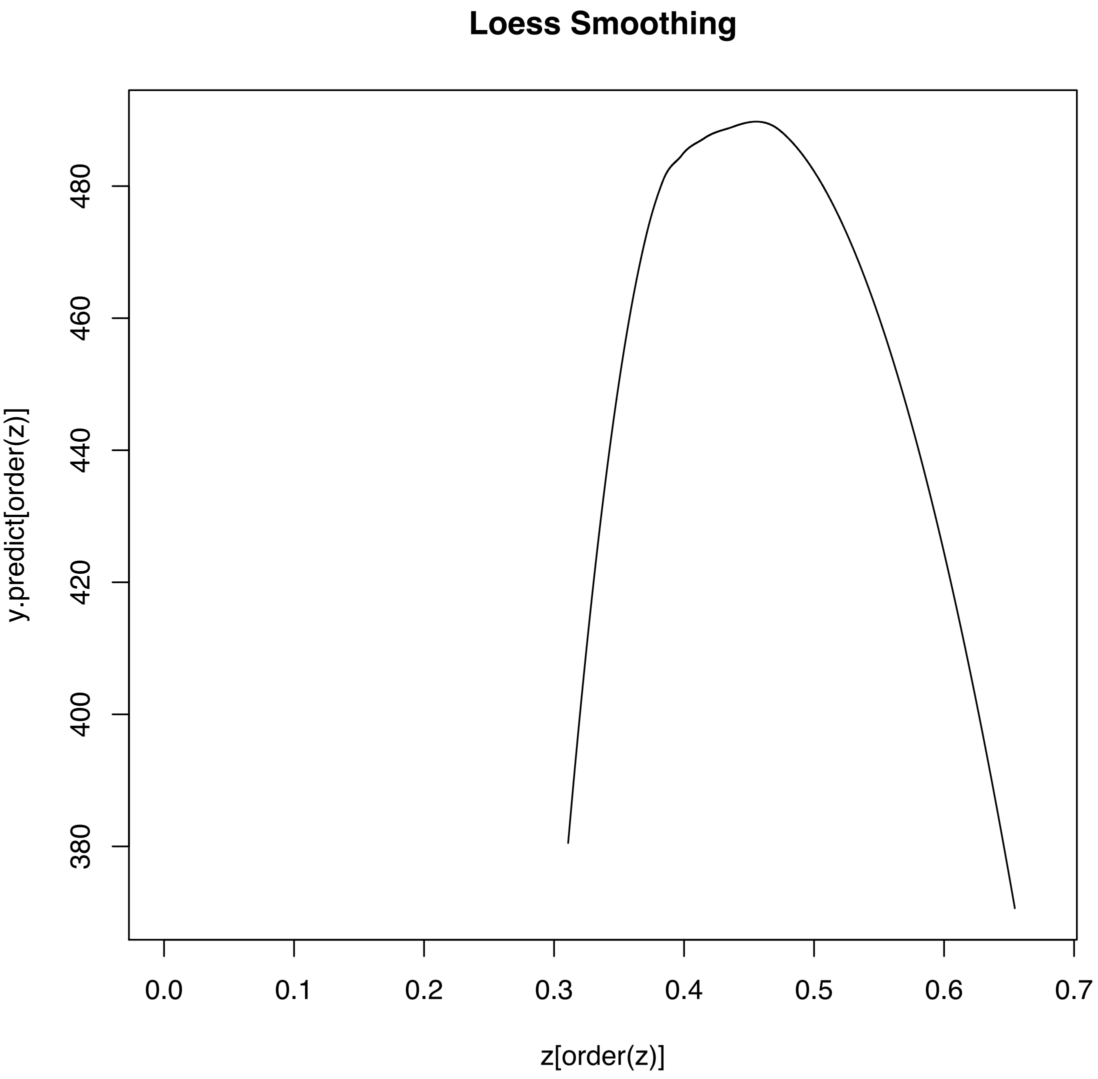

Loess Smoothing
y.predict[order(z)]
380
400
420
440
460
480
0.0
0.1
0.2
0.3
0.4
0.5
0.6
0.7
z[order(z)]

PL1090

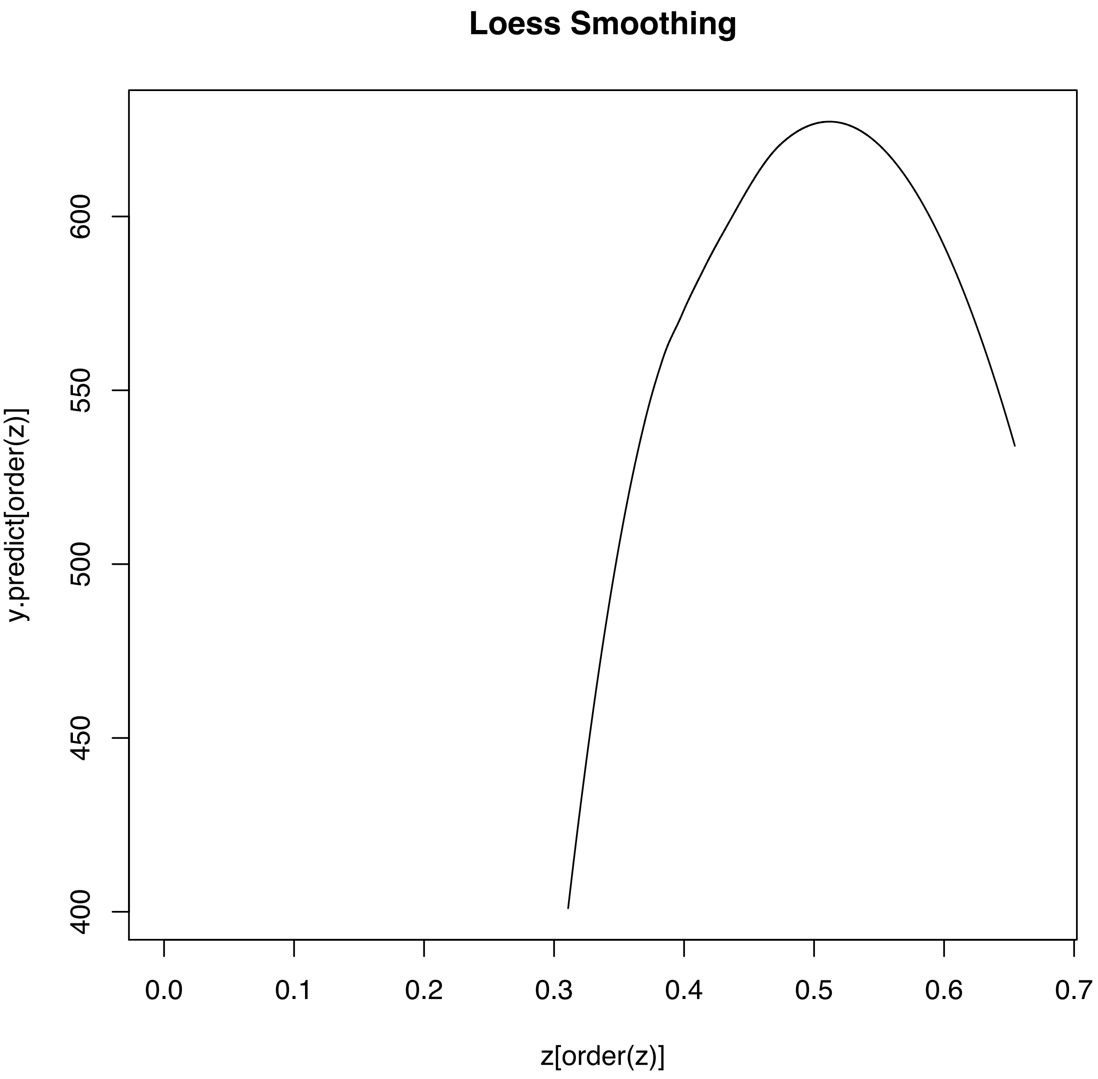

Loess Smoothing
600
550
500
450
400
y.predict[order(z)]
0.0
0.1
0.2
0.3
0.4
0.5
0.6
0.7
z[order(z)]

CVS201

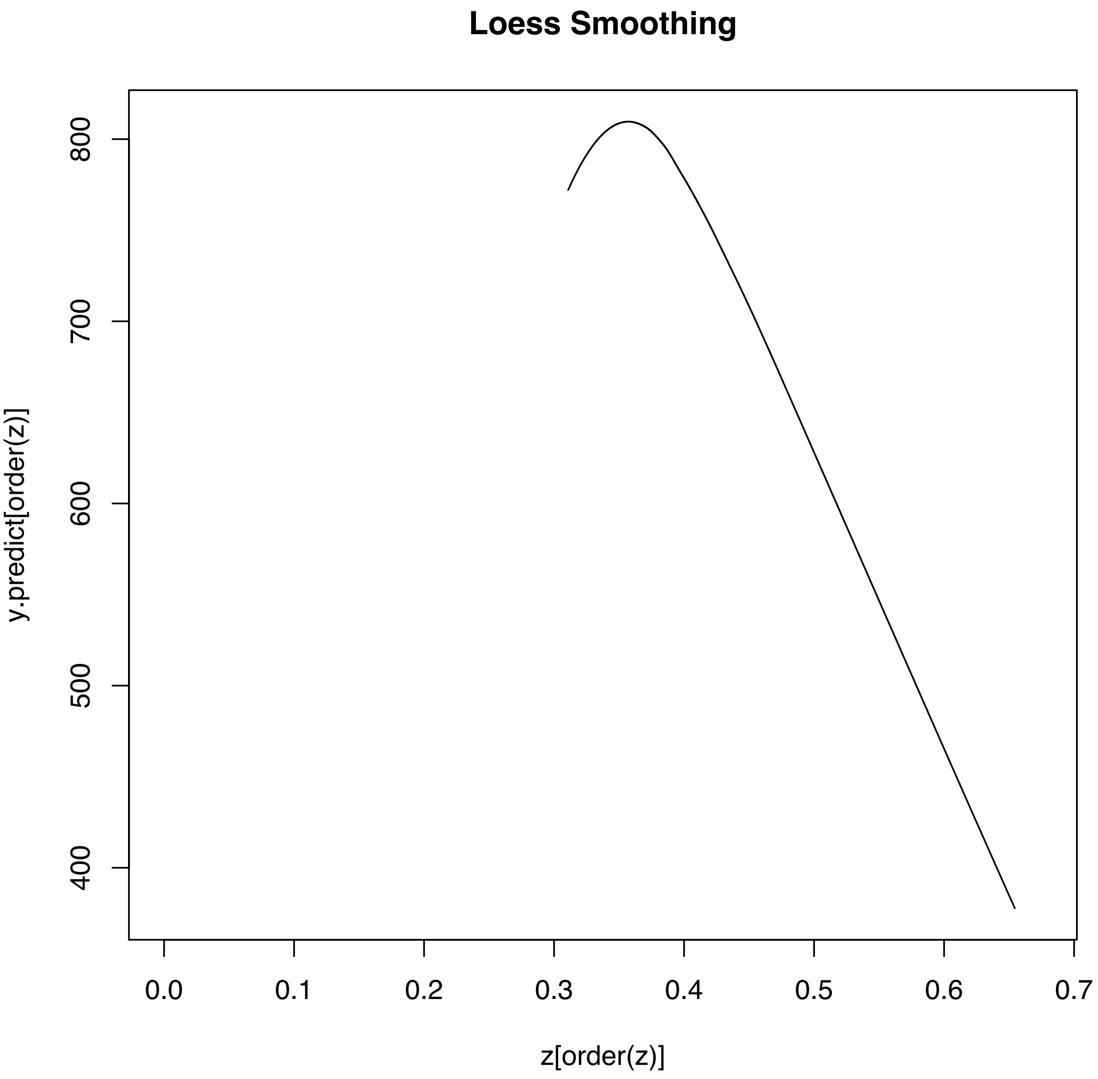

Loess Smoothing
800
700
600
500
400
y.predict[order(z)]
0.0
0.1
0.2
0.3
0.4
0.5
0.6
0.7
z[order(z)]

CVS200

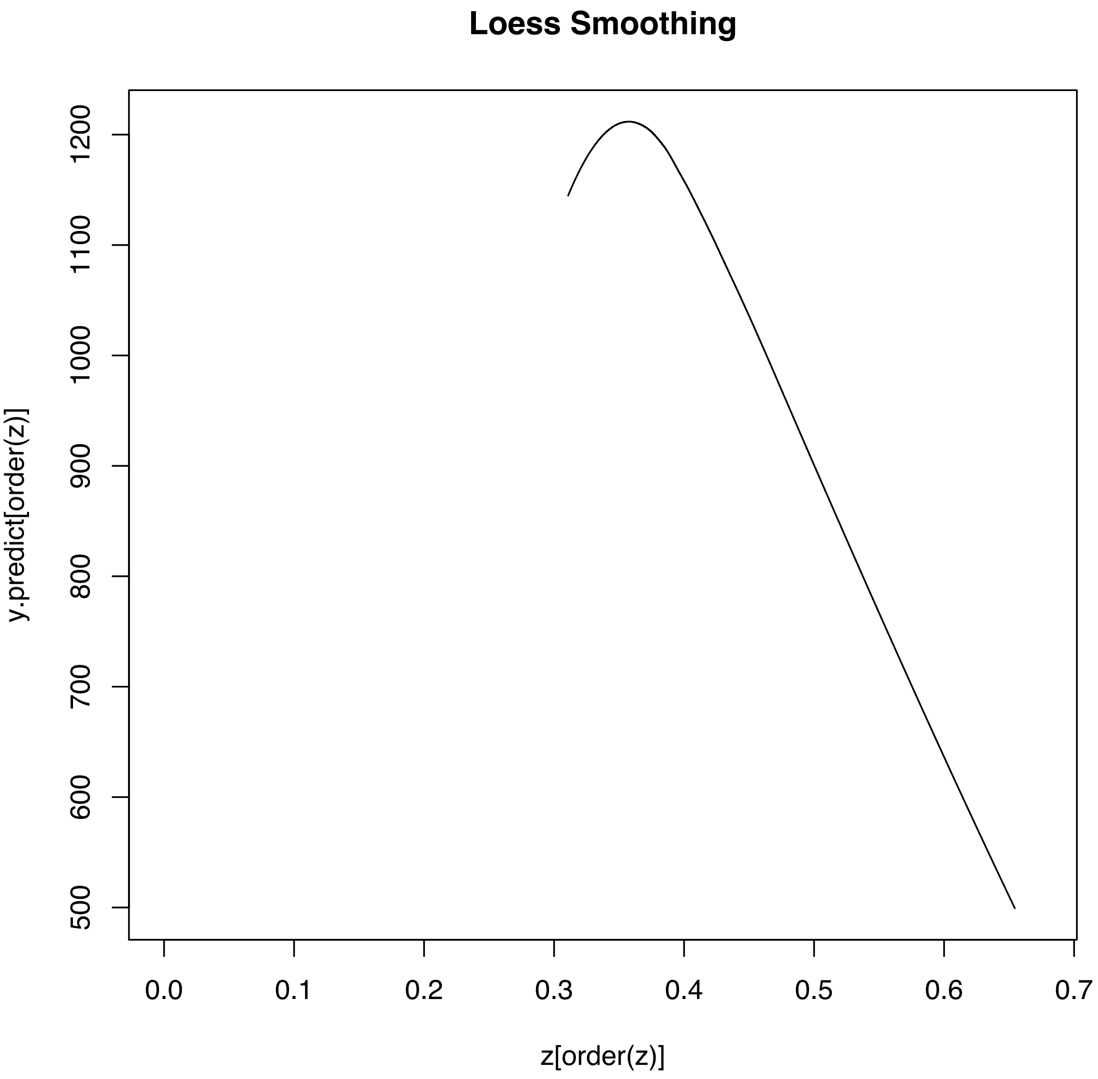

Loess Smoothing
y.predict[order(z)]
1200
1100
1000
900
800
700
600
500
0.0
0.1
0.2
0.3
0.4
0.5
0.6
0.7
z[order(z)]

CVS199

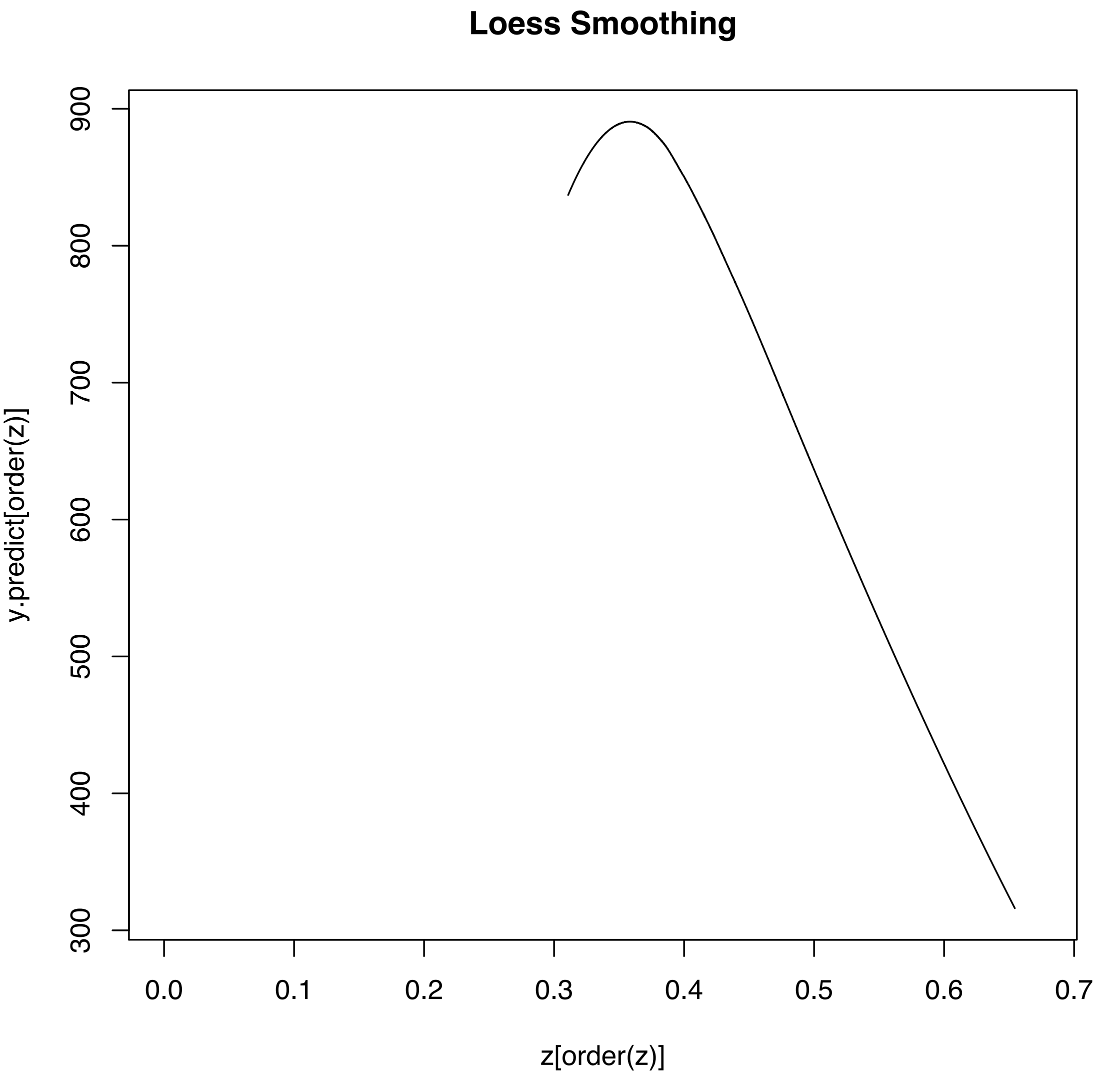
Loess Smoothing
y.predict[order(z)]
900
800
700
600
500
400
300
0.0
0.1
0.2
0.3
0.4
0.5
0.6
0.7
z[order(z)]

CVS185

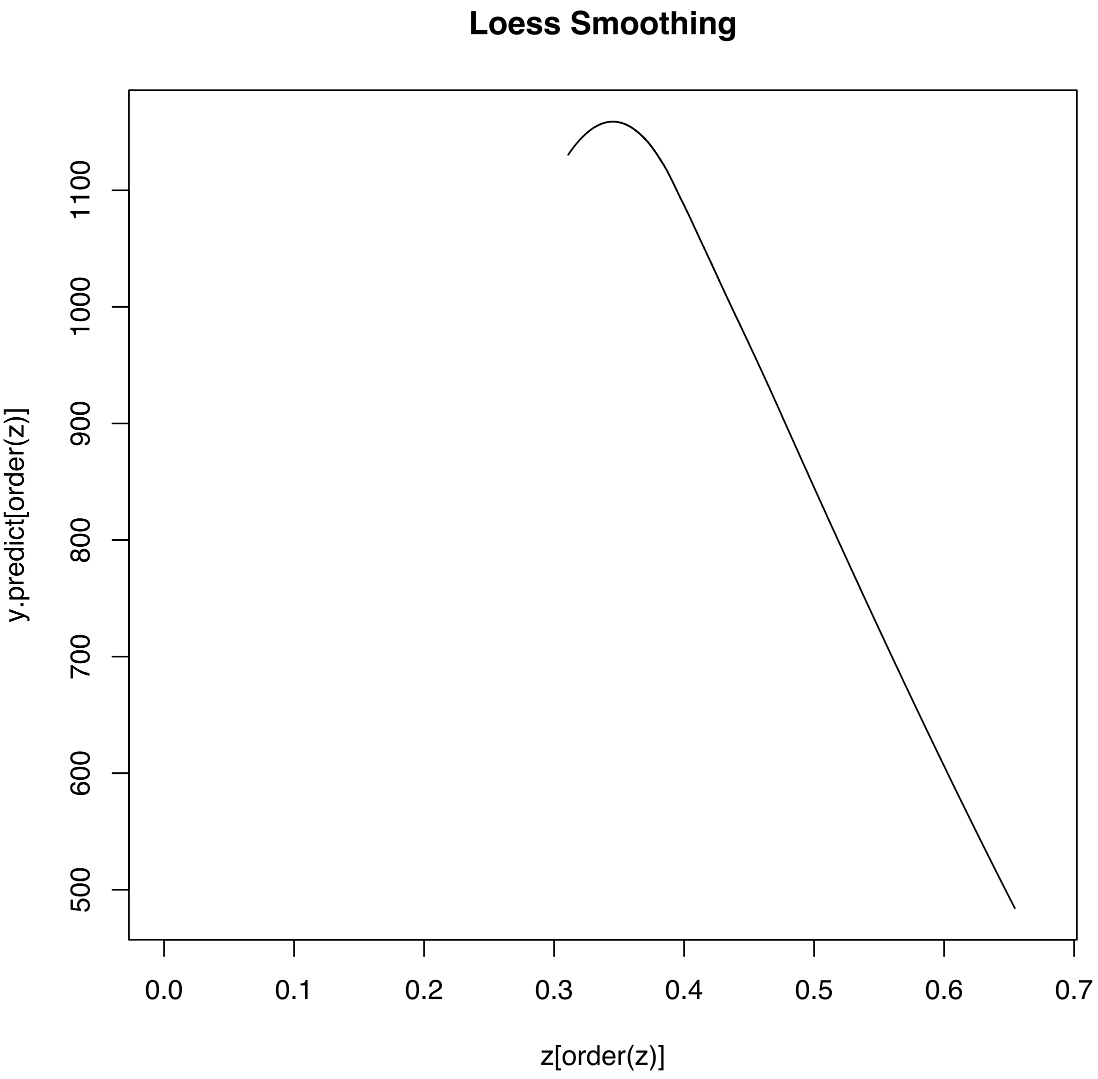
Loess Smoothing
y.predict[order(z)]
500
600
700
800
900
1000
1100
0.0
0.1
0.2
0.3
0.4
0.5
0.6
0.7
z[order(z)]

CVS184

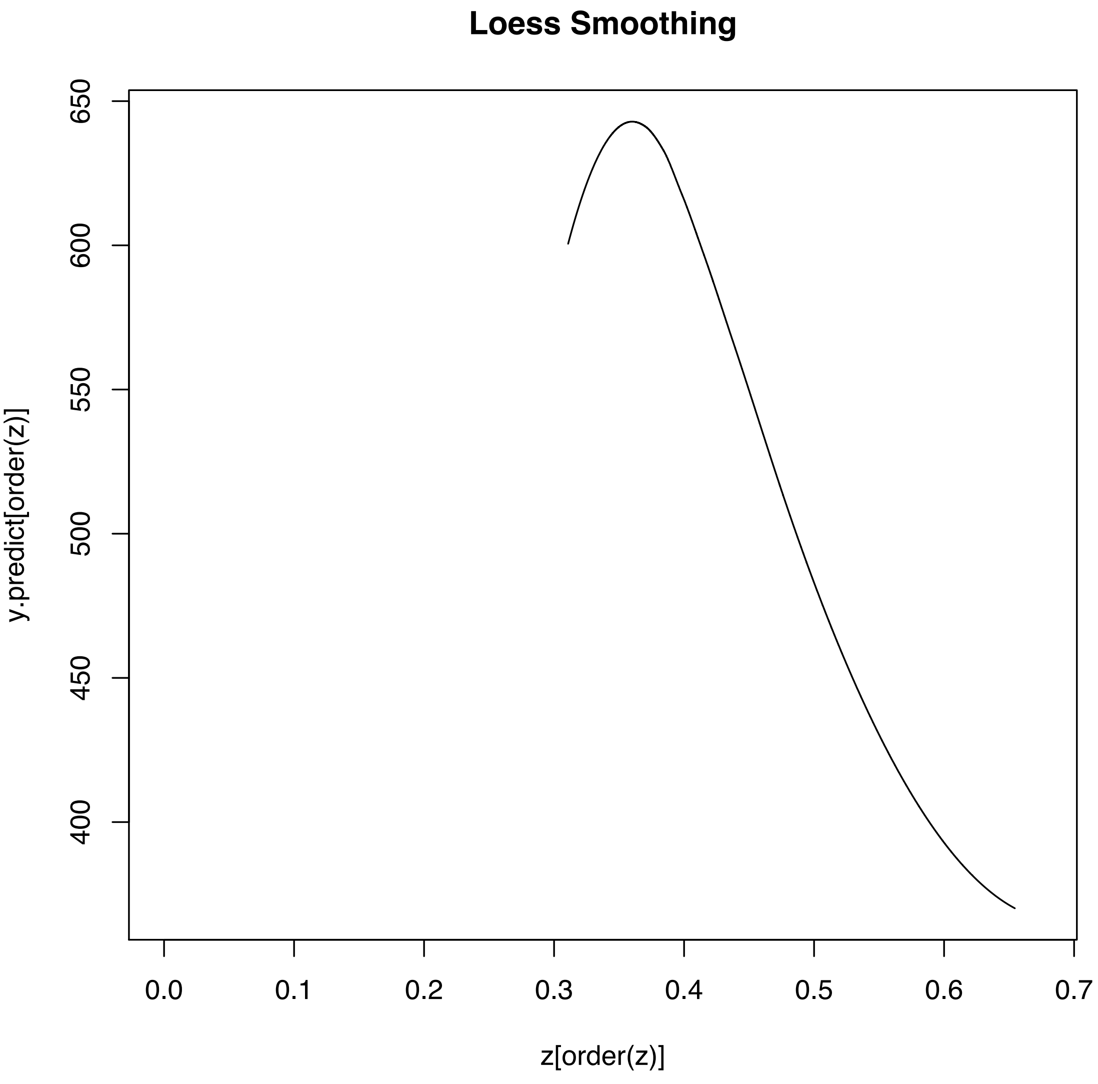

Loess Smoothing
650
600
550
500
450
400
y.predict[order(z)]
0.0
0.1
0.2
0.3
0.4
0.5
0.6
0.7
z[order(z)]

CVS183

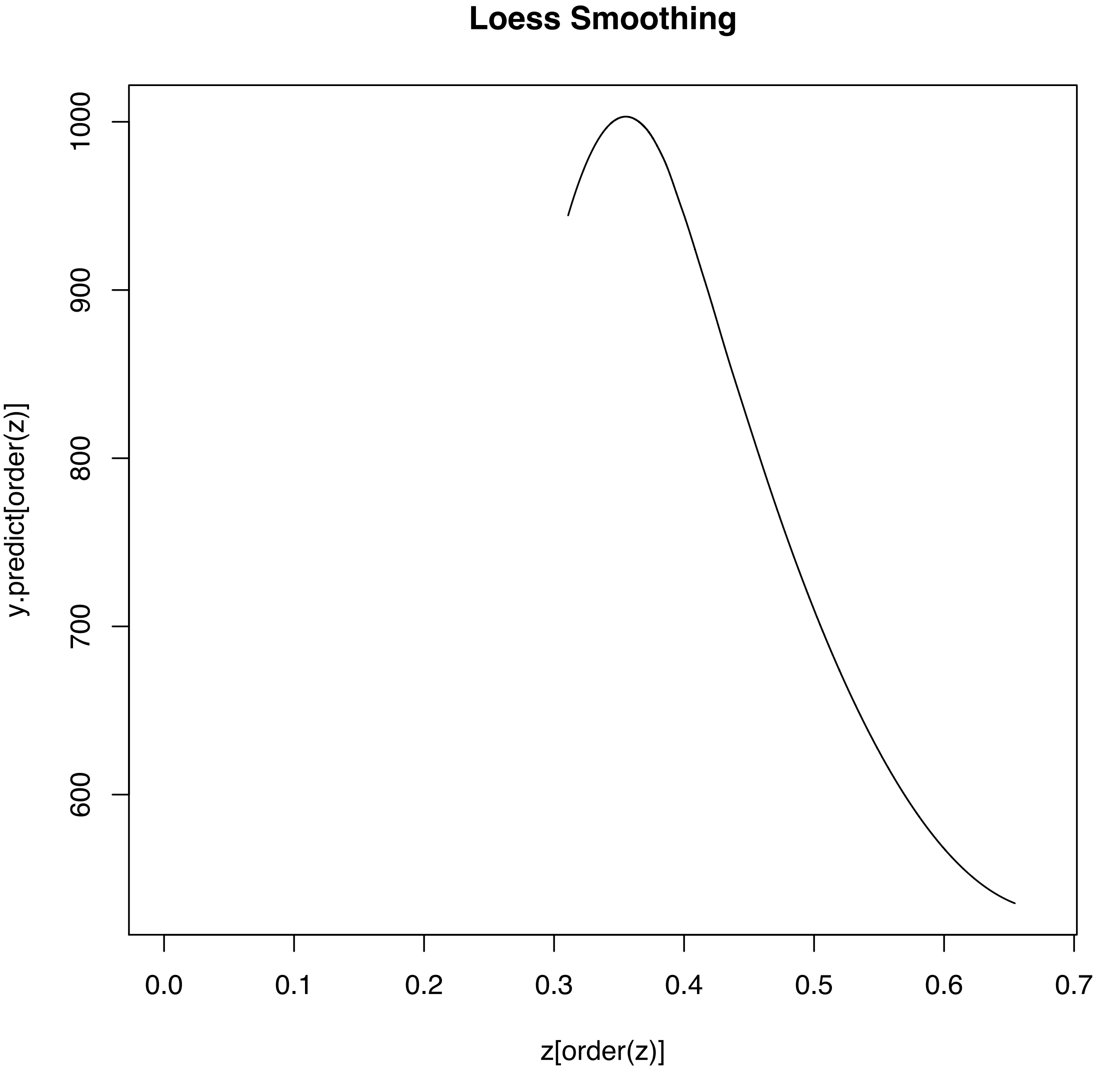
Loess Smoothing
y.predict[order(z)]
600
700
800
900
1000
0.0
0.1
0.2
0.3
0.4
0.5
0.6
0.7
z[order(z)]
