# Supplementary material for: High Levels of Sample-to-Sample Variation Confound Data Analysis for Non-Invasive Prenatal Screening of Fetal Microdeletions
Source: PLoS One. 2016 Jun 1;11(6):e0153182. doi: 10.1371/journal.pone.0153182 (PMC4889033; doi:10.1371/journal.pone.0153182)
Supplement: S2 Fig — There are 22 groups of plots in this file for the 22 autosomes. Each group has three plots. In each group, the first plot is the cv.RTC of the 50kb regions in the CVS control libraries against that in the maternal plasma control libraries for each autosome. The second plot is the cv.RTC of the 50kb regions in the plasma control libraries against the log2(GVF+1) of the 50kb regions reported in the DGV database. The third plot is the cv.RTC of the CVS control libraries against the DGV database. (PDF) [file pone.0153182.s002.pdf]

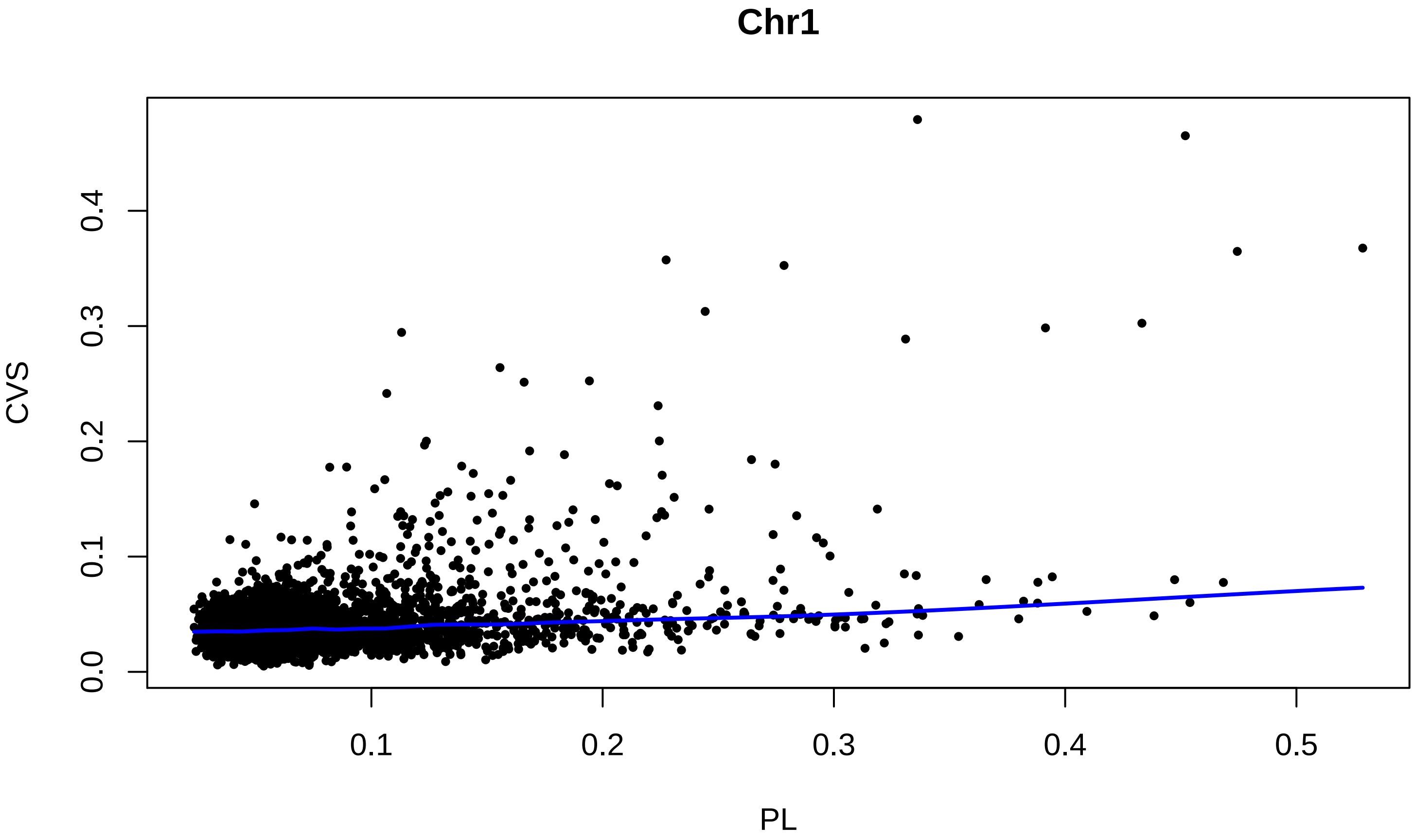
Chr1
CVS
PL
0.0
0.1
0.2
0.3
0.4
0.1
0.2
0.3
0.4
0.5

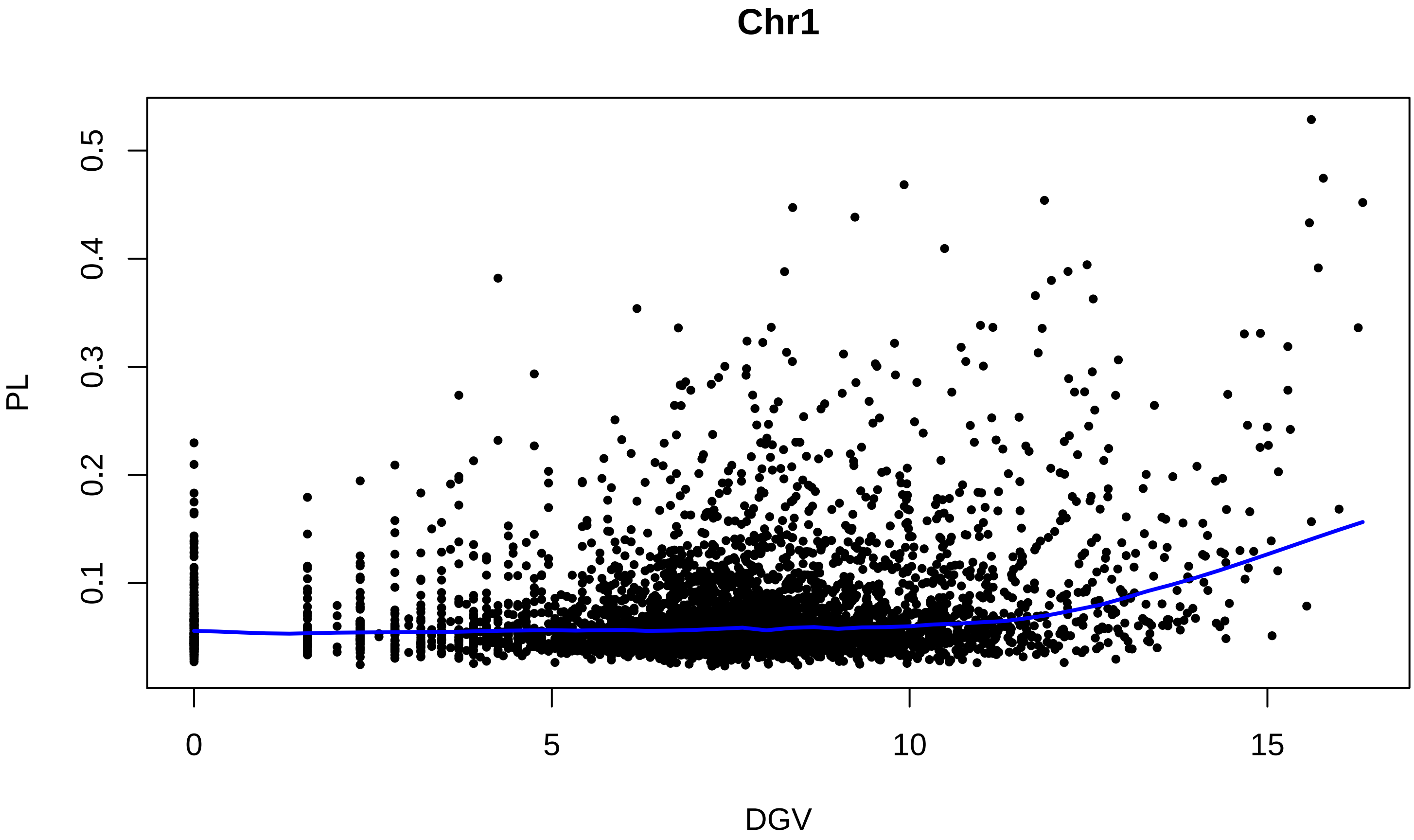
Chr1
PL
DGV
0.1
0.2
0.3
0.4
0.5
0
5
10
15

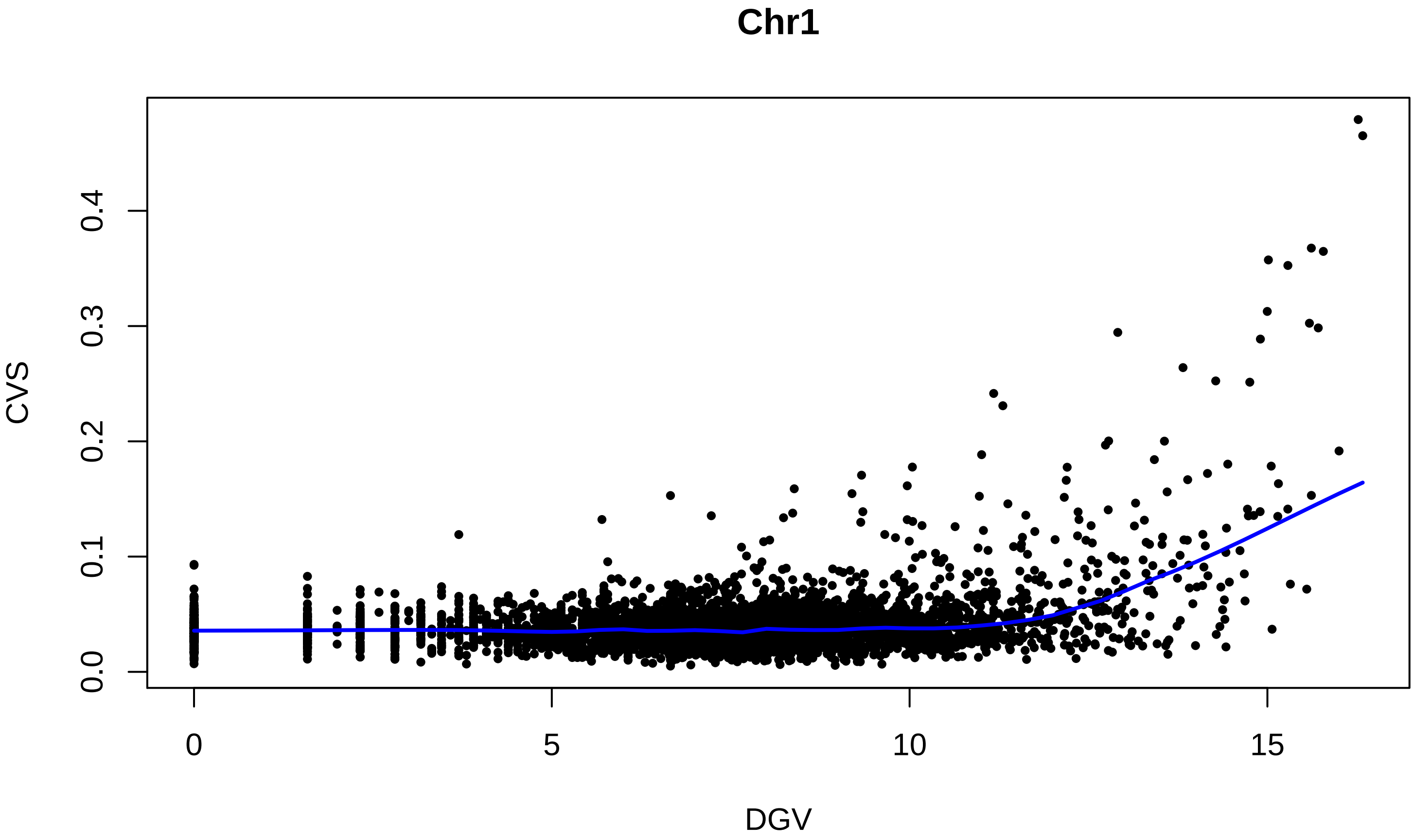
Chr1
CVS
0.0
0.1
0.2
0.3
0.4
DGV
0
5
10
15

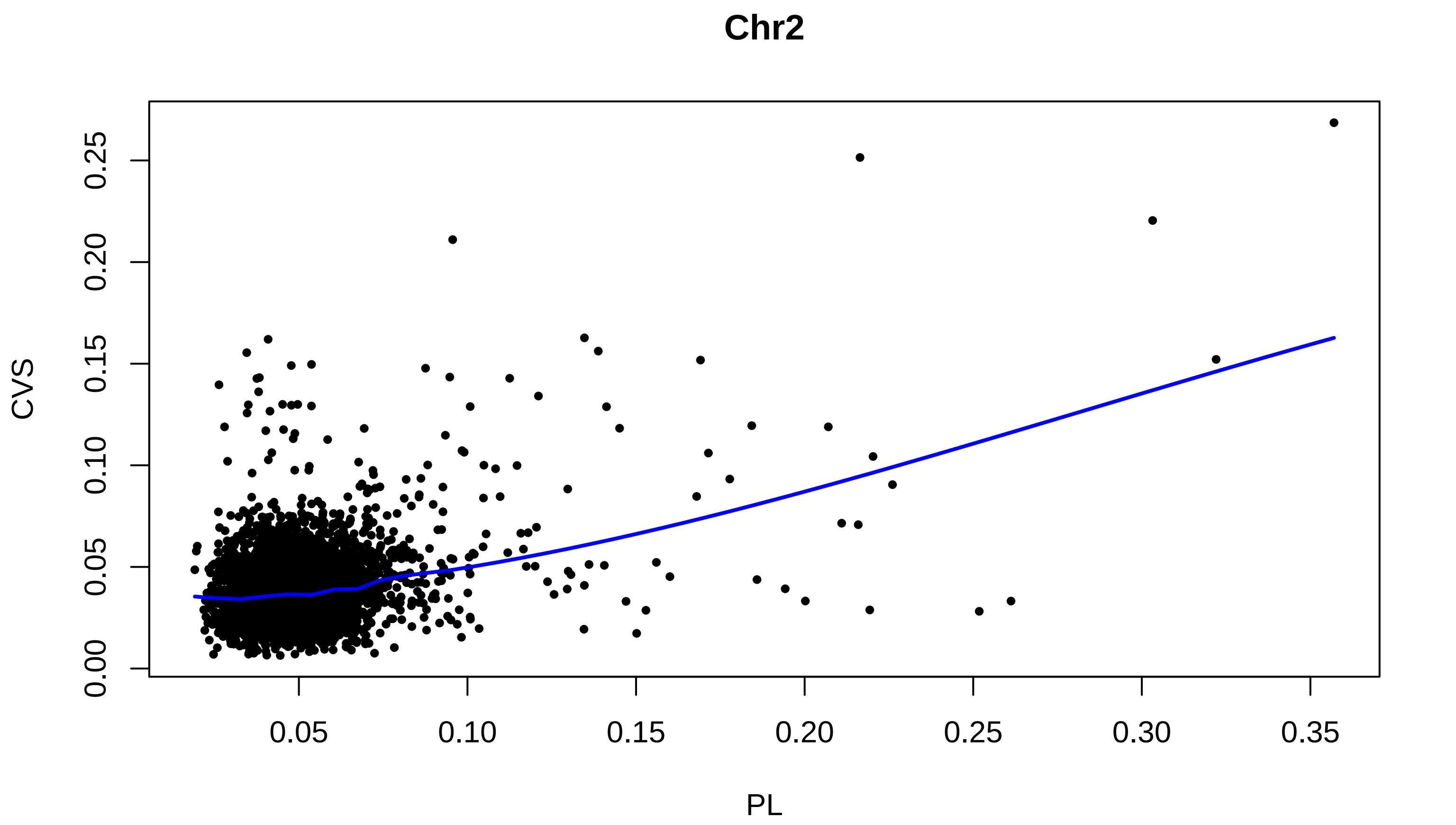

Chr2
CVS
PL
0.00
0.05
0.10
0.15
0.20
0.25
0.05
0.10
0.15
0.20
0.25
0.30
0.35

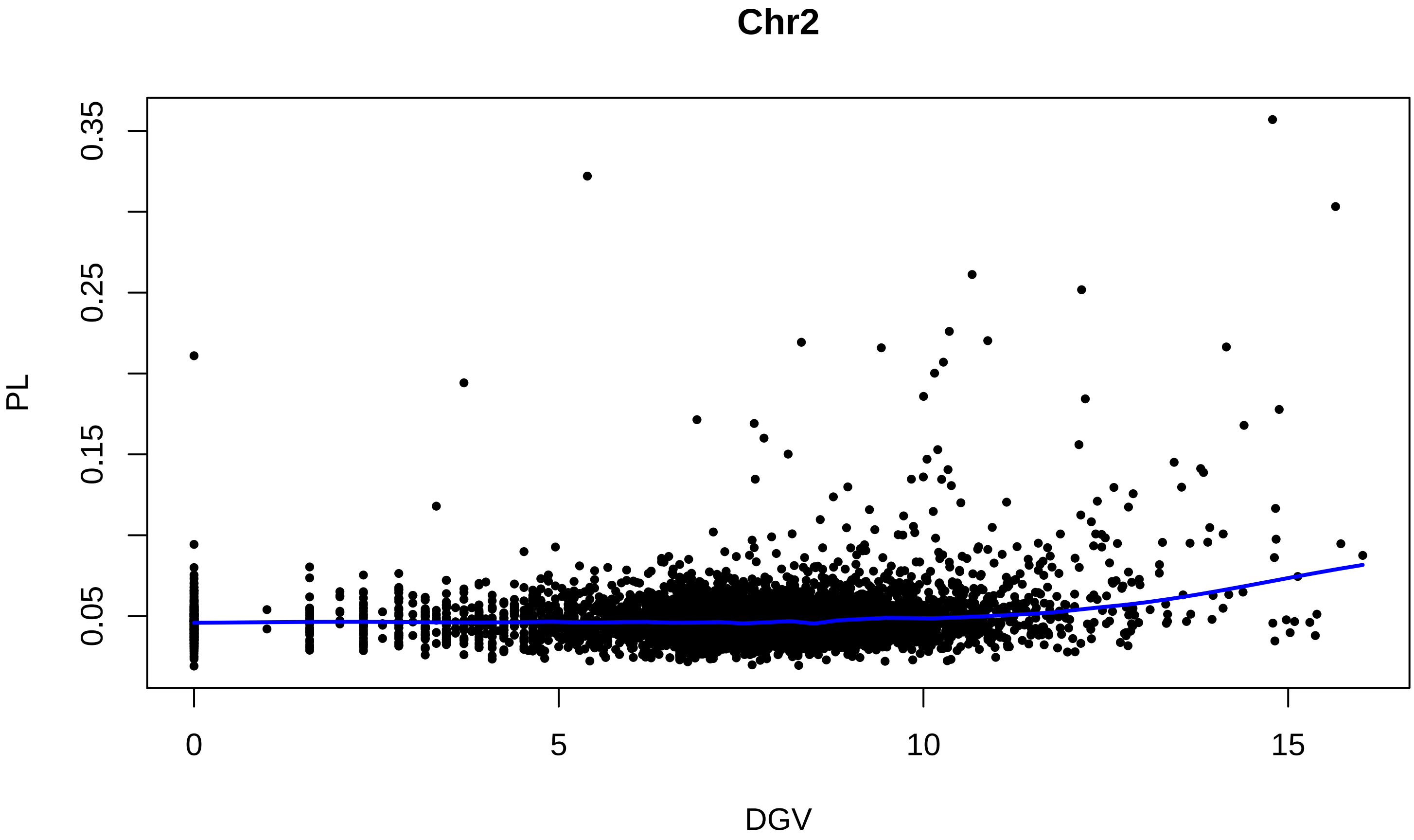
Chr2
PL
DGV
0.05
0.15
0.25
0.35
0
5
10
15

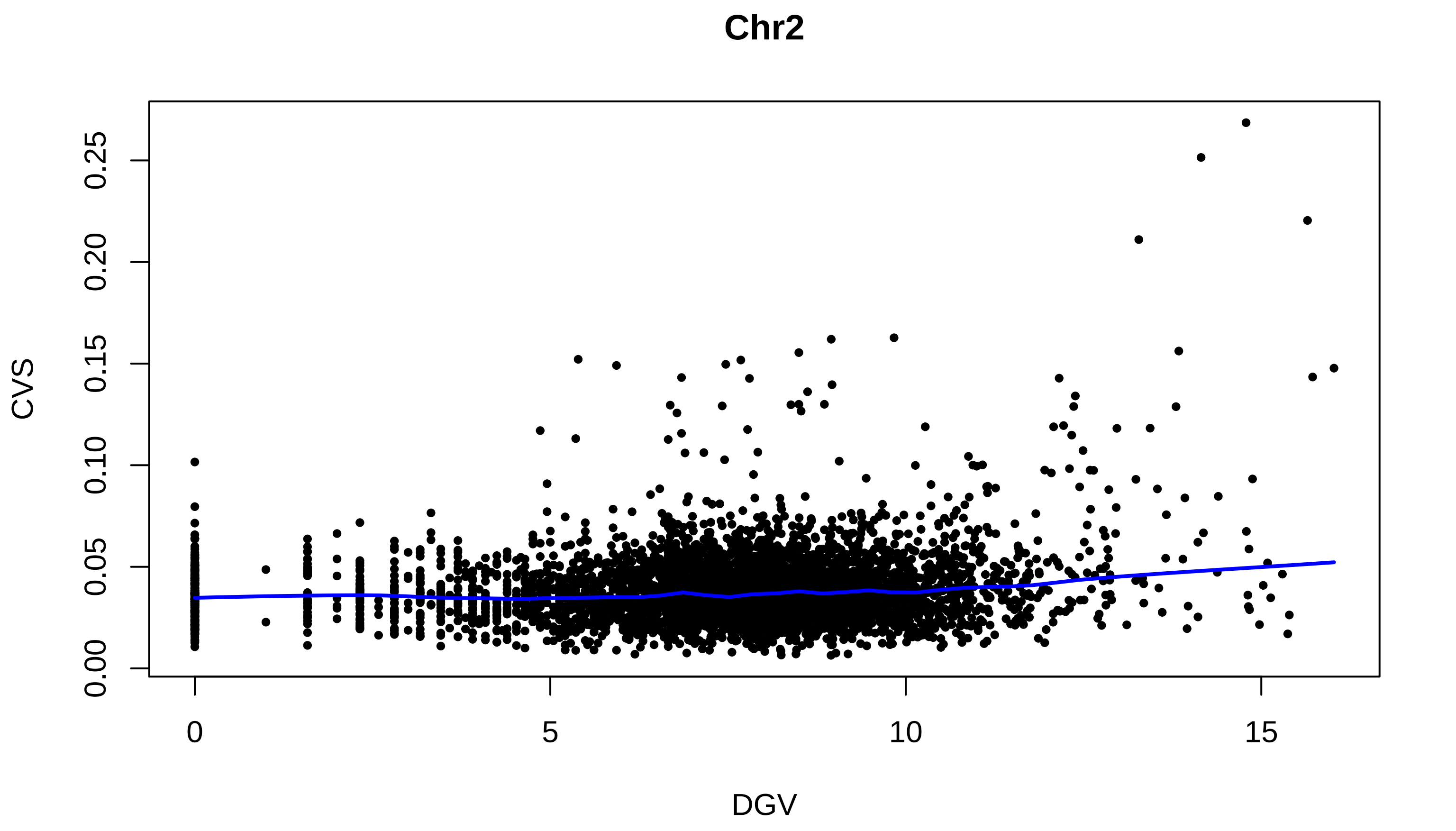

Chr2
CVS
DGV
0.00
0.05
0.10
0.15
0.20
0.25
0
5
10
15

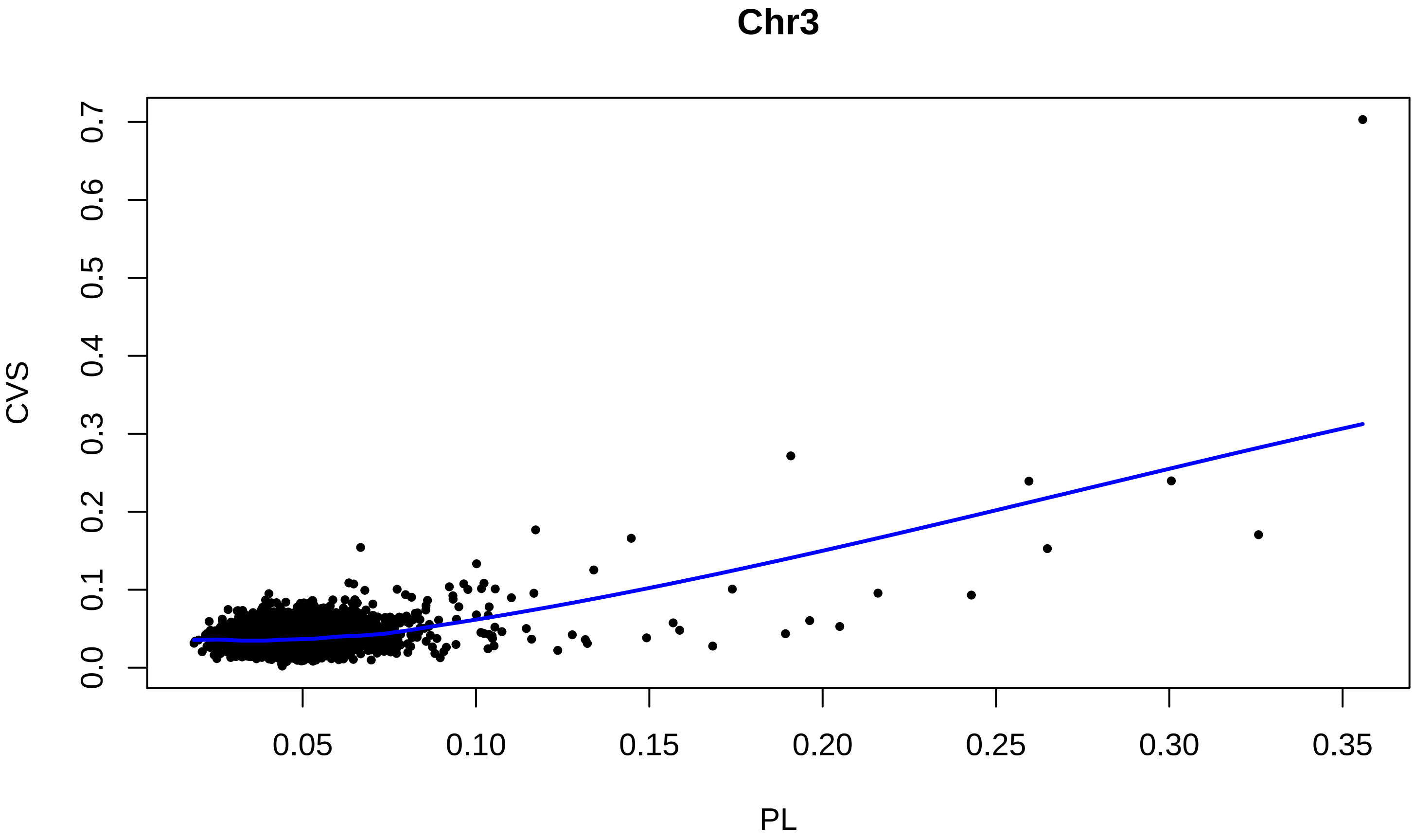

Chr3
CVS
PL
0.0
0.1
0.2
0.3
0.4
0.5
0.6
0.7
0.05
0.10
0.15
0.20
0.25
0.30
0.35

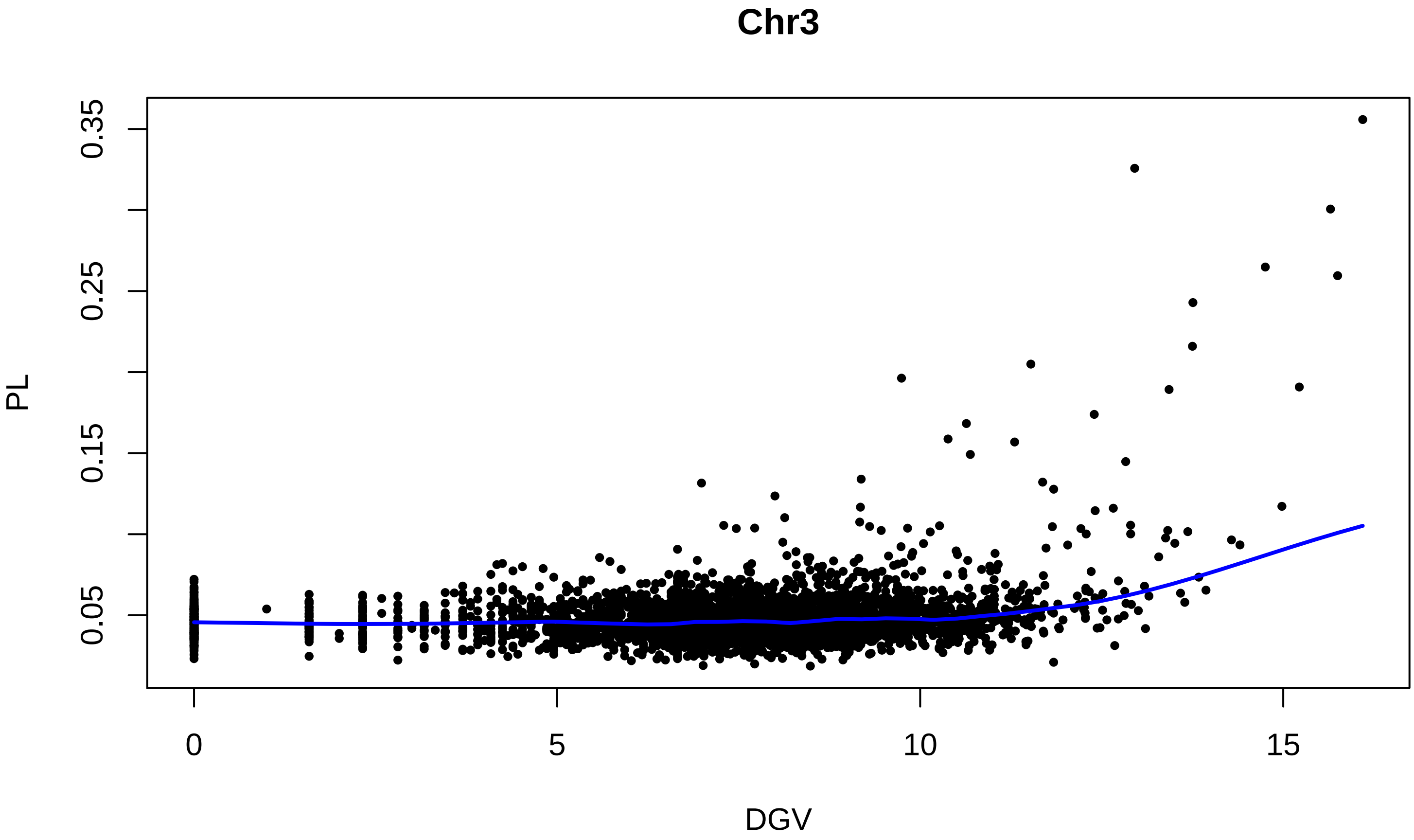
Chr3
PL
0.35
0.25
0.15
0.05
DGV
0
5
10
15

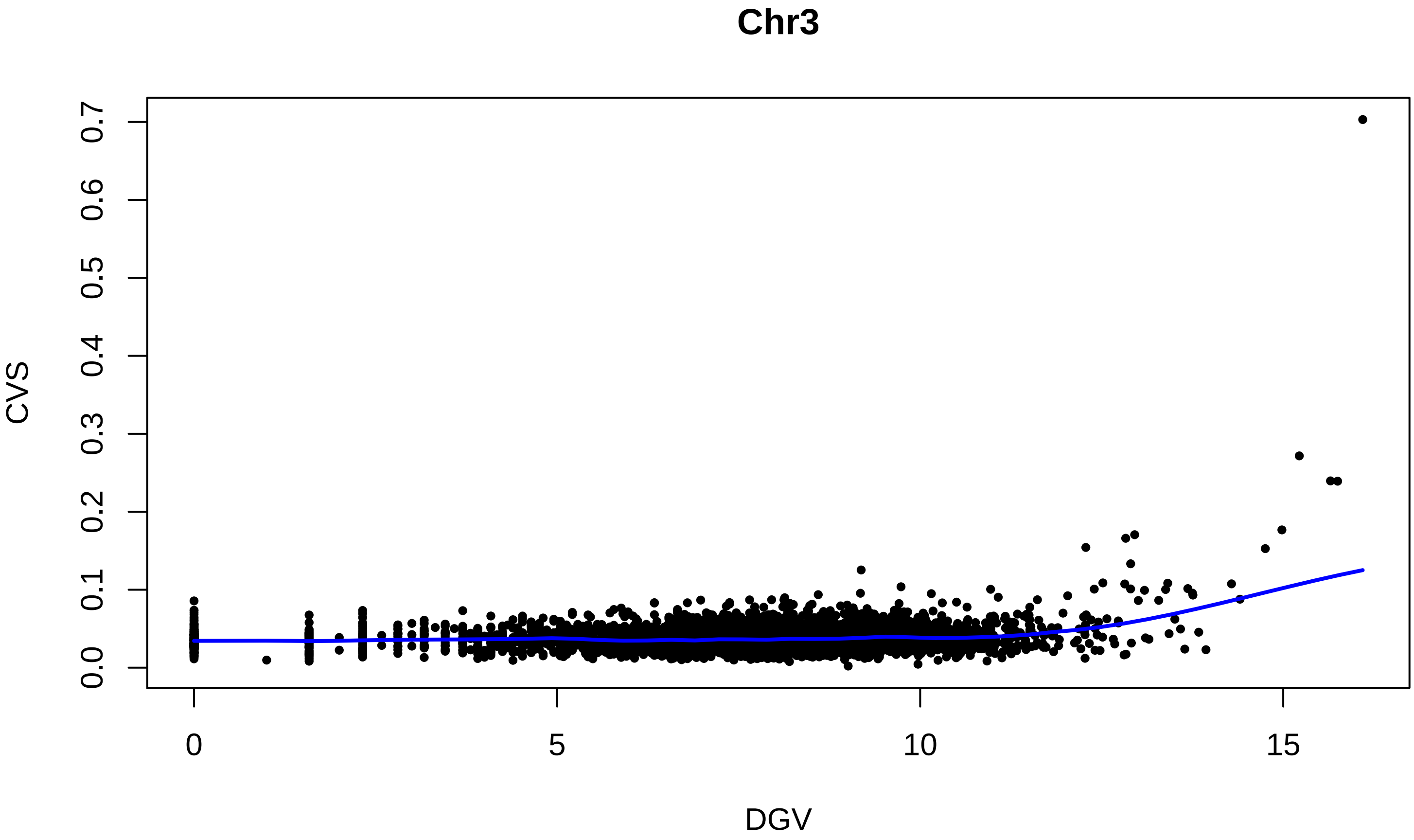
Chr3
CVS
DGV
0.0
0.1
0.2
0.3
0.4
0.5
0.6
0.7
0
5
10
15

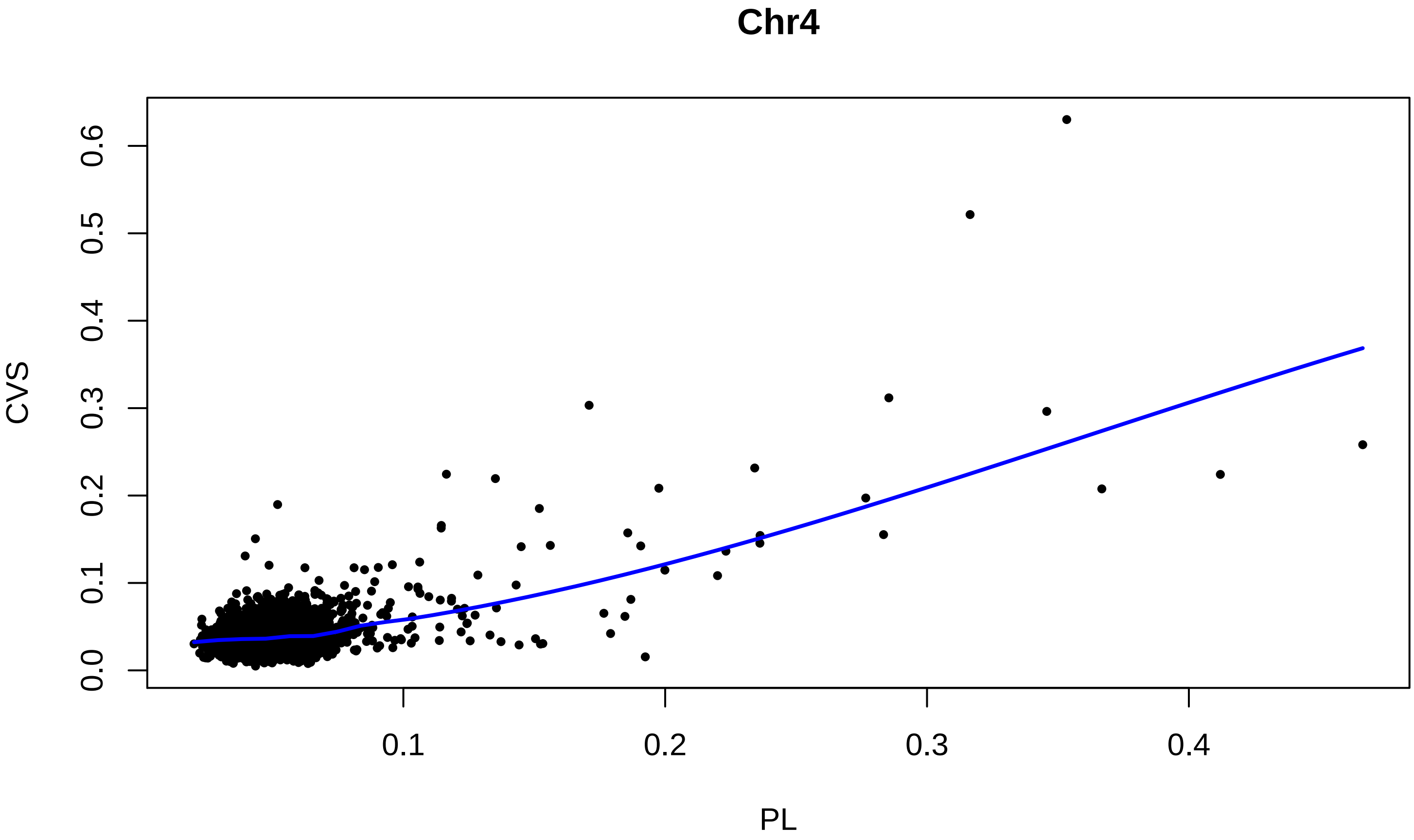
Chr4
CVS
PL
0.0
0.1
0.2
0.3
0.4
0.5
0.6
0.1
0.2
0.3
0.4

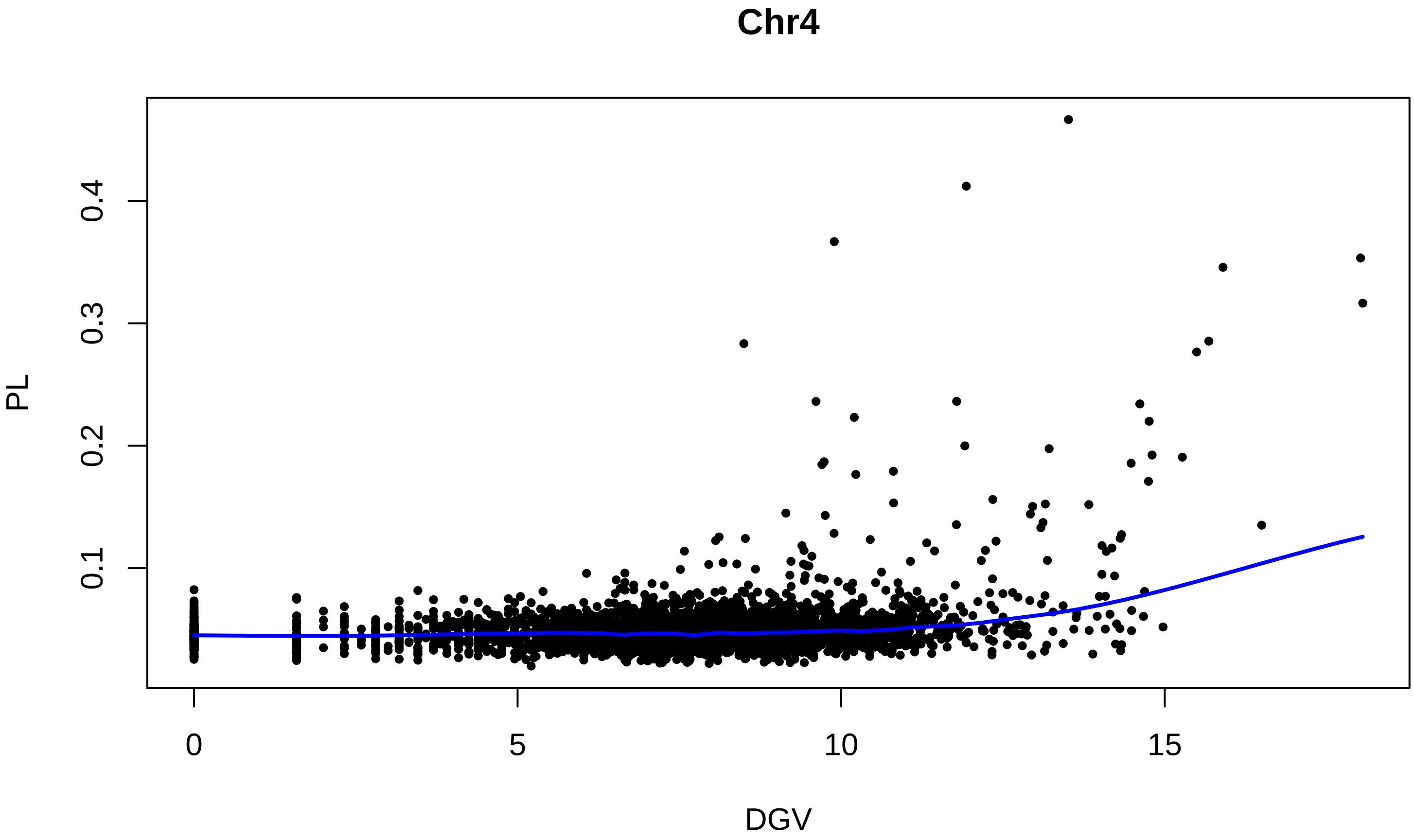
Chr4
PL
0.1
0.2
0.3
0.4
0
5
10
15
DGV

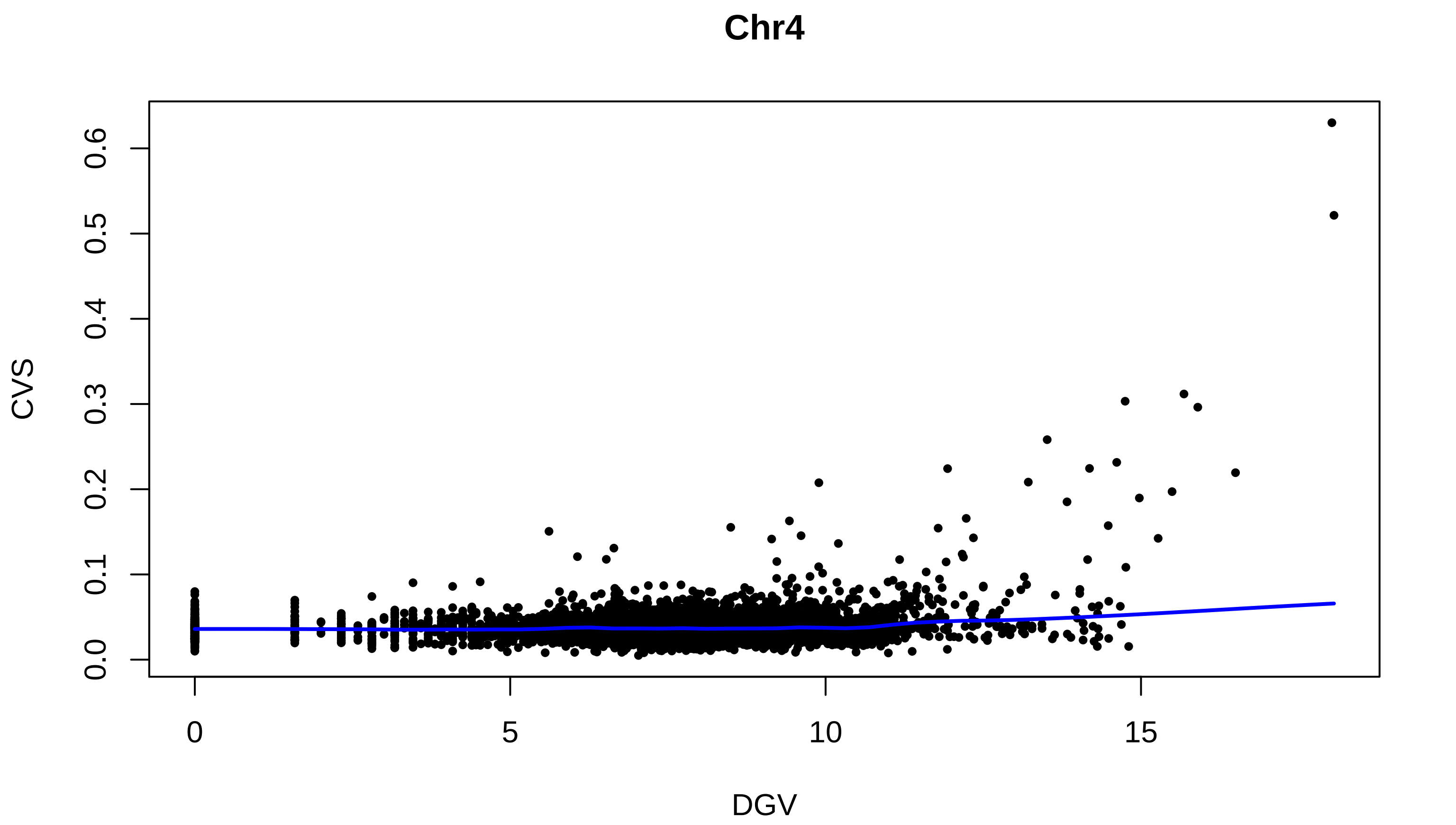
Chr4
CVS
DGV
0.0
0.1
0.2
0.3
0.4
0.5
0.6
0
5
10
15

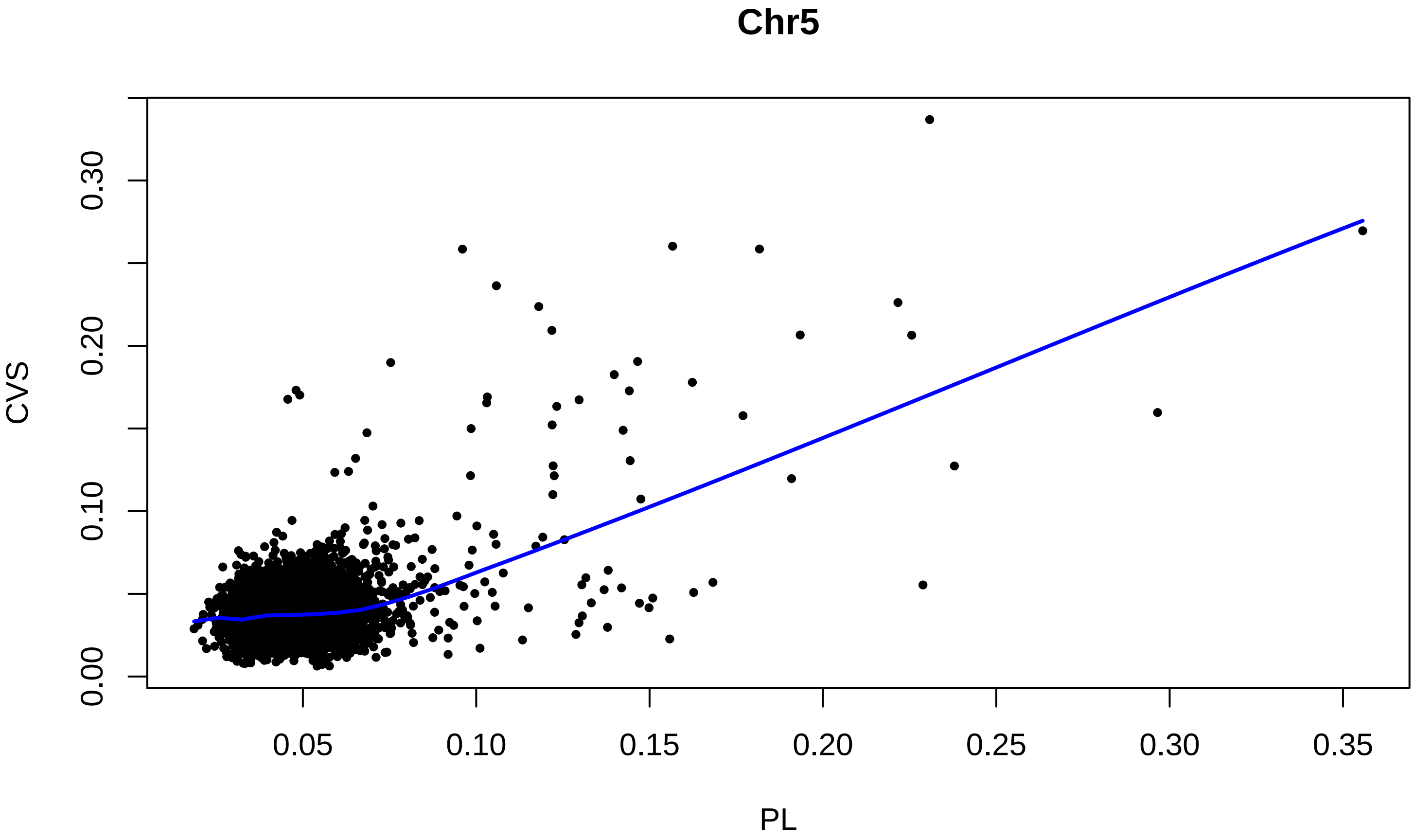

Chr5
CVS
0.00
0.10
0.20
0.30
0.05
0.10
0.15
0.20
0.25
0.30
0.35
PL

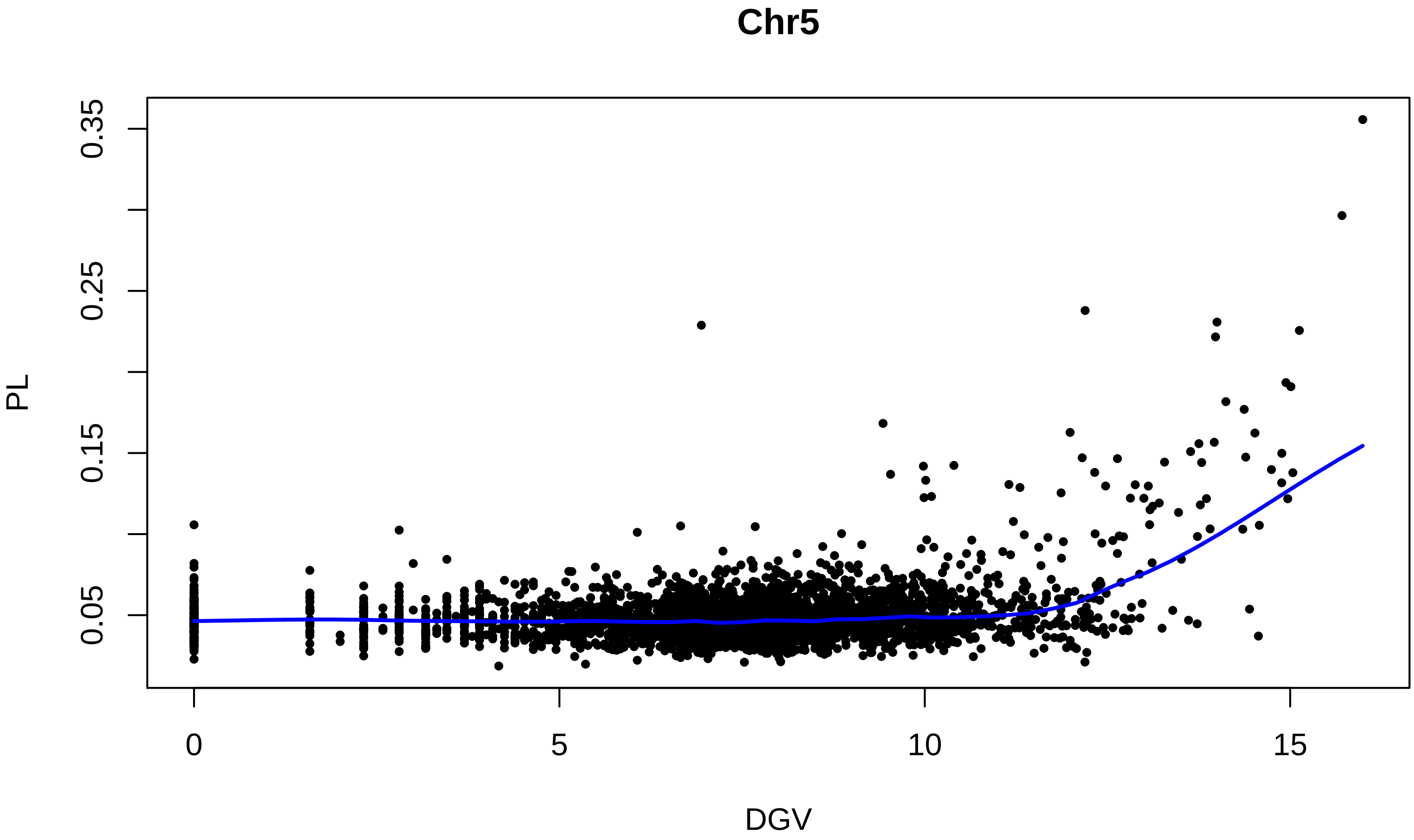
Chr5
PL
DGV
0.05
0.15
0.25
0.35
0
5
10
15

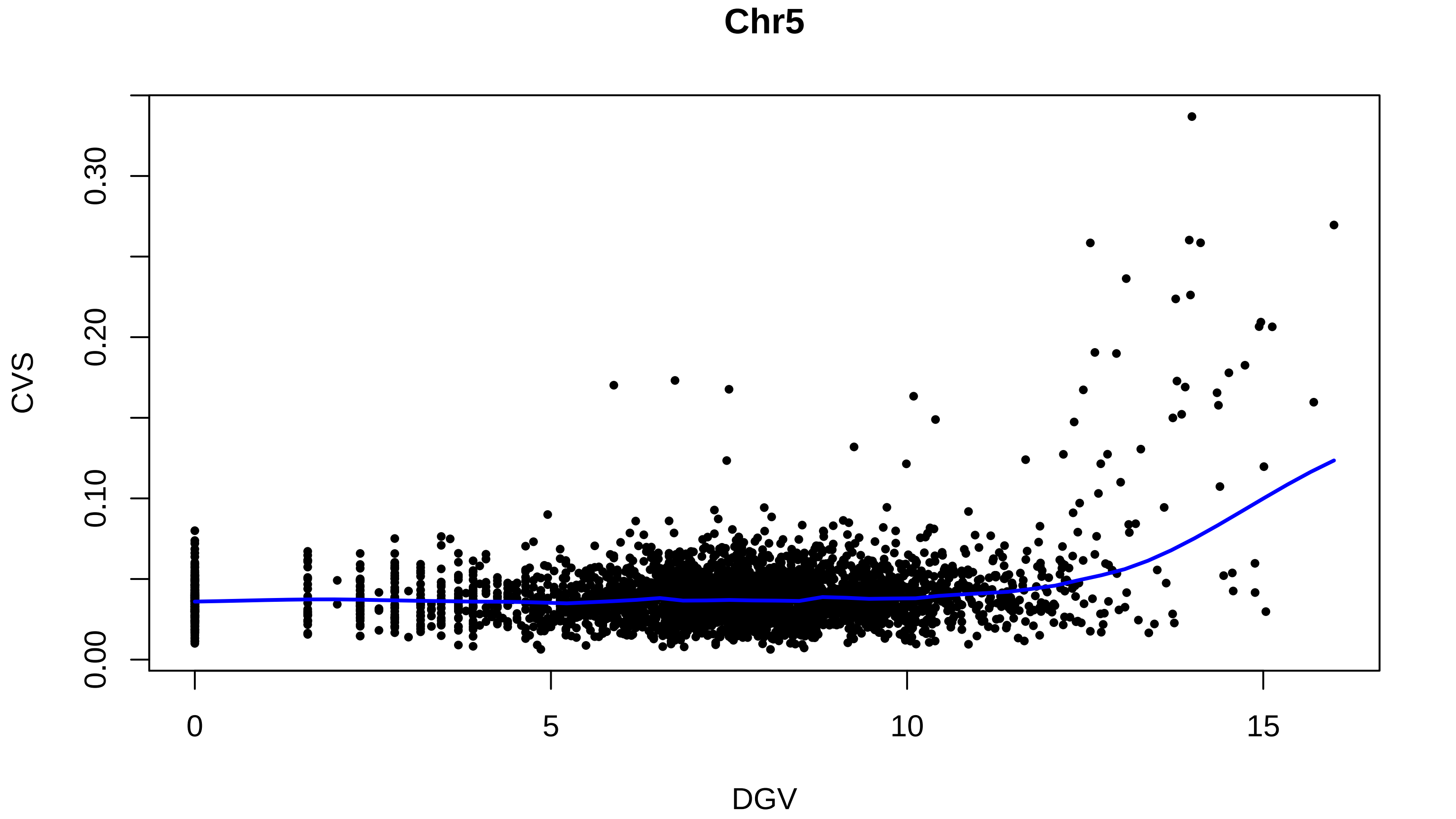

Chr5
CVS
0.00
0.10
0.20
0.30
DGV
0
5
10
15

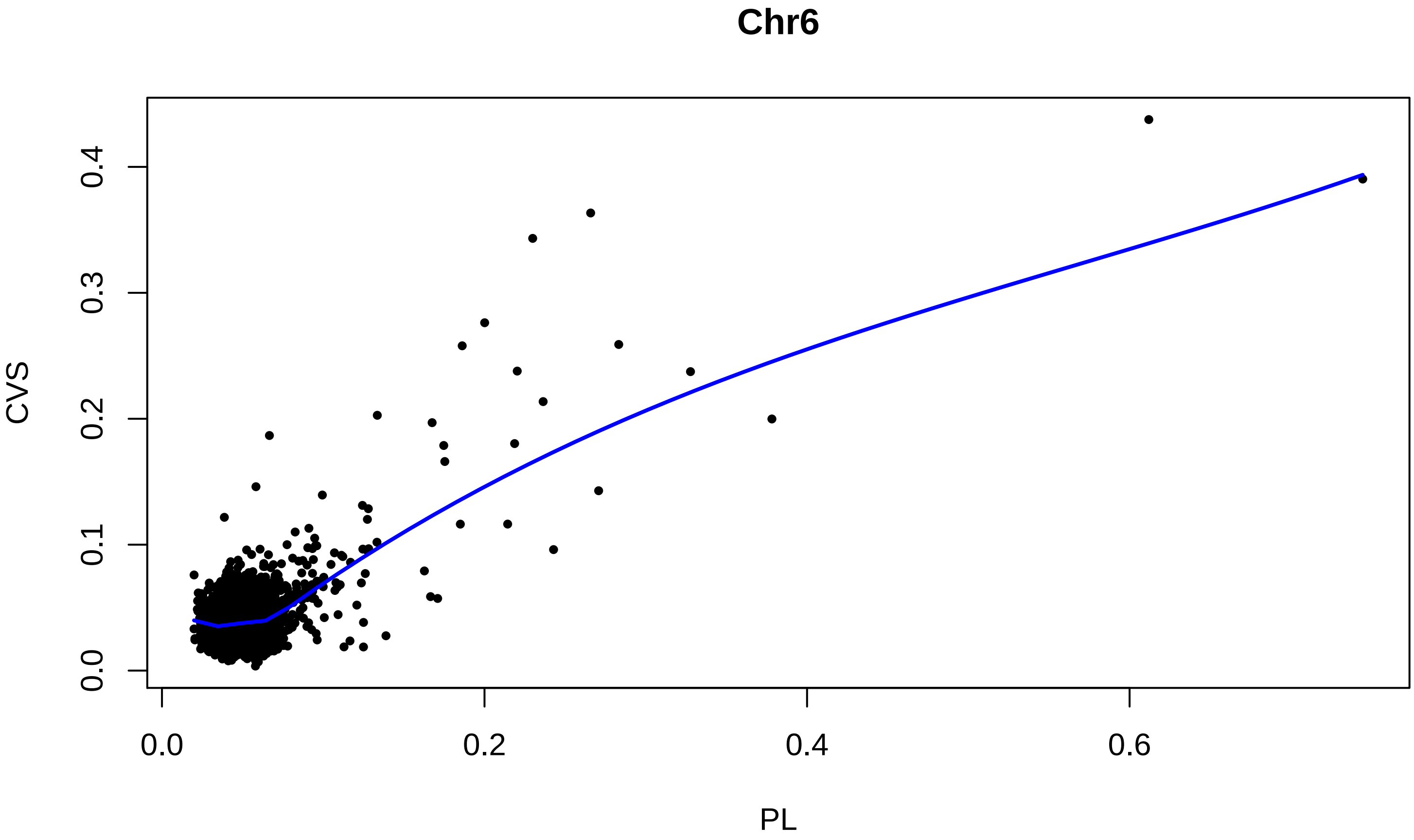
Chr6
CVS
PL
0.0
0.1
0.2
0.3
0.4
0.0
0.2
0.4
0.6

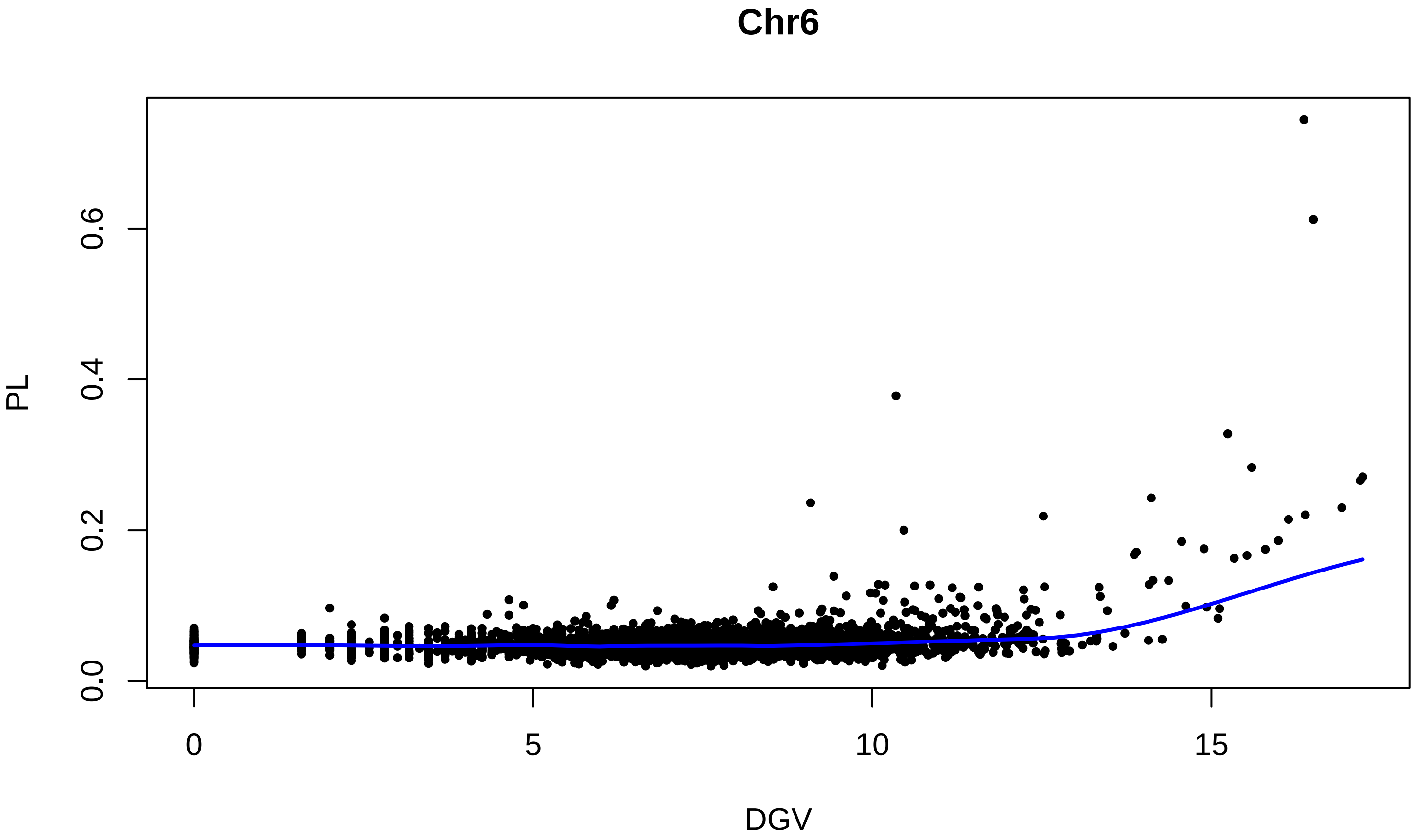

Chr6
PL
DGV
0.0
0.2
0.4
0.6
0
5
10
15

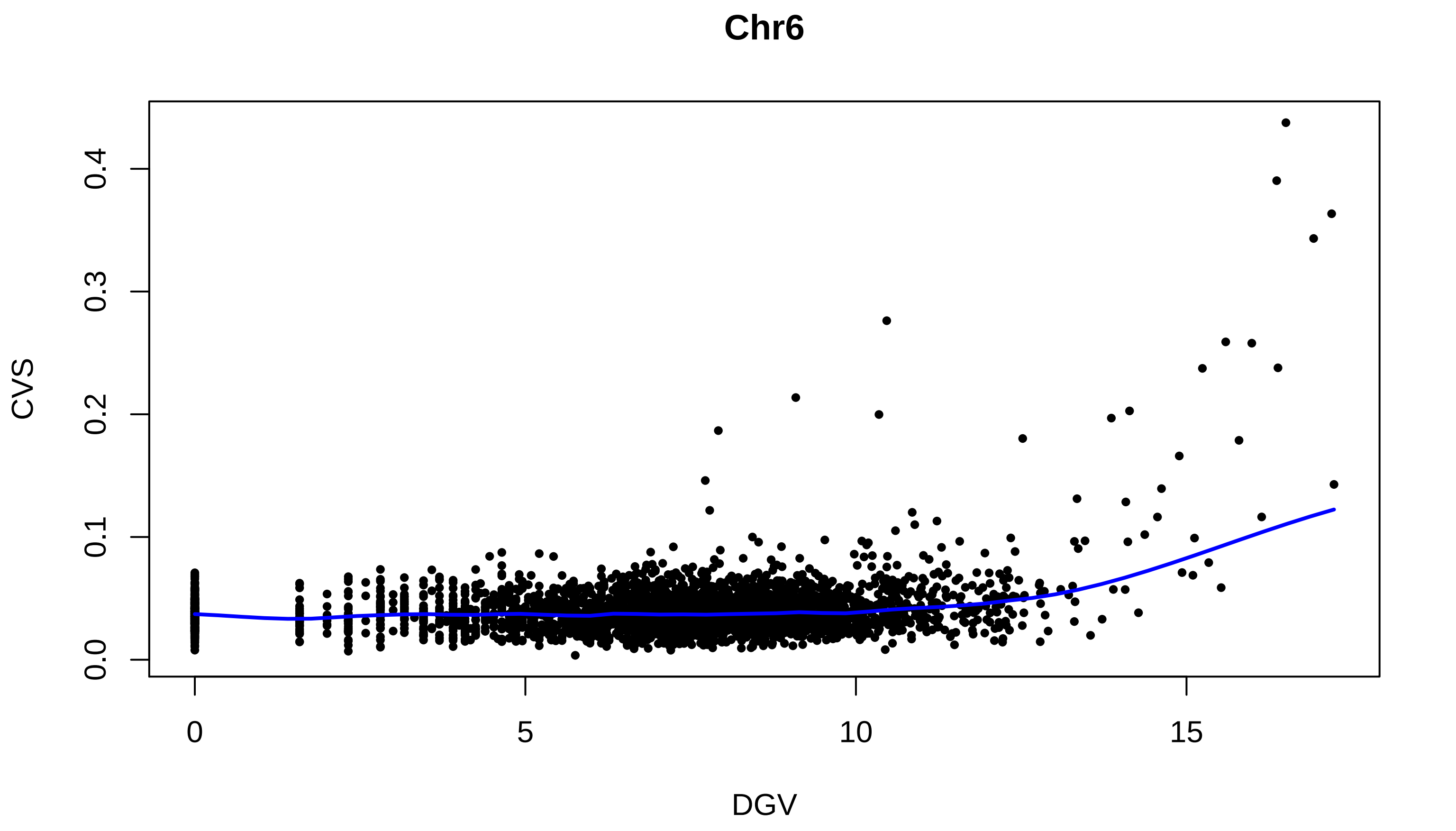

Chr6
CVS
0.0
0.1
0.2
0.3
0.4
DGV
0
5
10
15

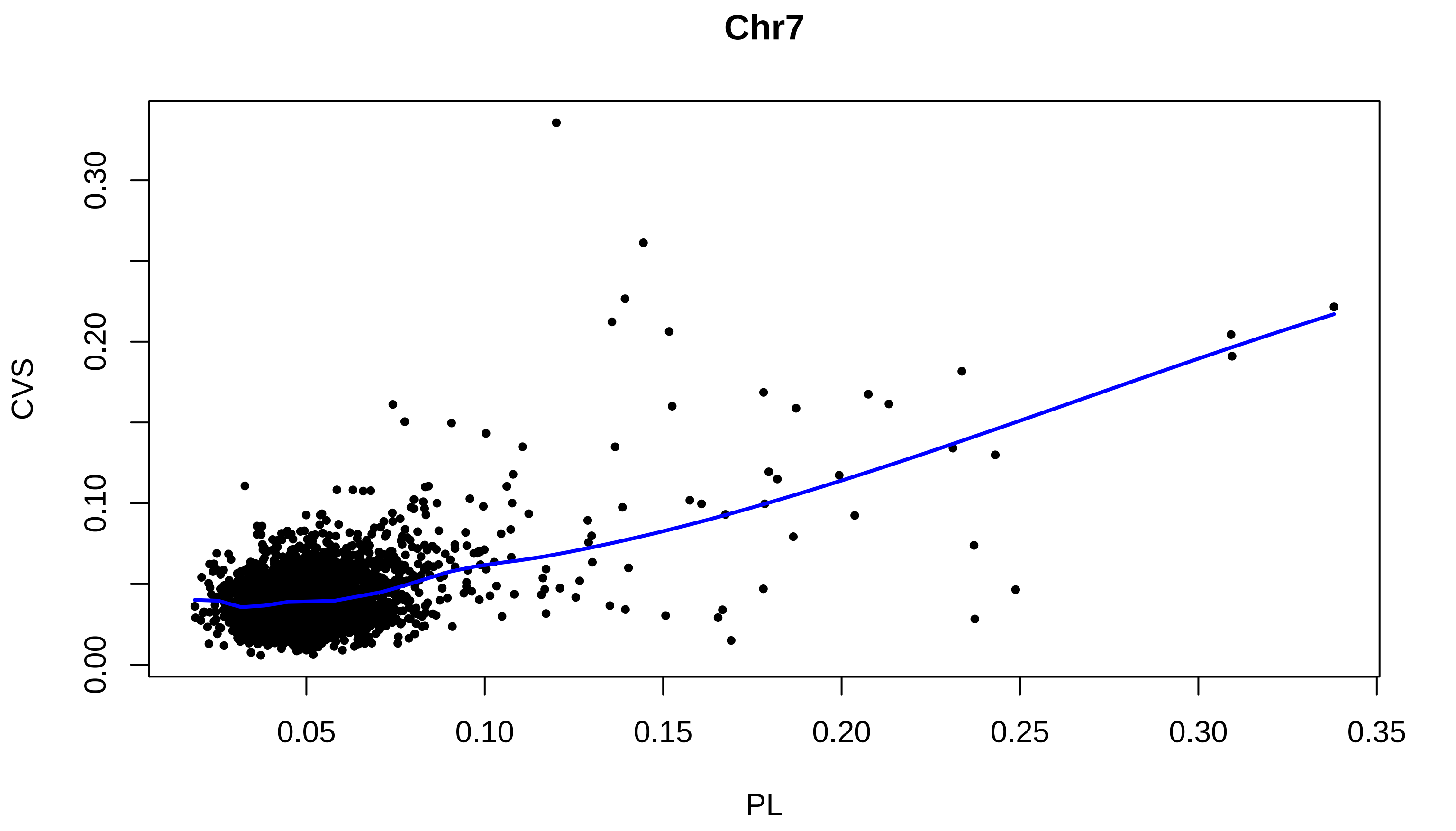

Chr7
CVS
0.00
0.10
0.20
0.30
0.05
0.10
0.15
0.20
0.25
0.30
0.35
PL

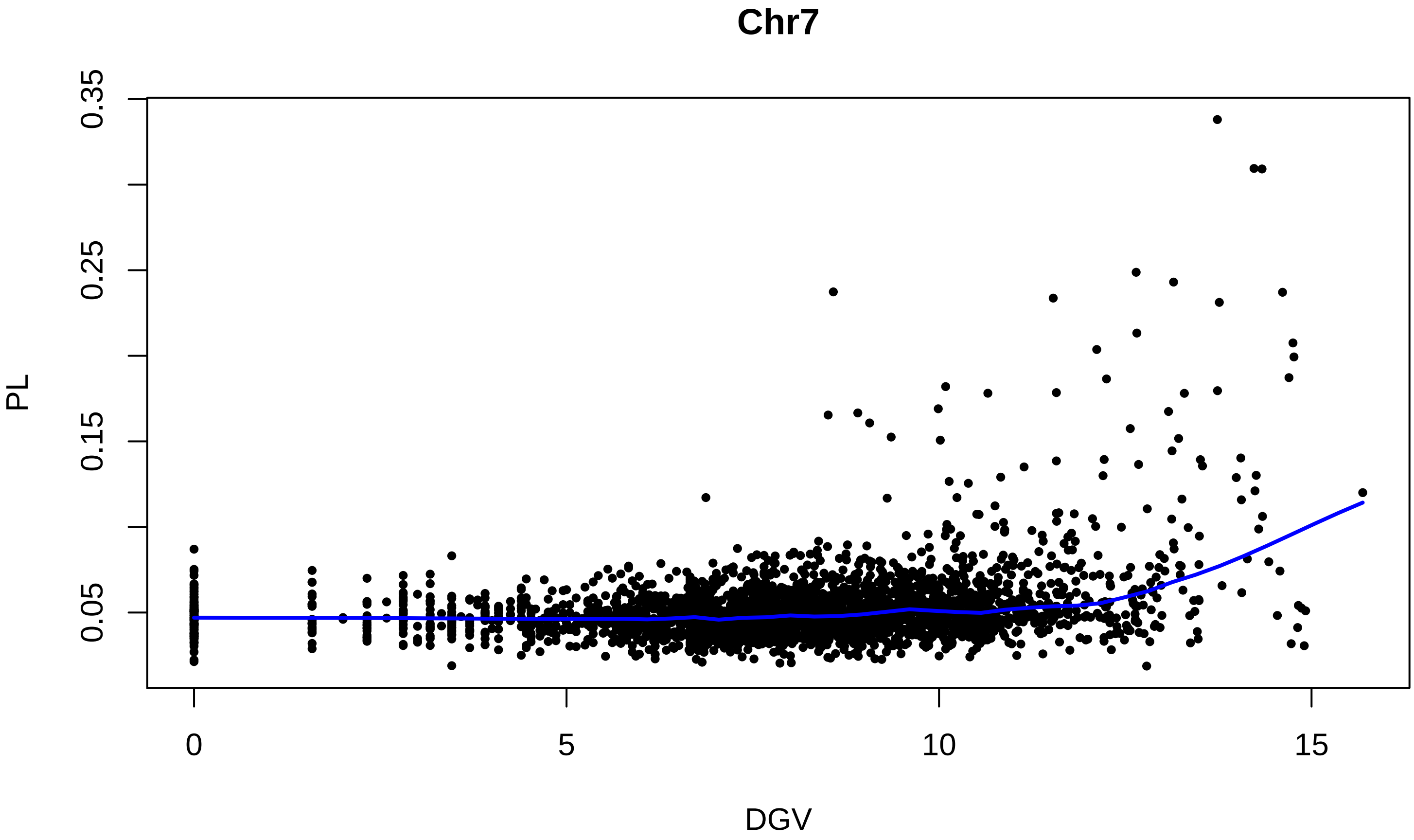
Chr7
PL
0.05
0.15
0.25
0.35
0
5
10
15
DGV

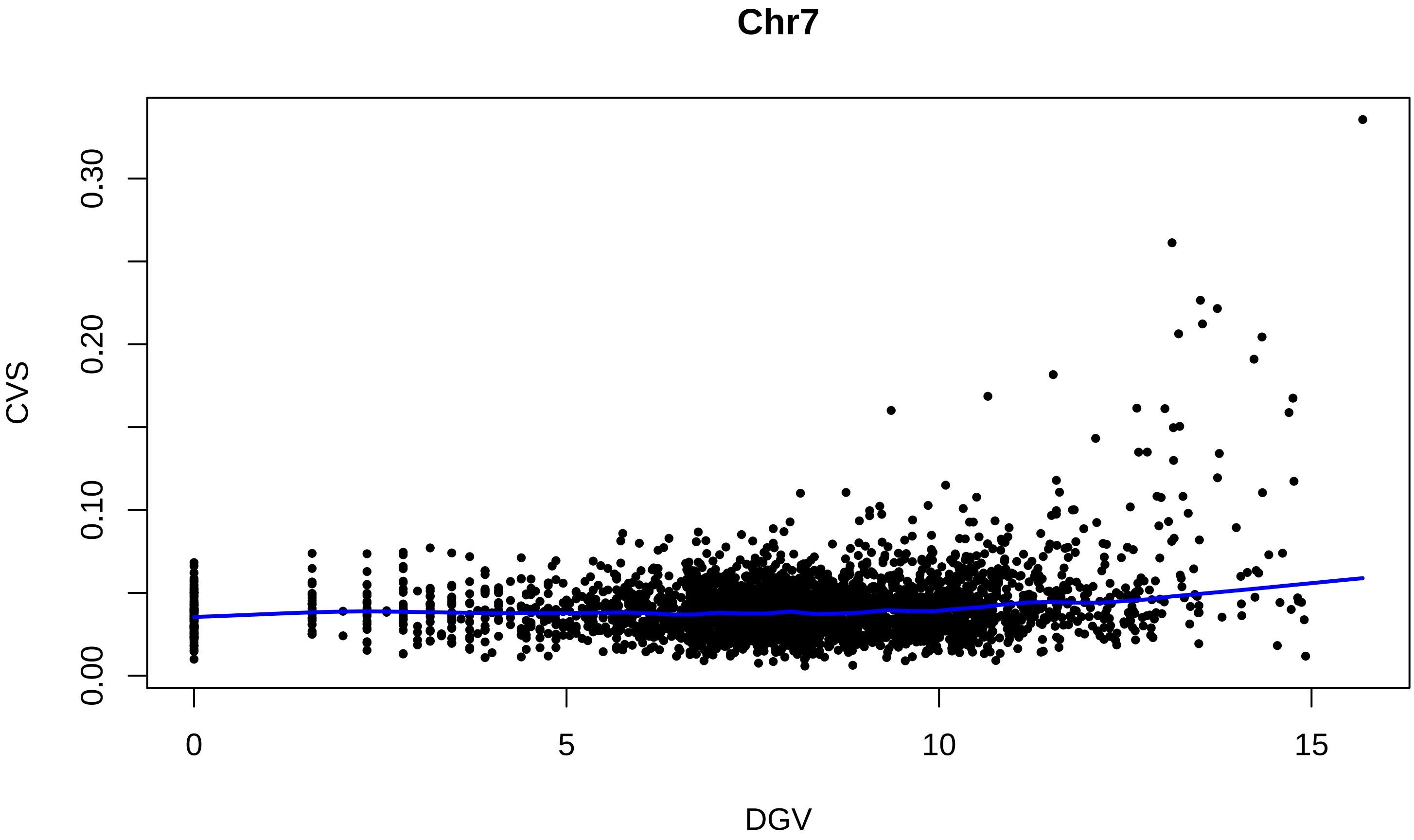
Chr7
CVS
DGV
0.00
0.10
0.20
0.30
0
5
10
15

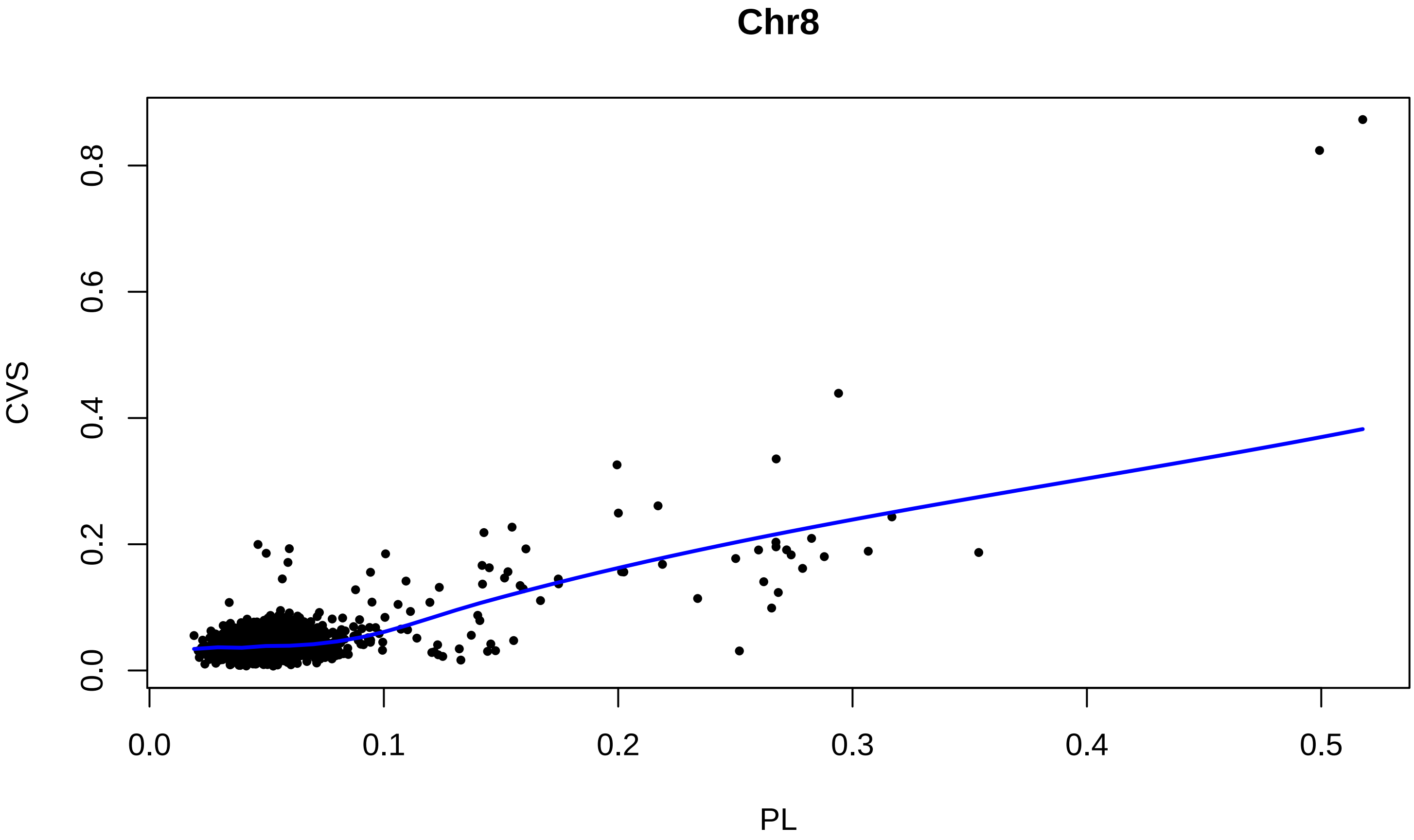
Chr8
CVS
0.0
0.2
0.4
0.6
0.8
PL
0.0
0.1
0.2
0.3
0.4
0.5

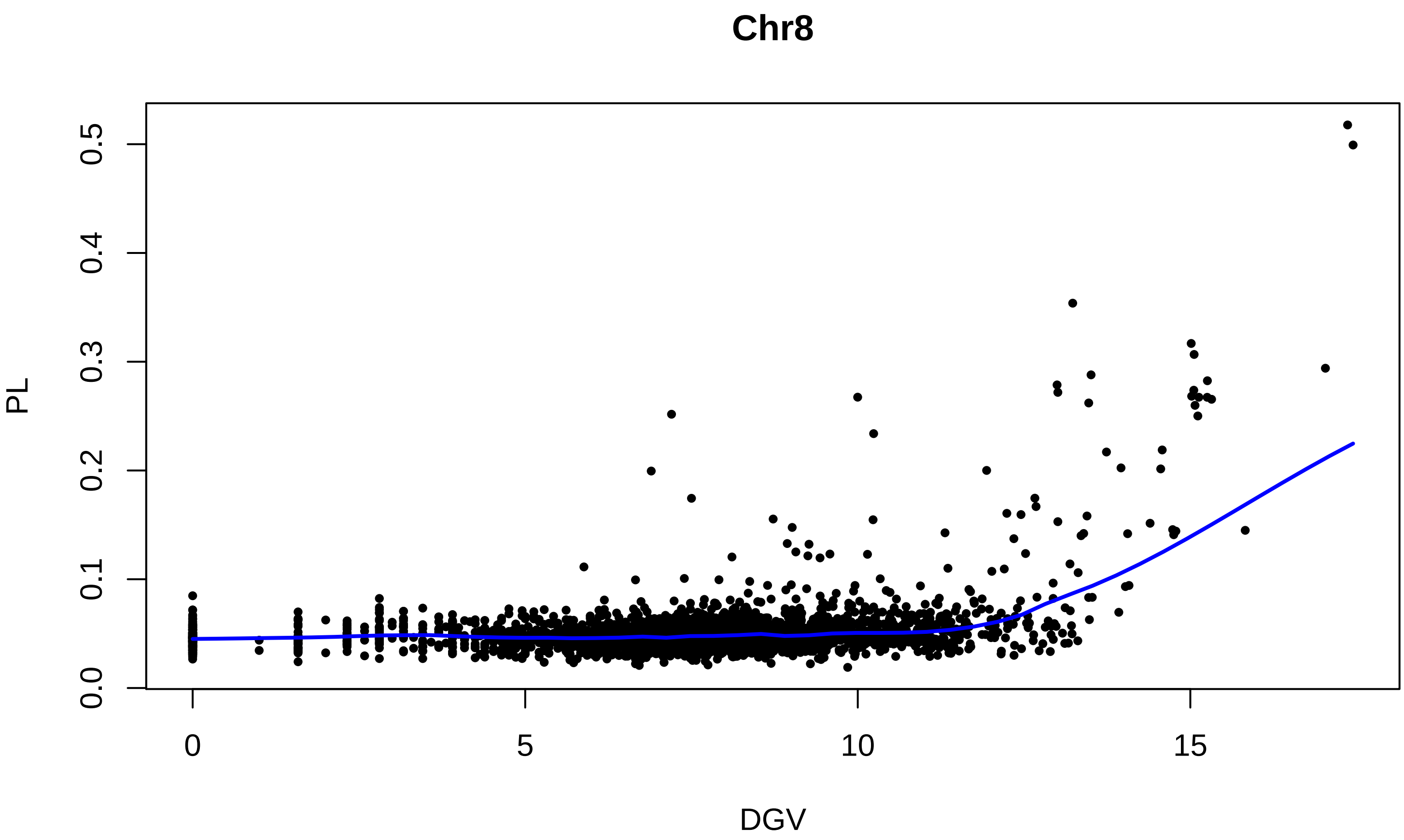

Chr8
PL
0.0
0.1
0.2
0.3
0.4
0.5
0
5
10
15
DGV

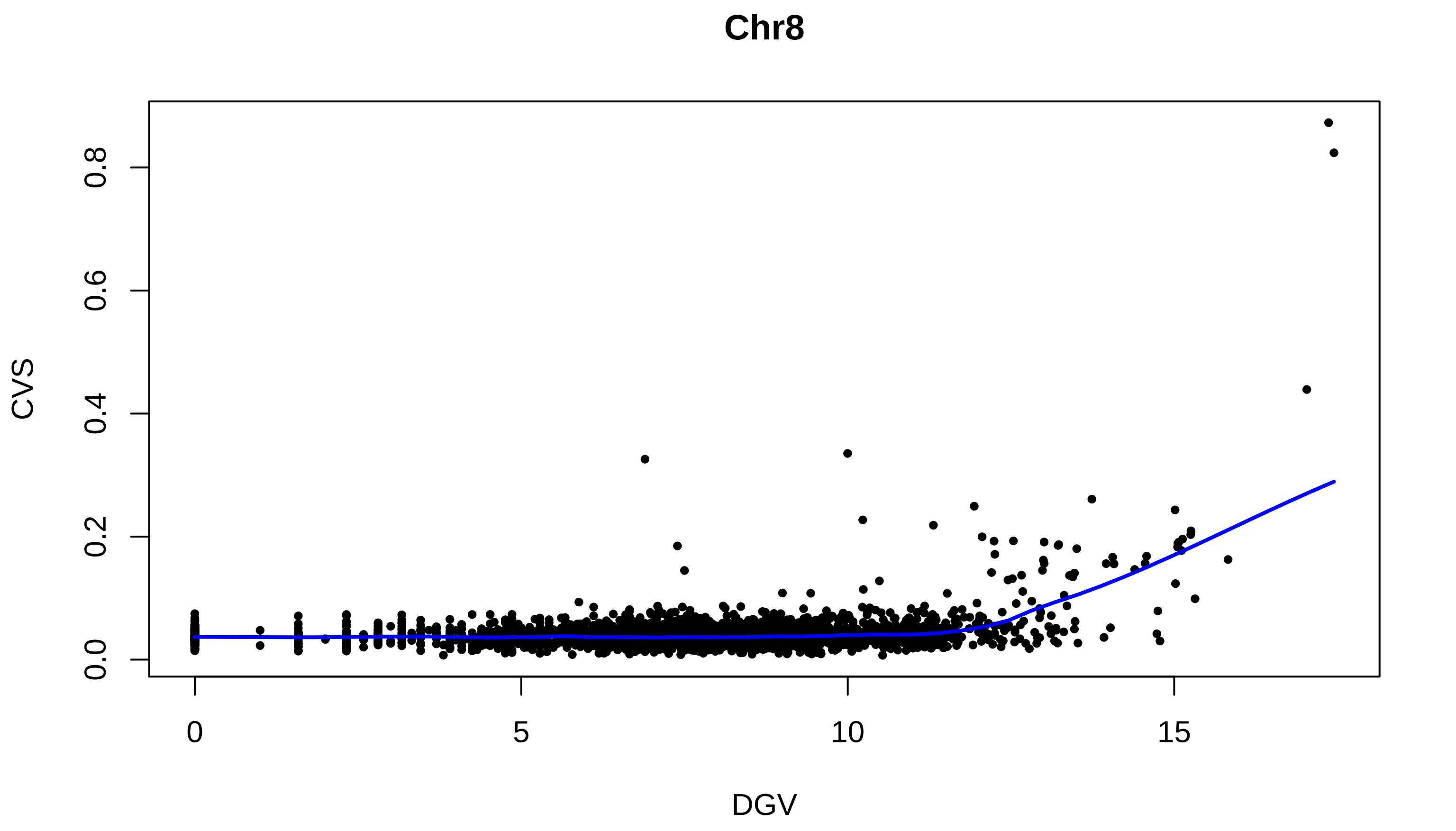
Chr8
CVS
0.0
0.2
0.4
0.6
0.8
0
5
10
15
DGV

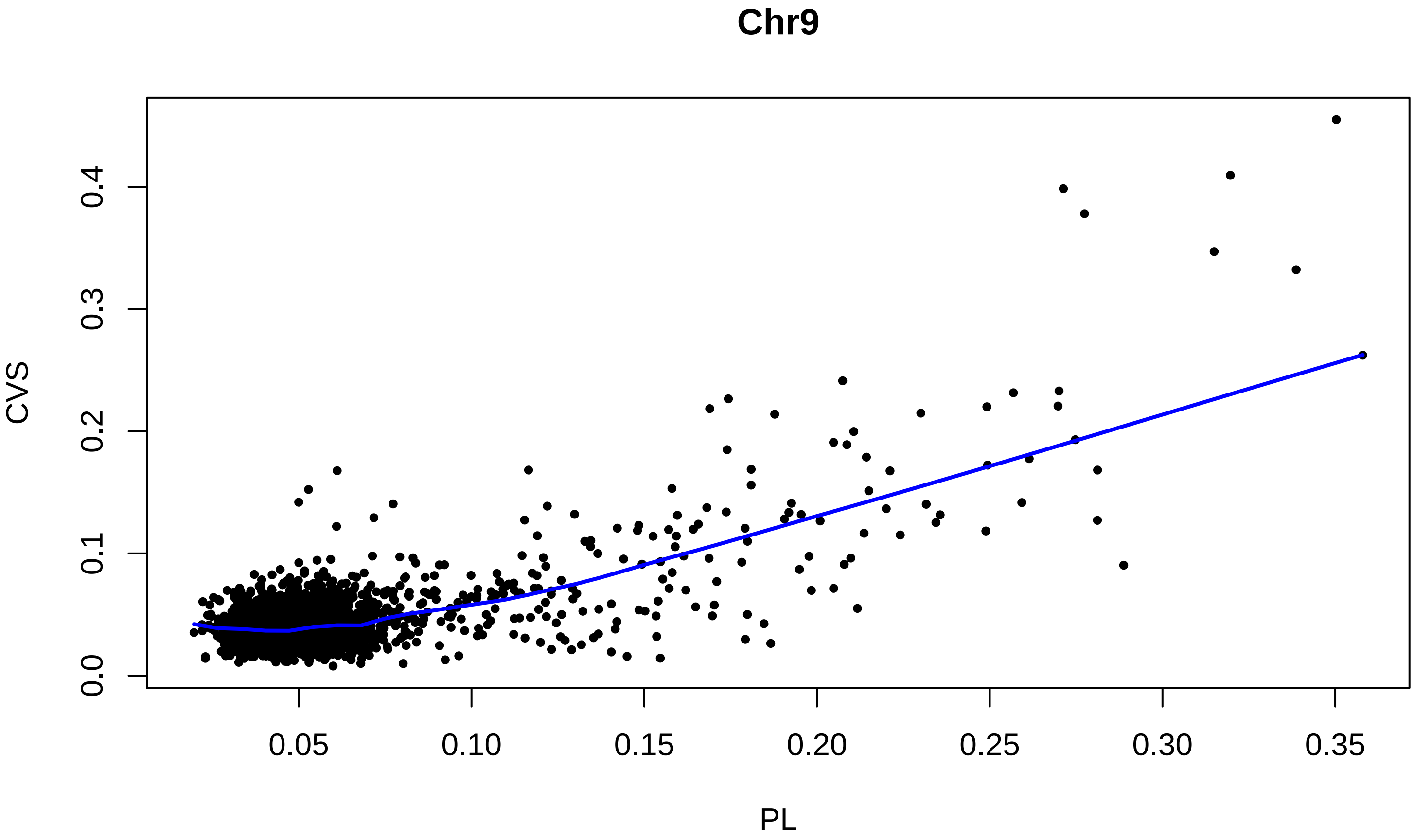

Chr9
CVS
PL
0.0
0.1
0.2
0.3
0.4
0.05
0.10
0.15
0.20
0.25
0.30
0.35

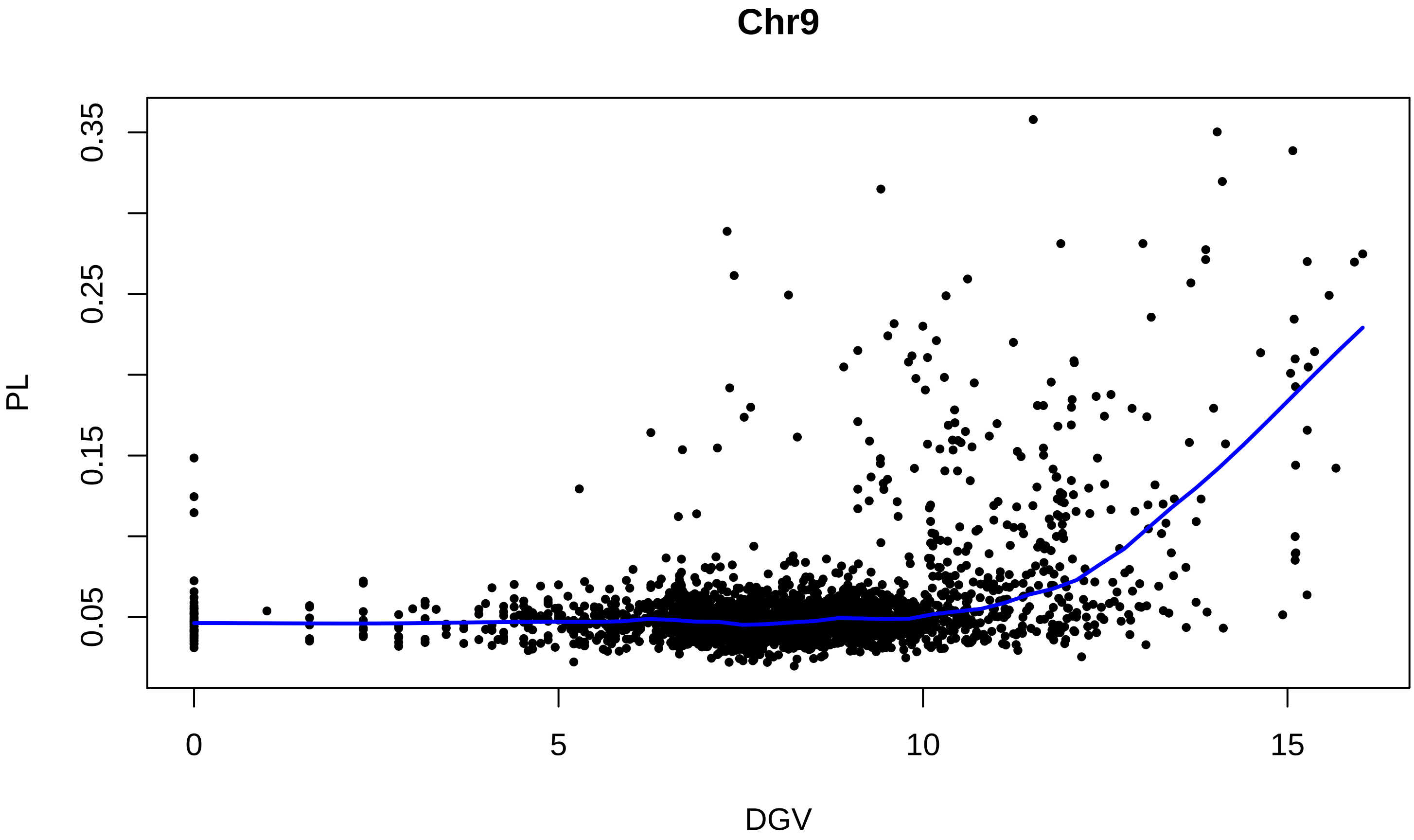

Chr9
PL
0.05
0.15
0.25
0.35
0
5
10
15
DGV

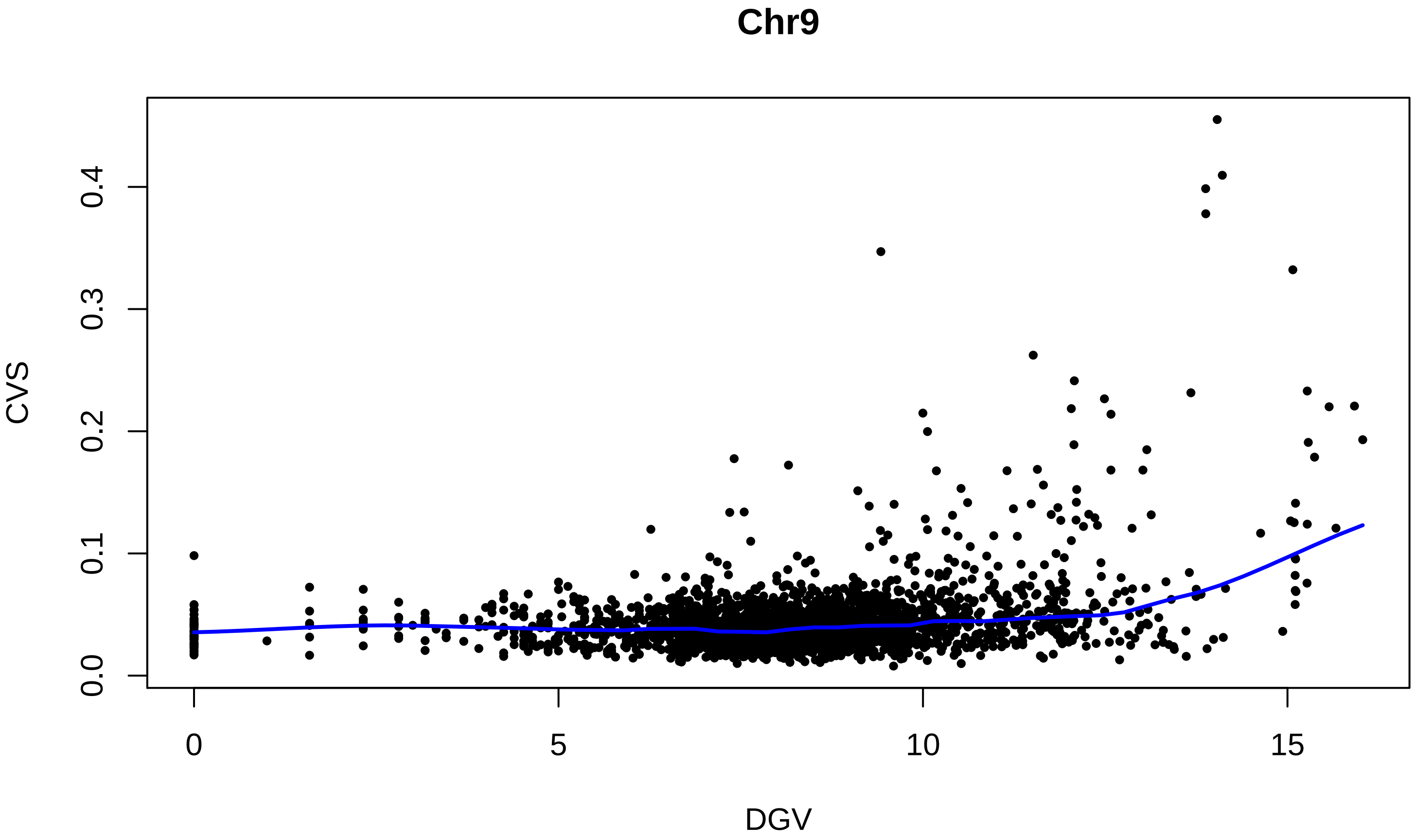

Chr9
CVS
DGV
0.0
0.1
0.2
0.3
0.4
0
5
10
15

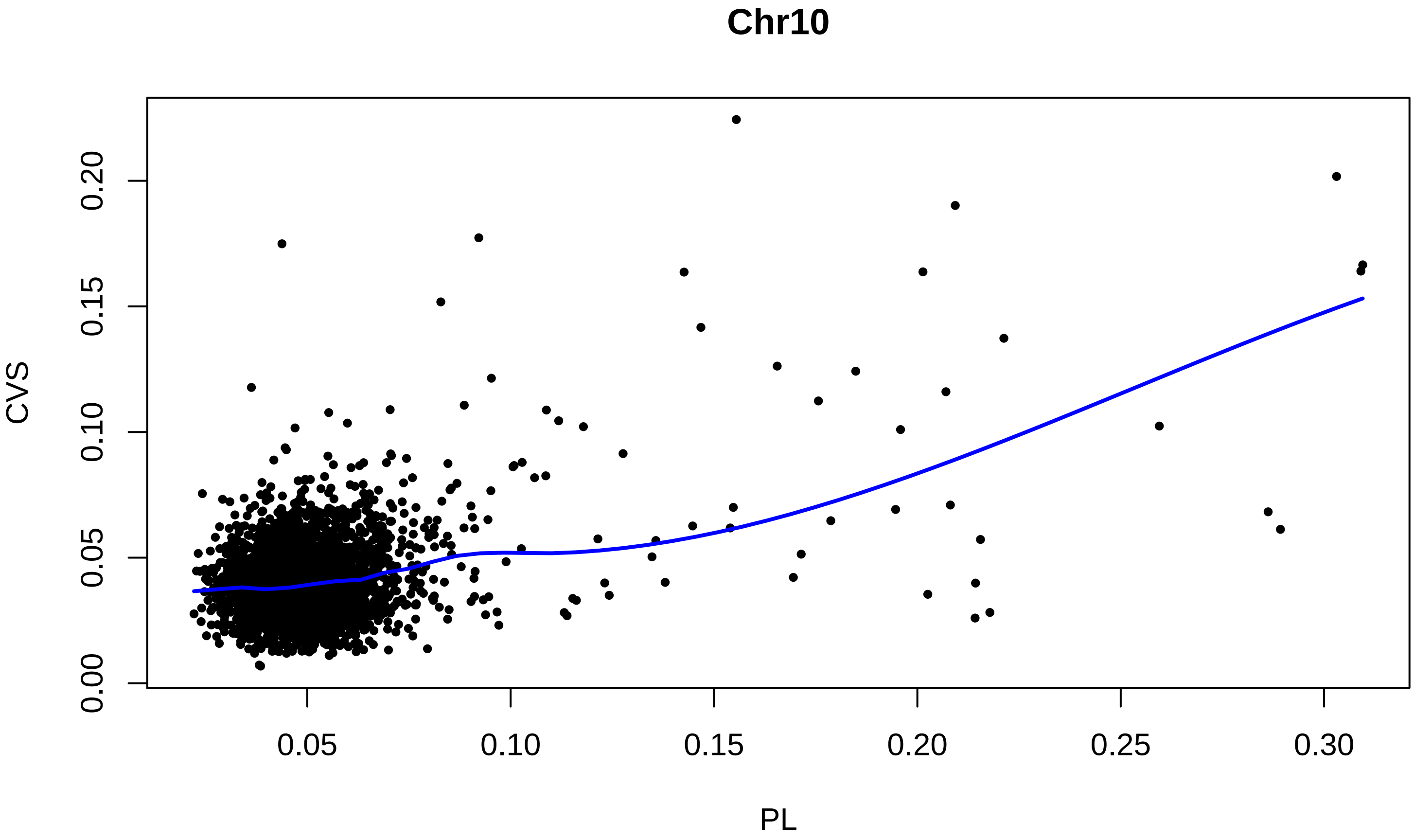
Chr10
CVS
PL
0.00
0.05
0.10
0.15
0.20
0.05
0.10
0.15
0.20
0.25
0.30

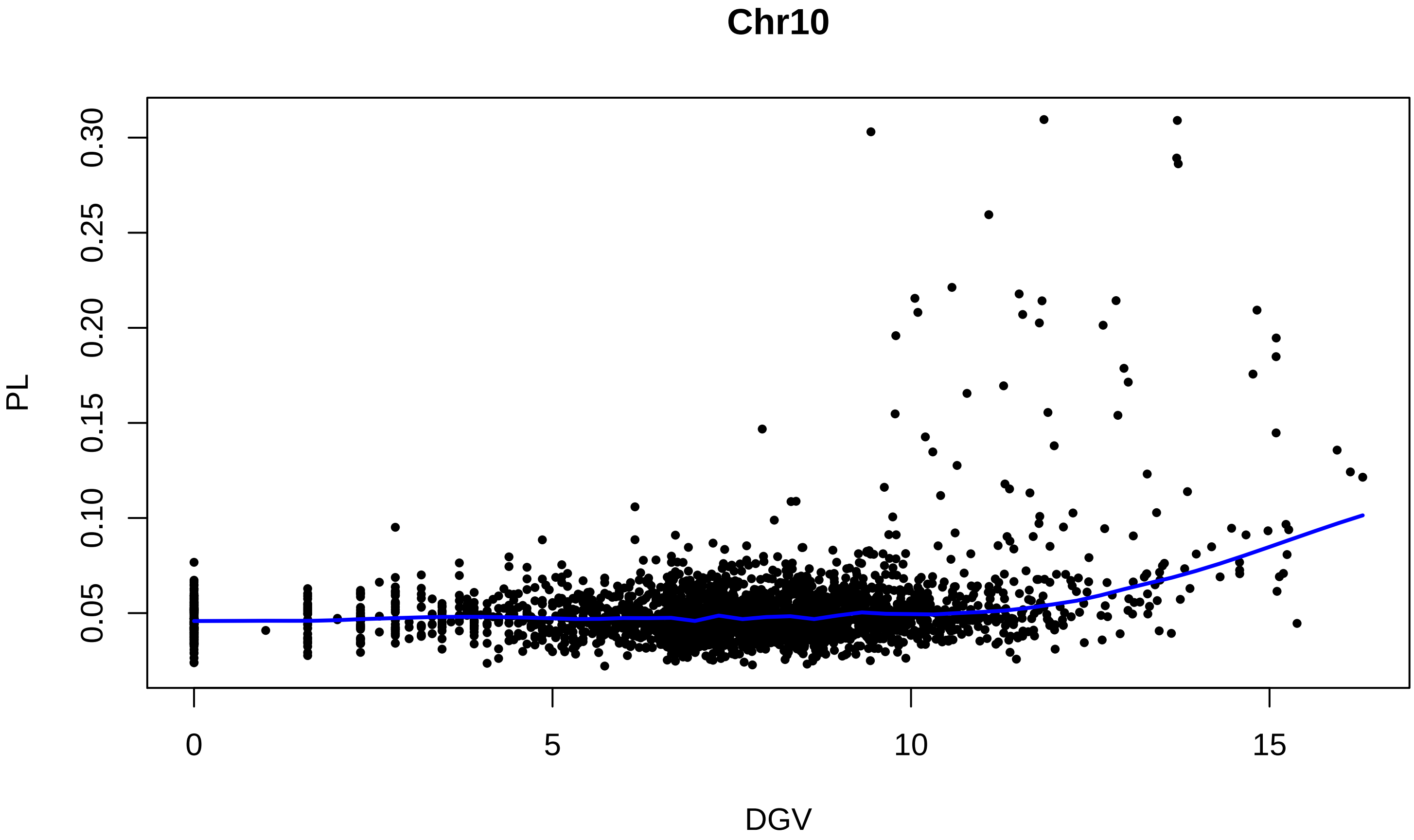

Chr10
PL
DGV
0.05
0.10
0.15
0.20
0.25
0.30
0
5
10
15

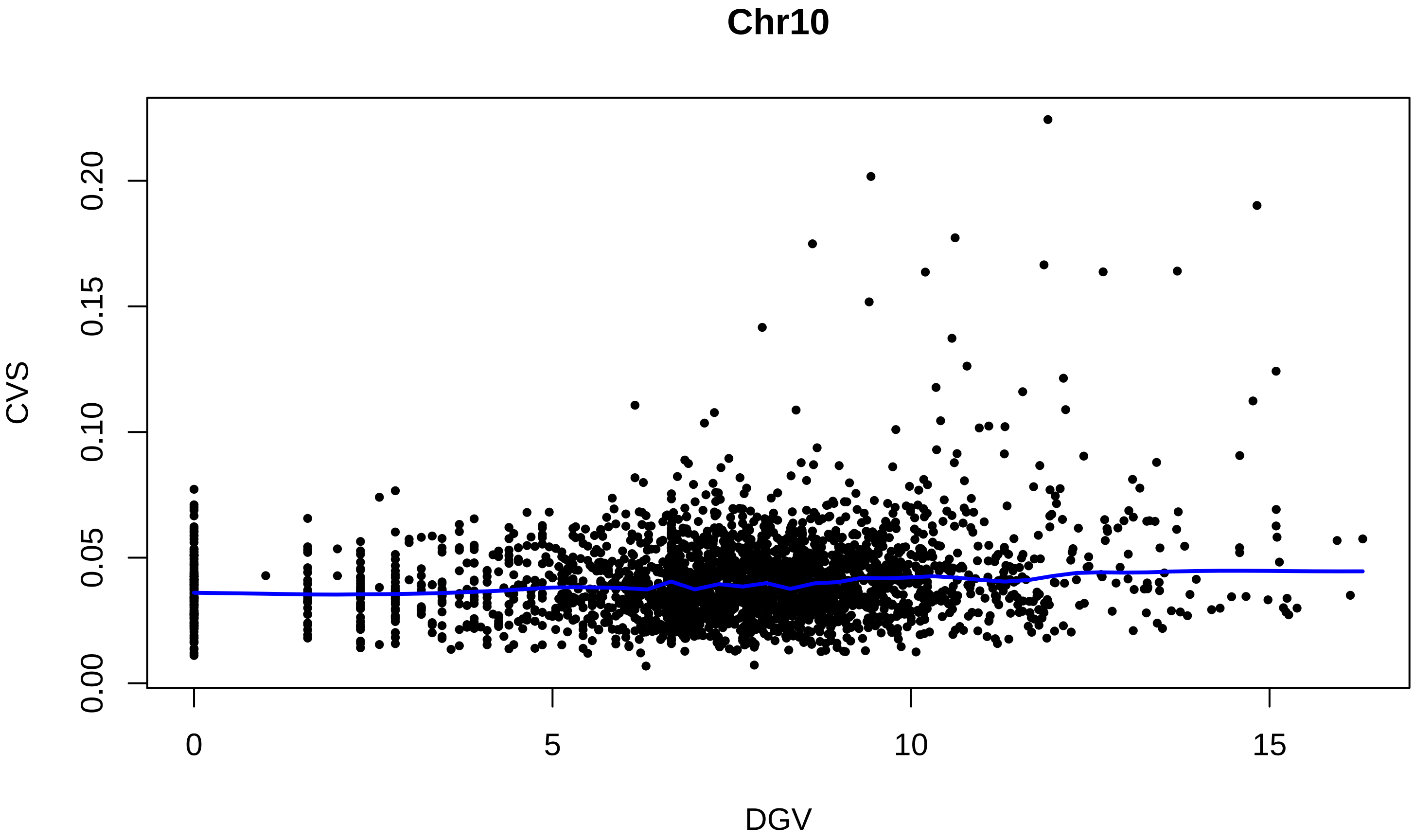
Chr10
CVS
DGV
0.00
0.05
0.10
0.15
0.20
0
5
10
15

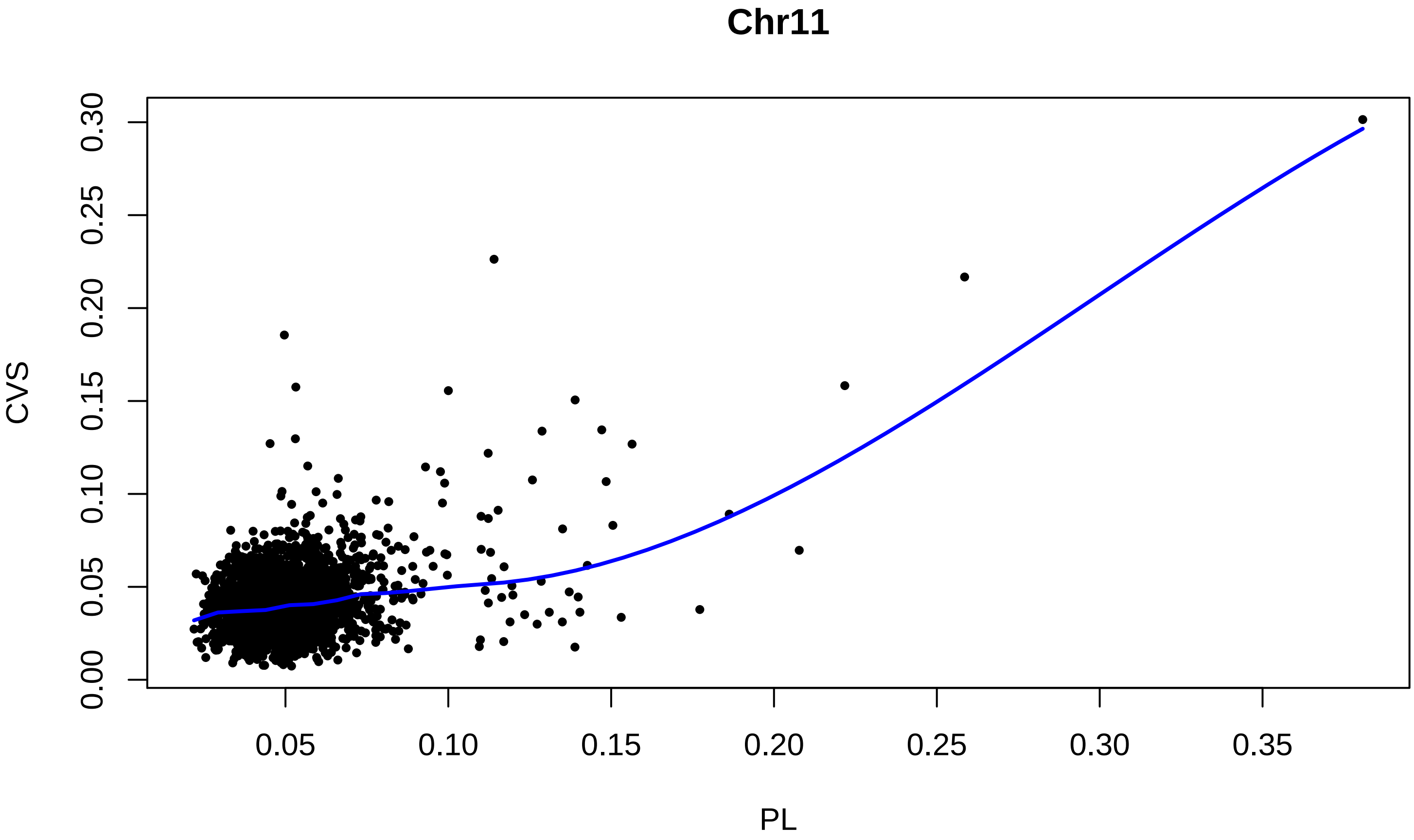

Chr11
CVS
0.00
0.05
0.10
0.15
0.20
0.25
0.30
0.05
0.10
0.15
0.20
0.25
0.30
0.35
PL

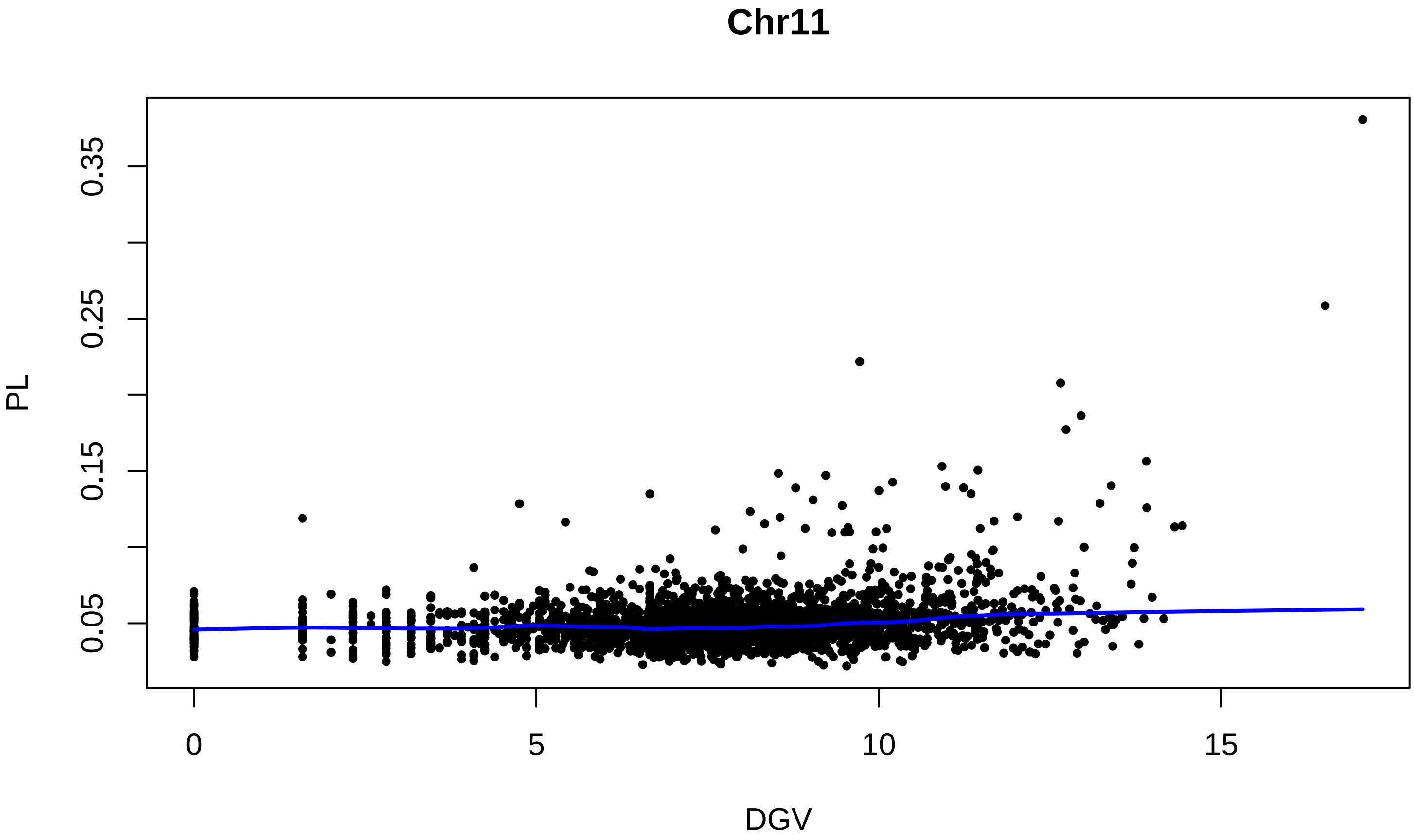

Chr11
PL
0.05
0.15
0.25
0.35
0
5
10
15
DGV

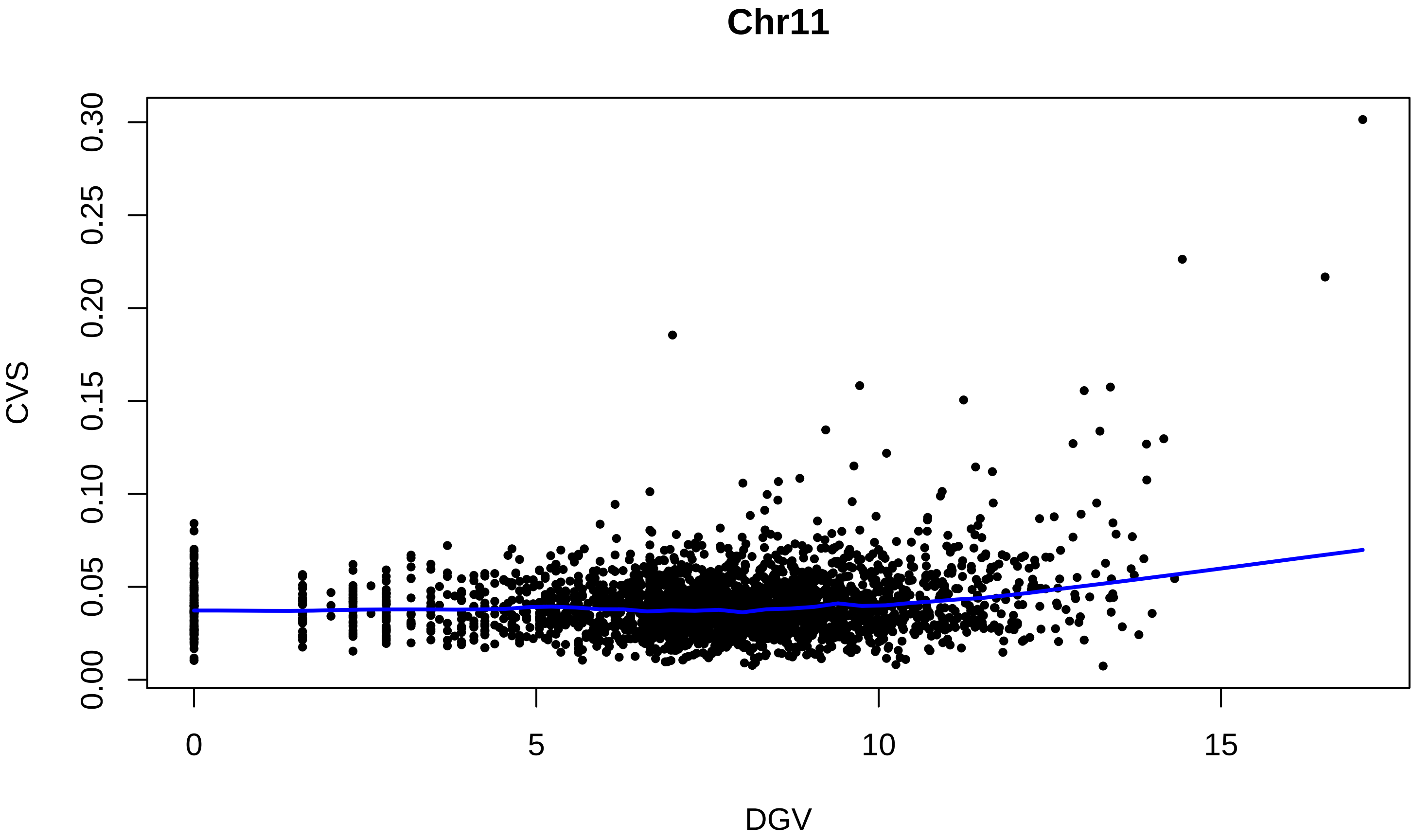
Chr11
CVS
DGV
0.00
0.05
0.10
0.15
0.20
0.25
0.30
0
5
10
15

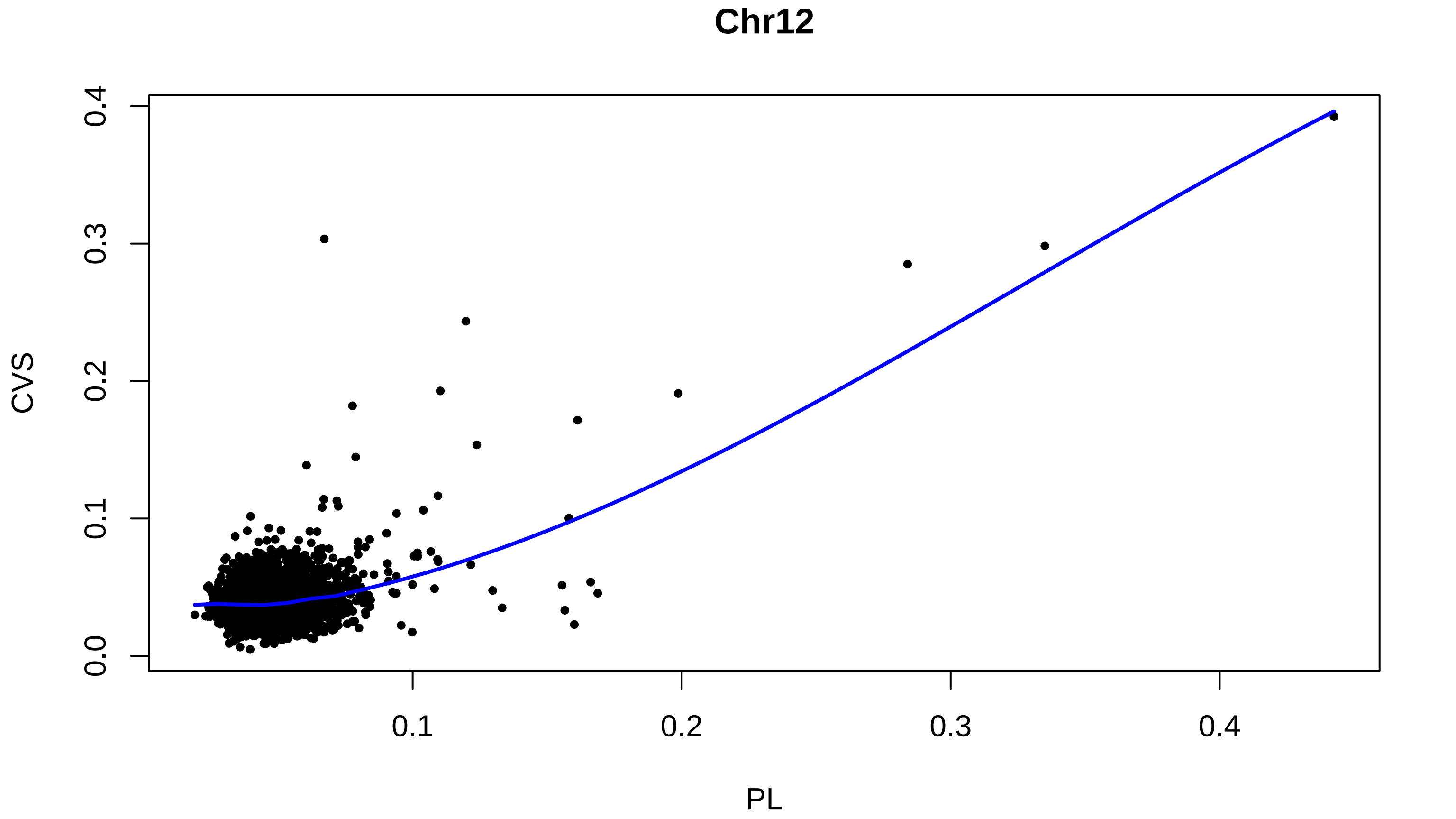

Chr12
CVS
0.0
0.1
0.2
0.3
0.4
PL
0.1
0.2
0.3
0.4

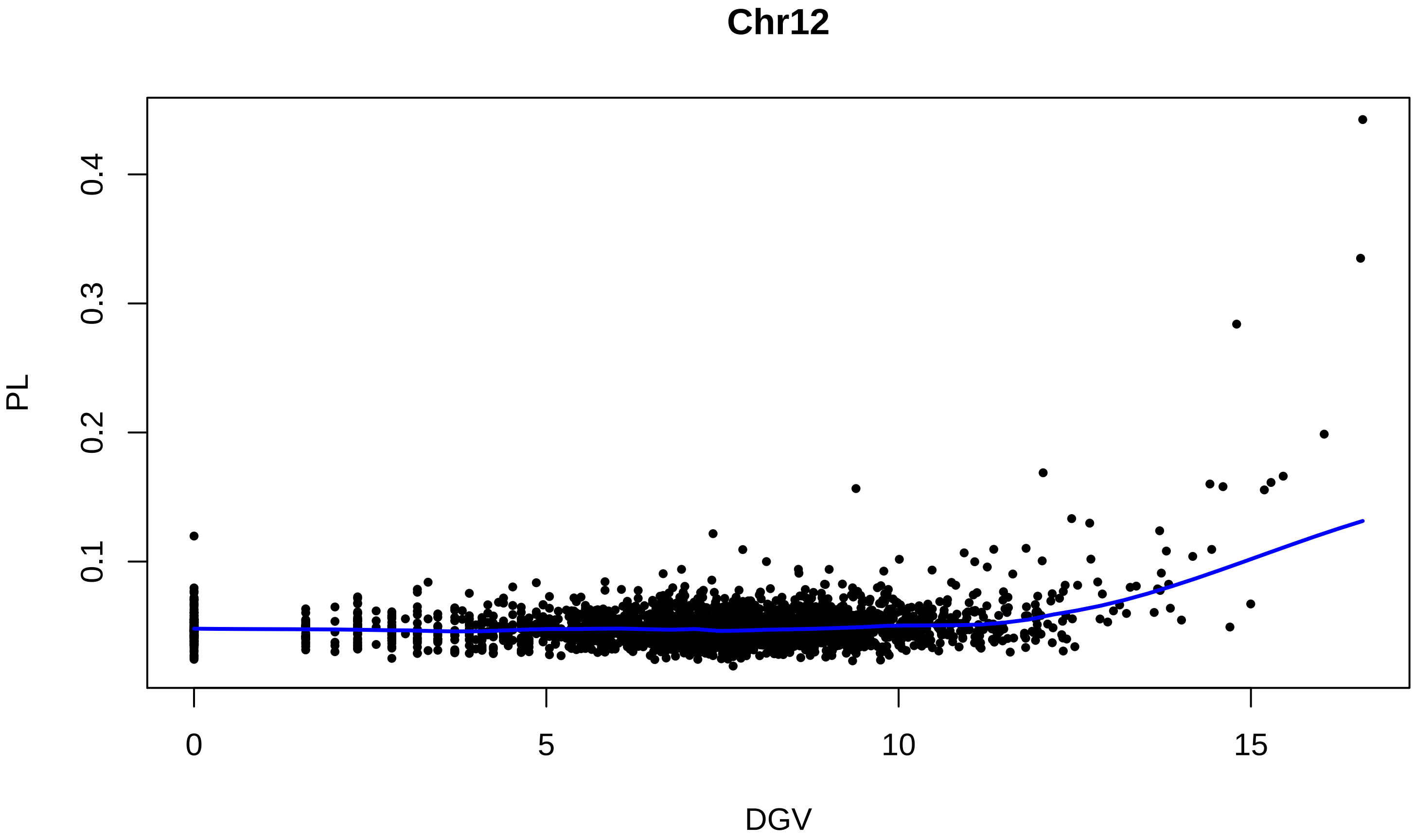
Chr12
PL
0.1
0.2
0.3
0.4
0
5
10
15
DGV

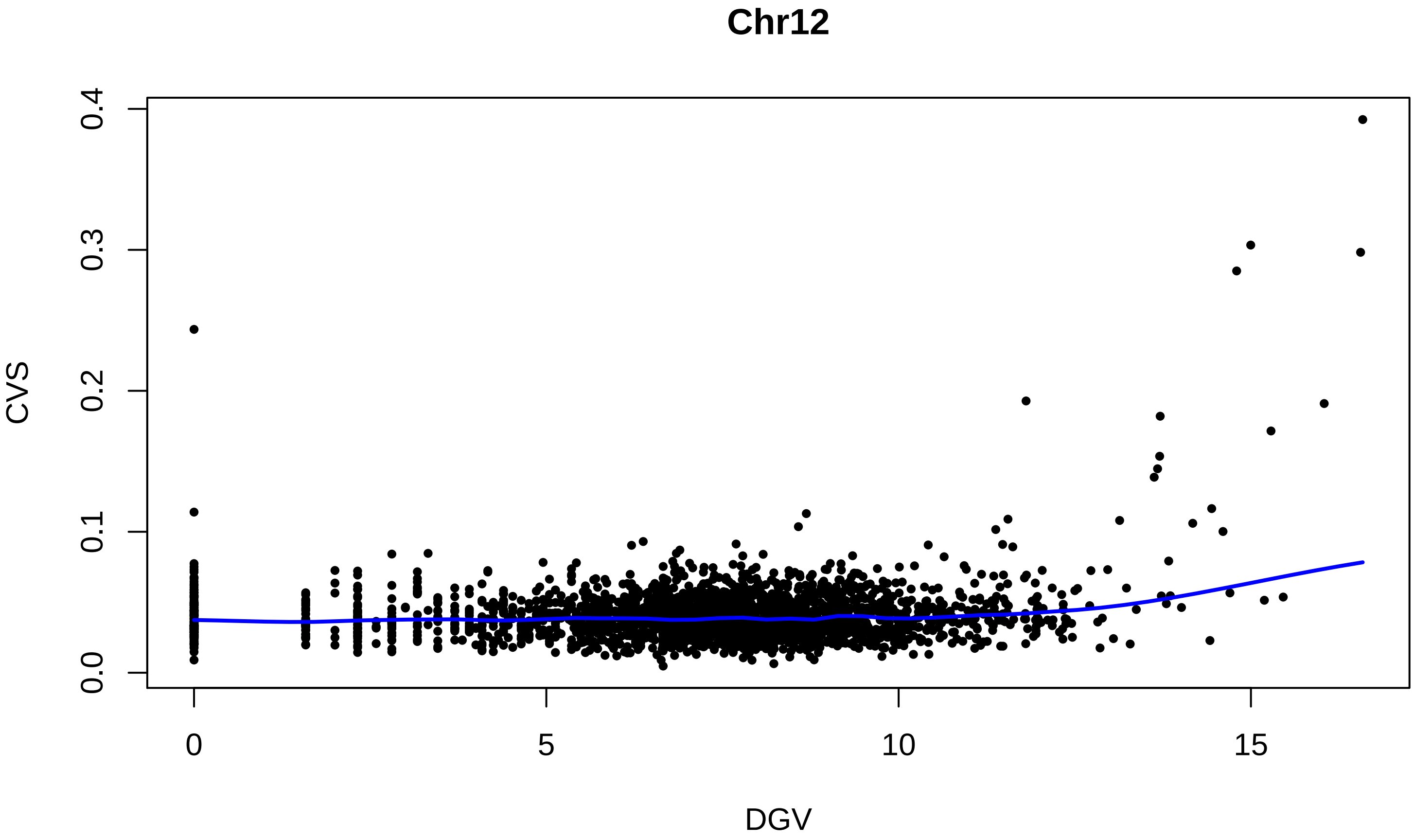

Chr12
CVS
DGV
0.0
0.1
0.2
0.3
0.4
0
5
10
15

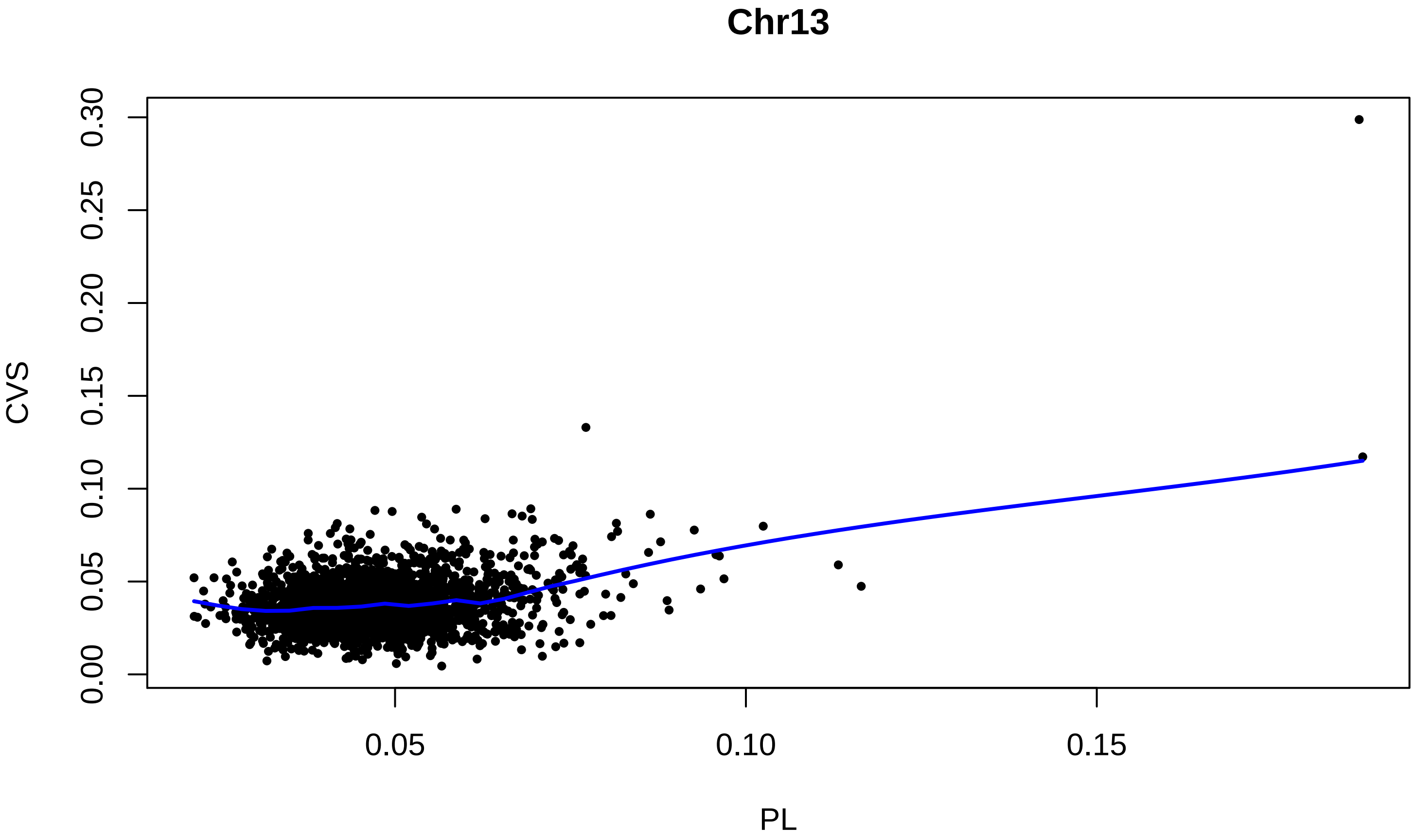
Chr13
CVS
PL
0.00
0.05
0.10
0.15
0.20
0.25
0.30
0.05
0.10
0.15

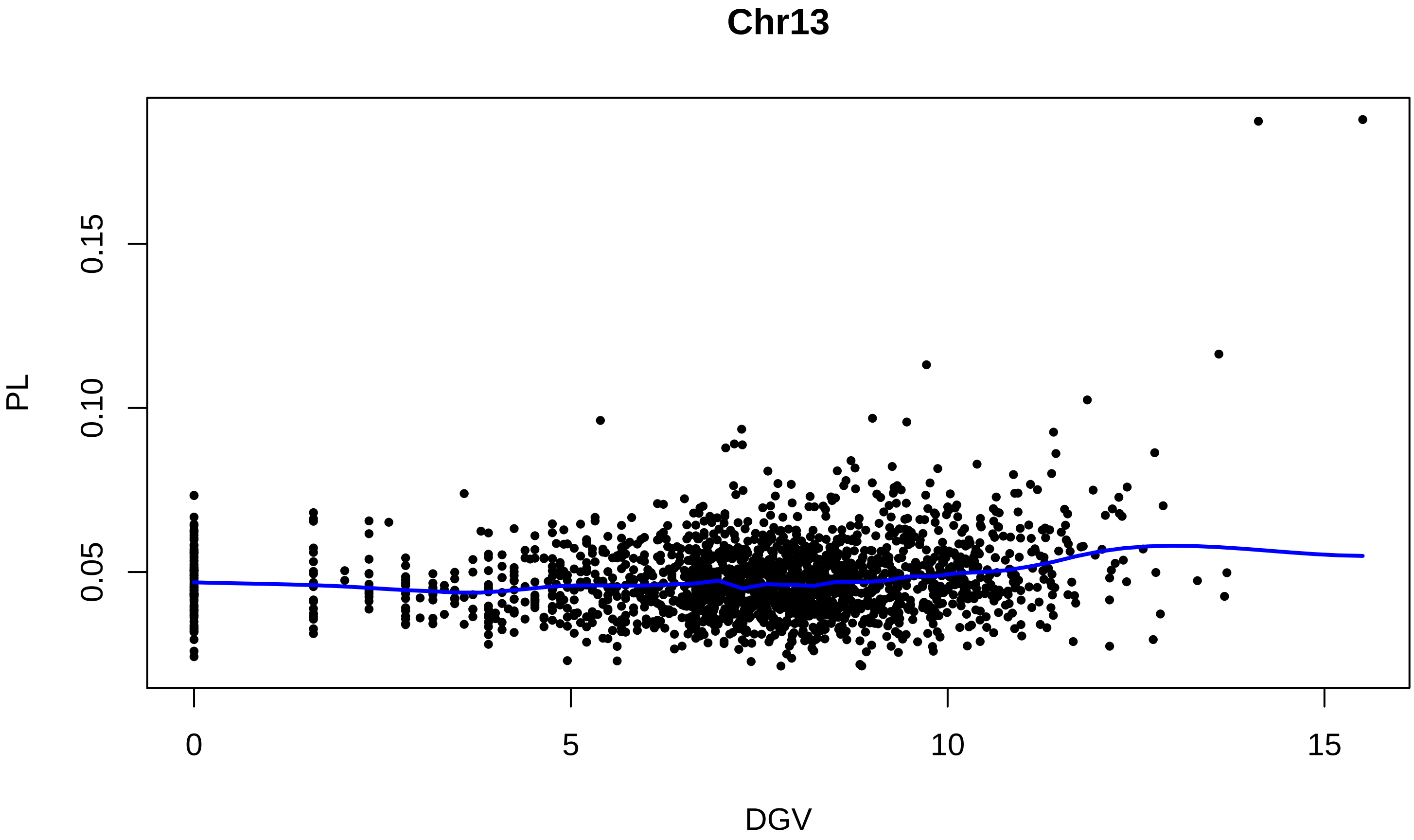

Chr13
PL
0.05
0.10
0.15
0
5
10
15
DGV

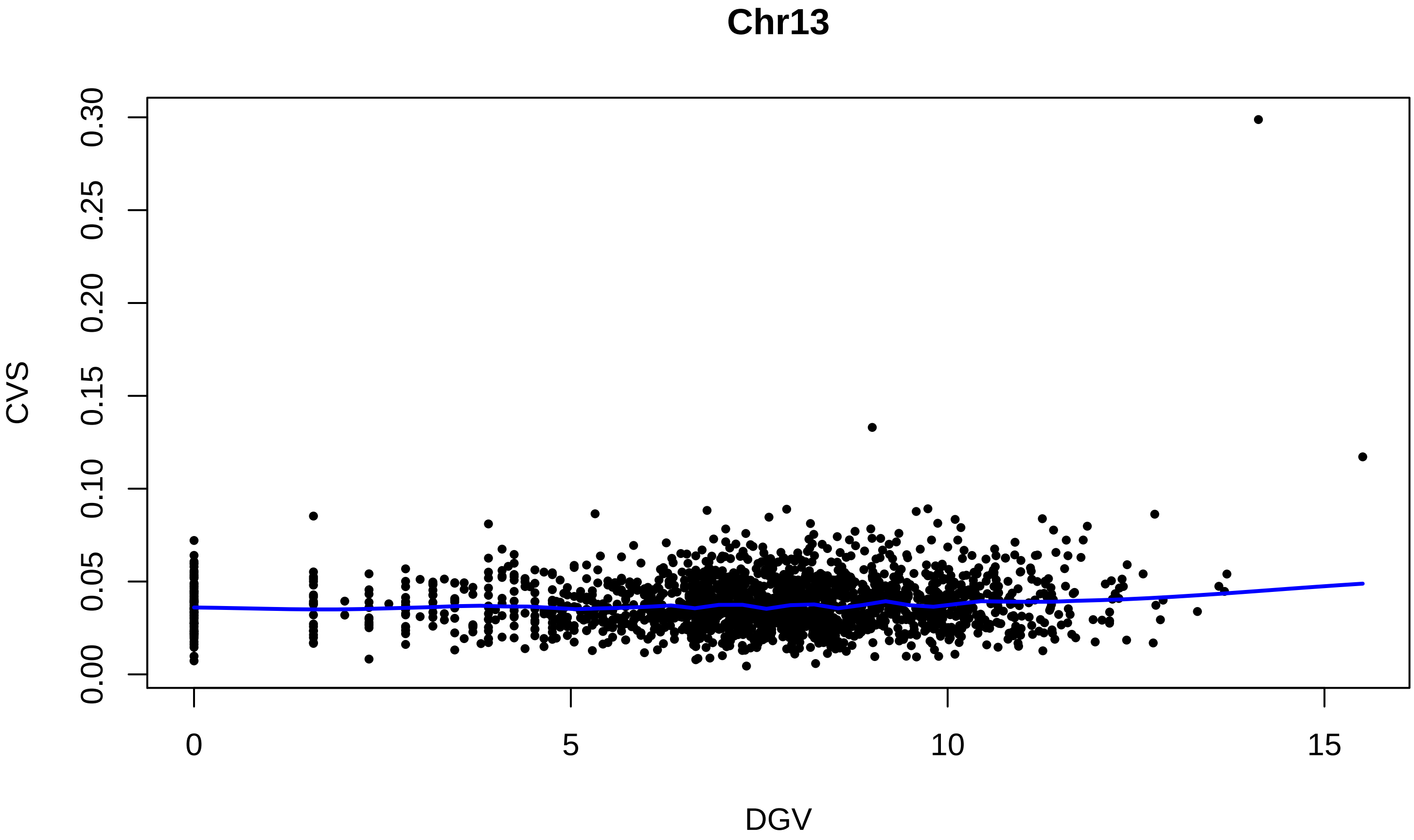

Chr13
CVS
DGV
0.00
0.05
0.10
0.15
0.20
0.25
0.30
0
5
10
15

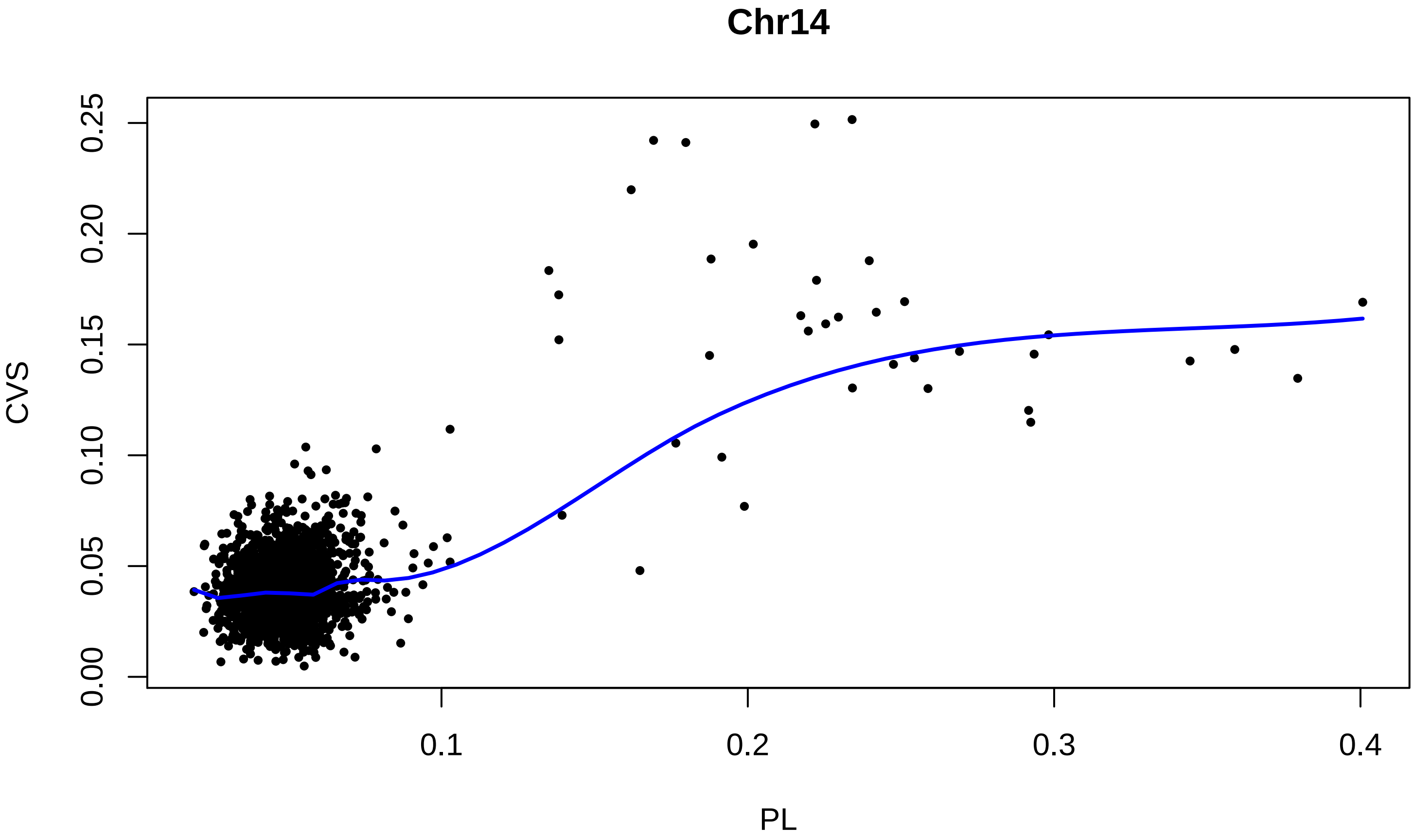

Chr14
CVS
PL
0.00
0.05
0.10
0.15
0.20
0.25
0.1
0.2
0.3
0.4

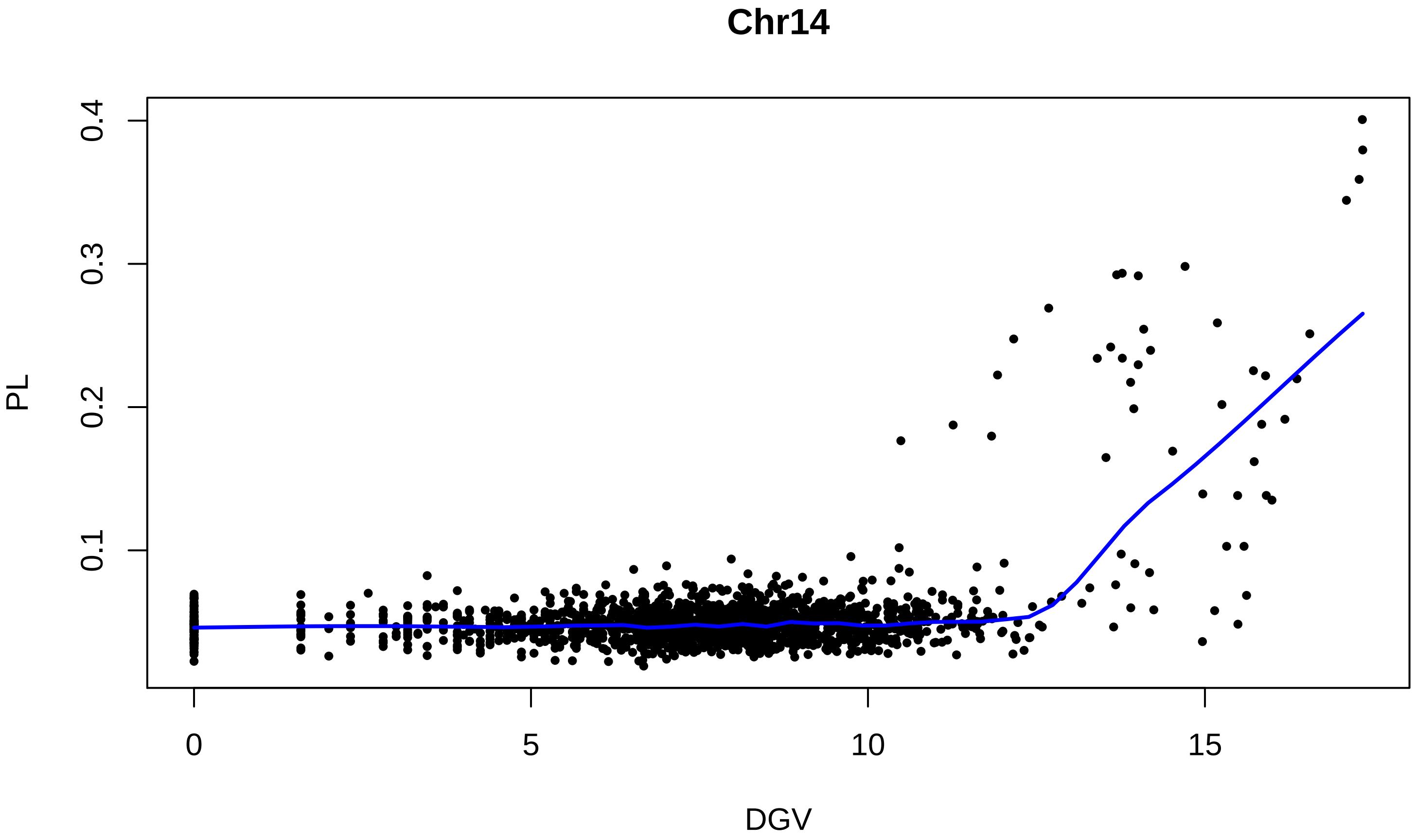

Chr14
PL
0.1
0.2
0.3
0.4
0
5
10
15
DGV

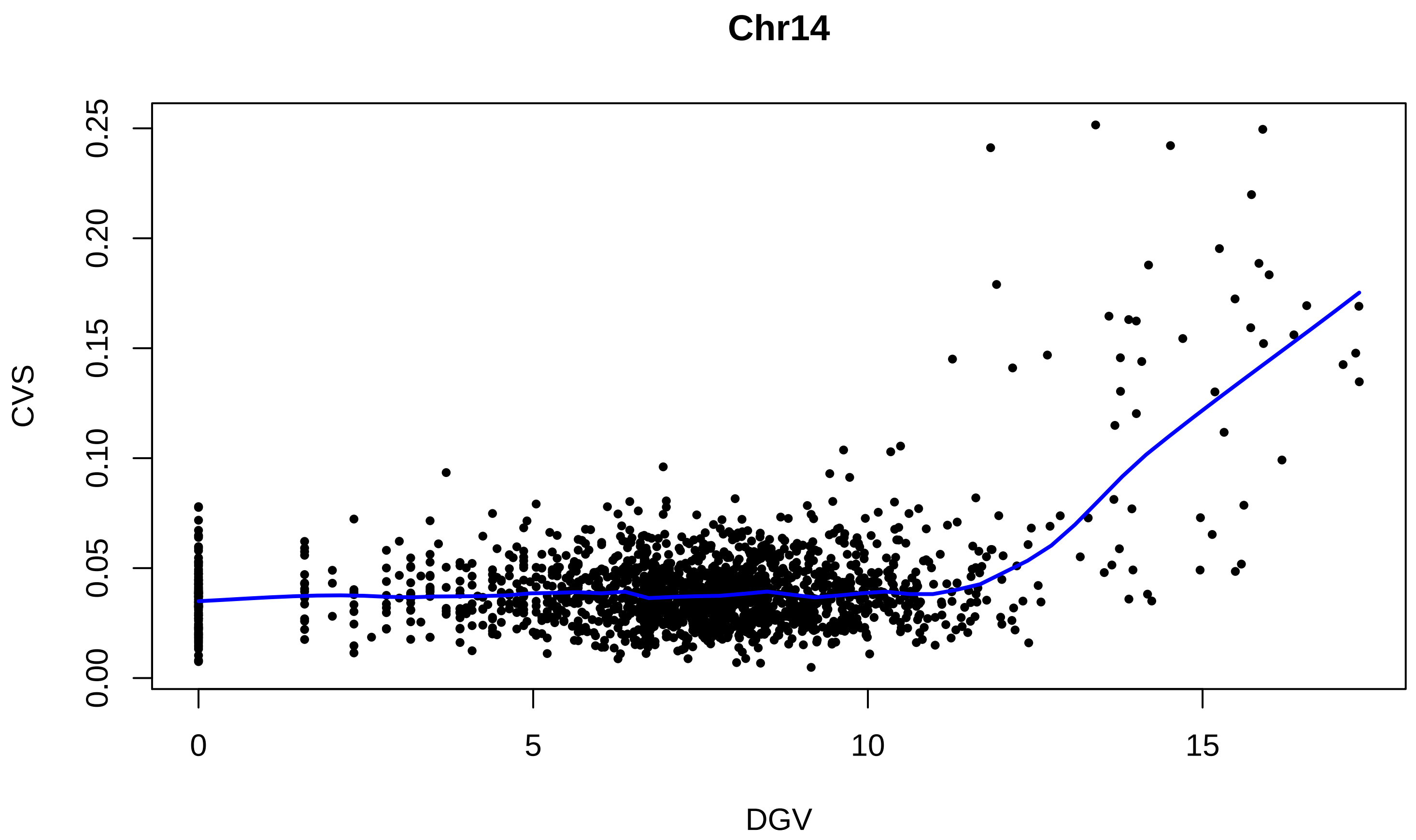
Chr14
CVS
DGV
0.00
0.05
0.10
0.15
0.20
0.25
0
5
10
15

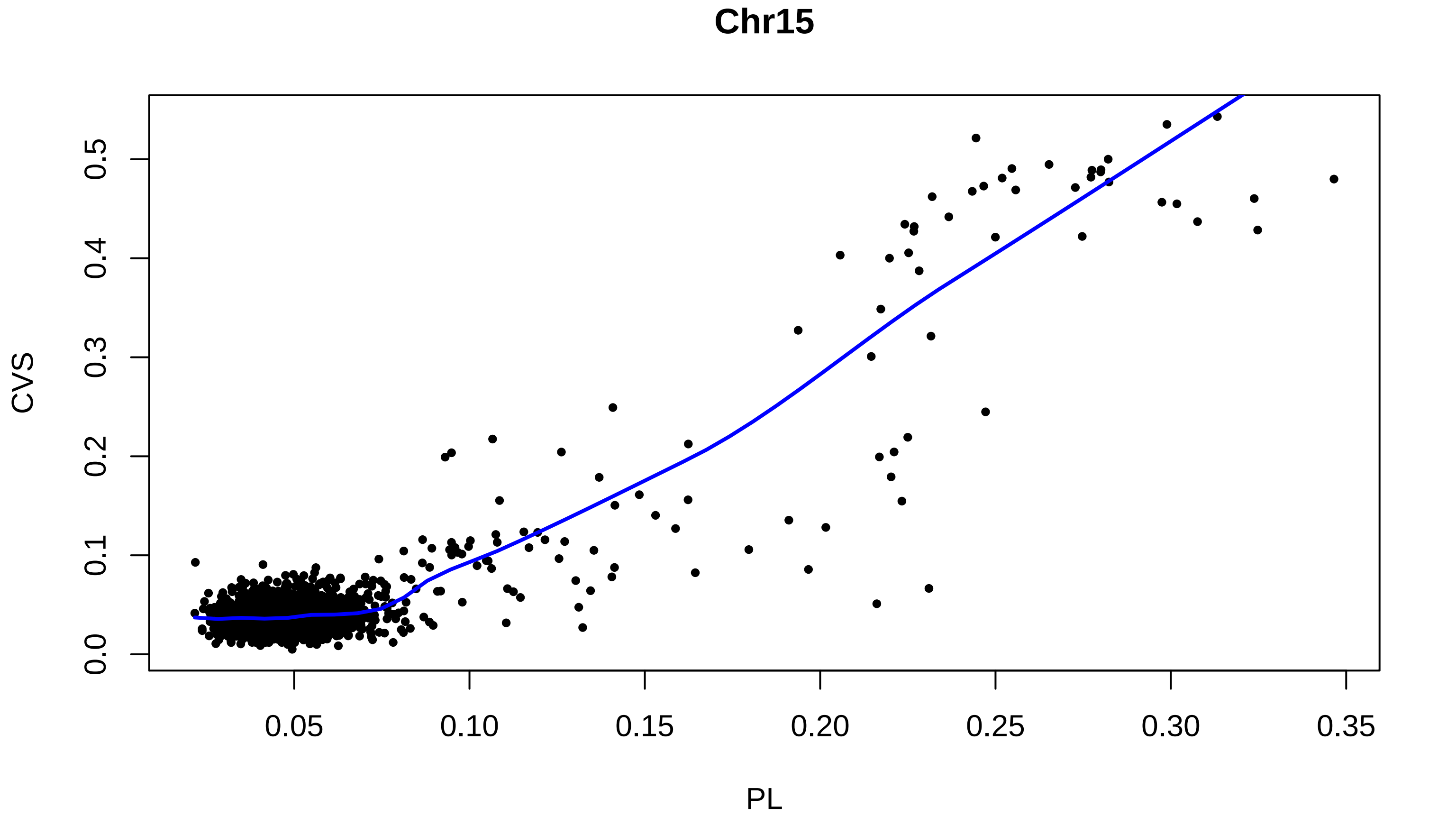

Chr15
CVS
PL
0.0
0.1
0.2
0.3
0.4
0.5
0.05
0.10
0.15
0.20
0.25
0.30
0.35

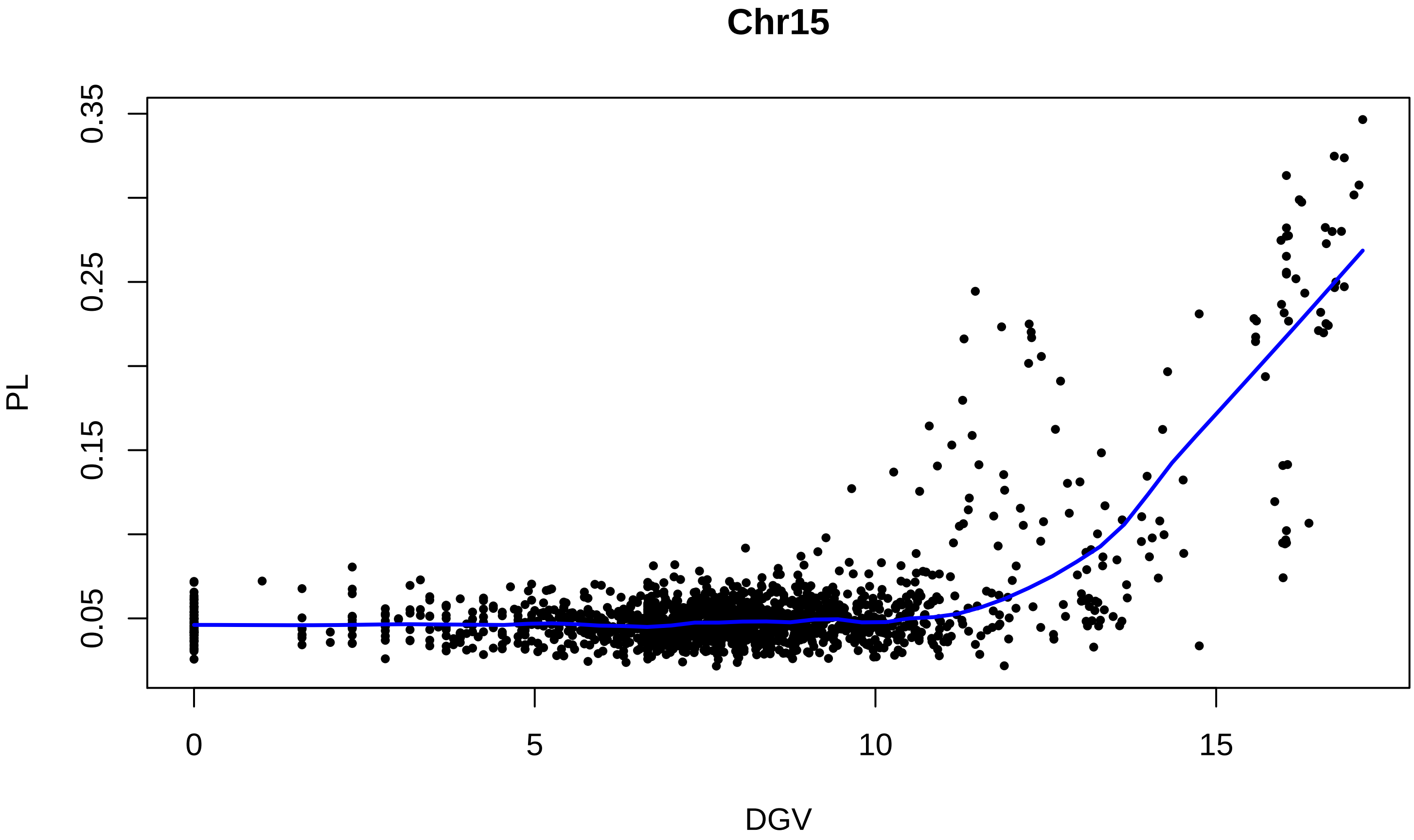

Chr15
PL
DGV
0.05
0.15
0.25
0.35
0
5
10
15

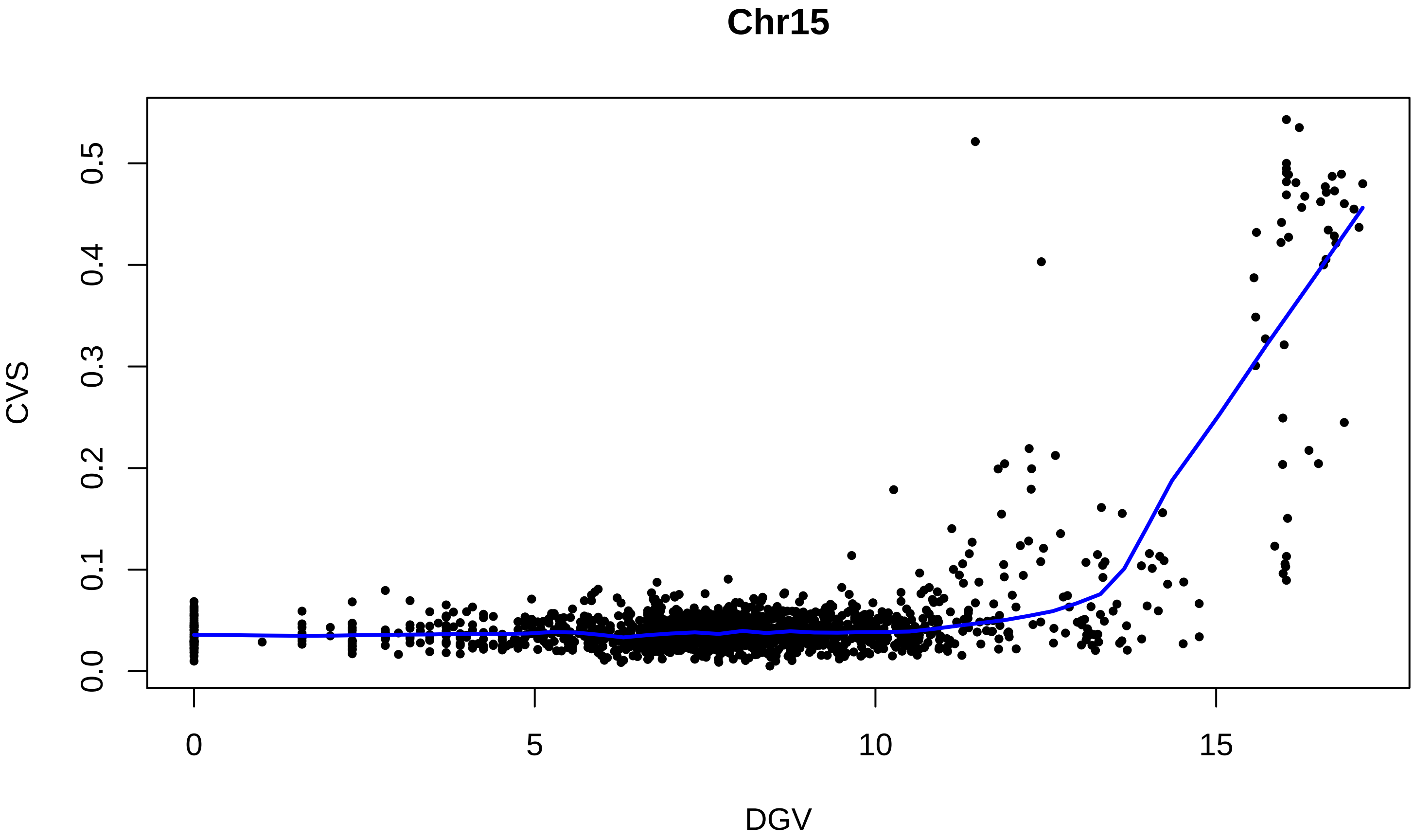

Chr15
CVS
0.0
0.1
0.2
0.3
0.4
0.5
0
5
10
15
DGV

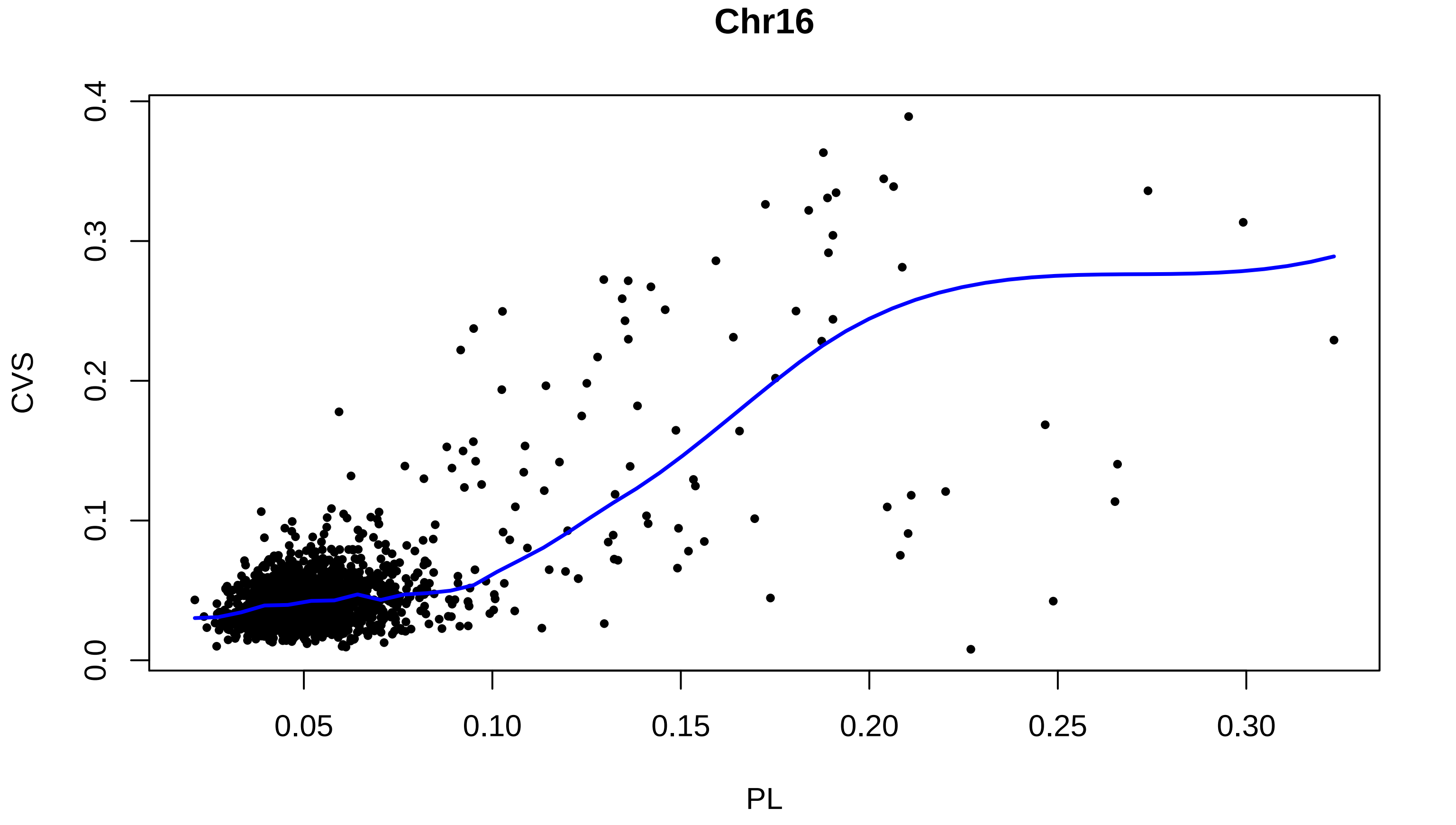

Chr16
CVS
PL
0.0
0.1
0.2
0.3
0.4
0.05
0.10
0.15
0.20
0.25
0.30

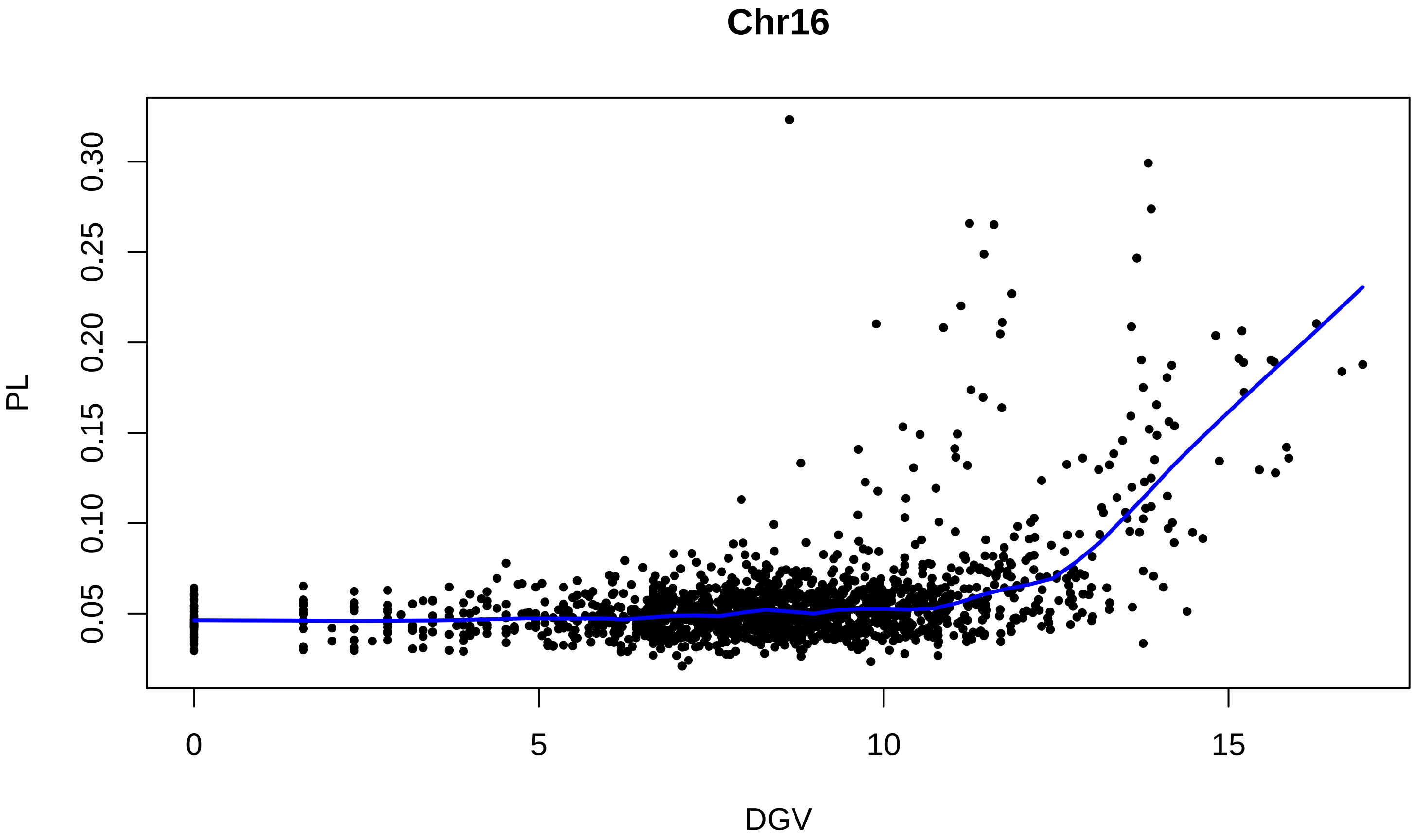

Chr16
PL
0.05
0.10
0.15
0.20
0.25
0.30
DGV
0
5
10
15

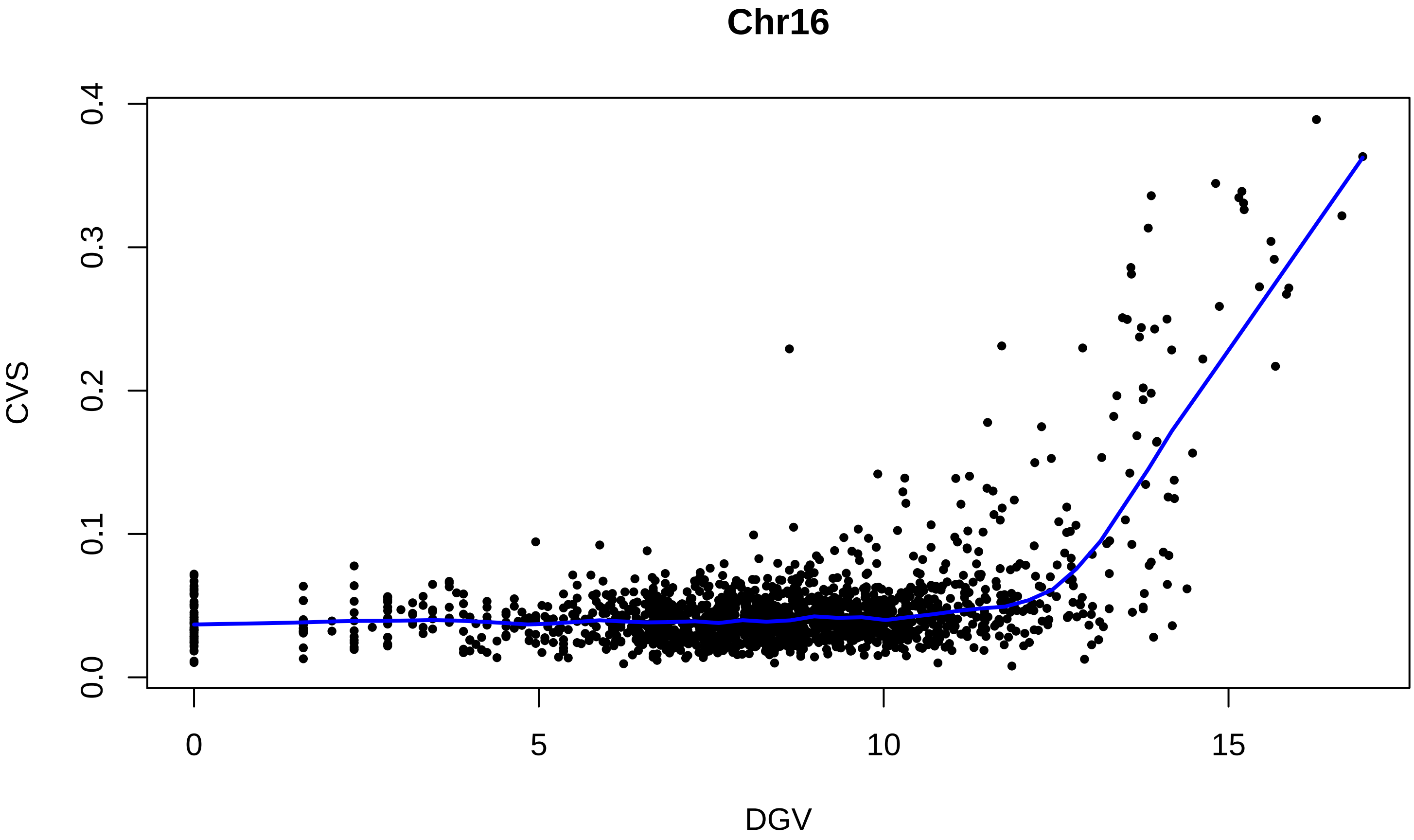

Chr16
CVS
DGV
0.0
0.1
0.2
0.3
0.4
0
5
10
15

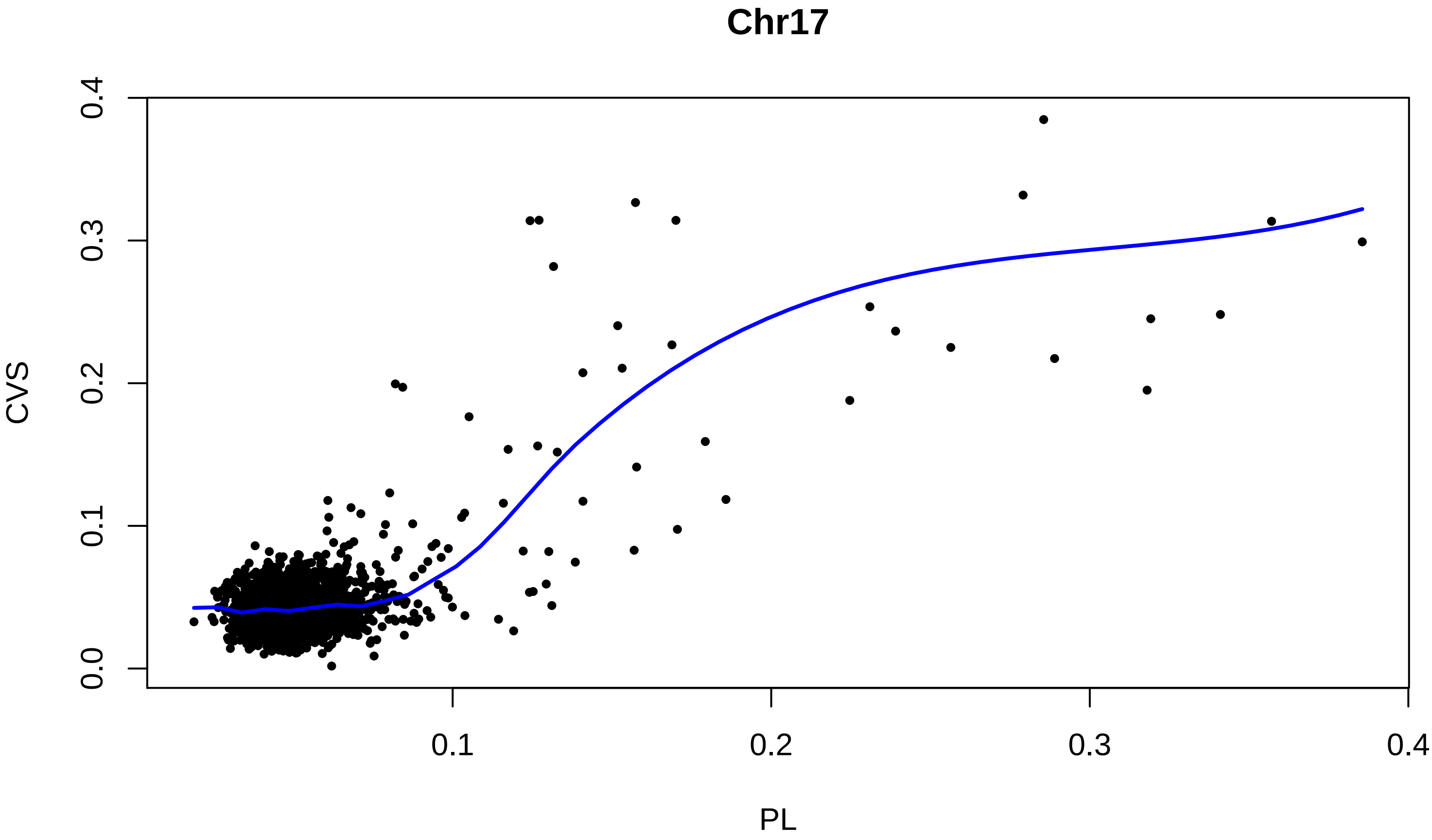

Chr17
CVS
PL
0.0
0.1
0.2
0.3
0.4
0.1
0.2
0.3
0.4

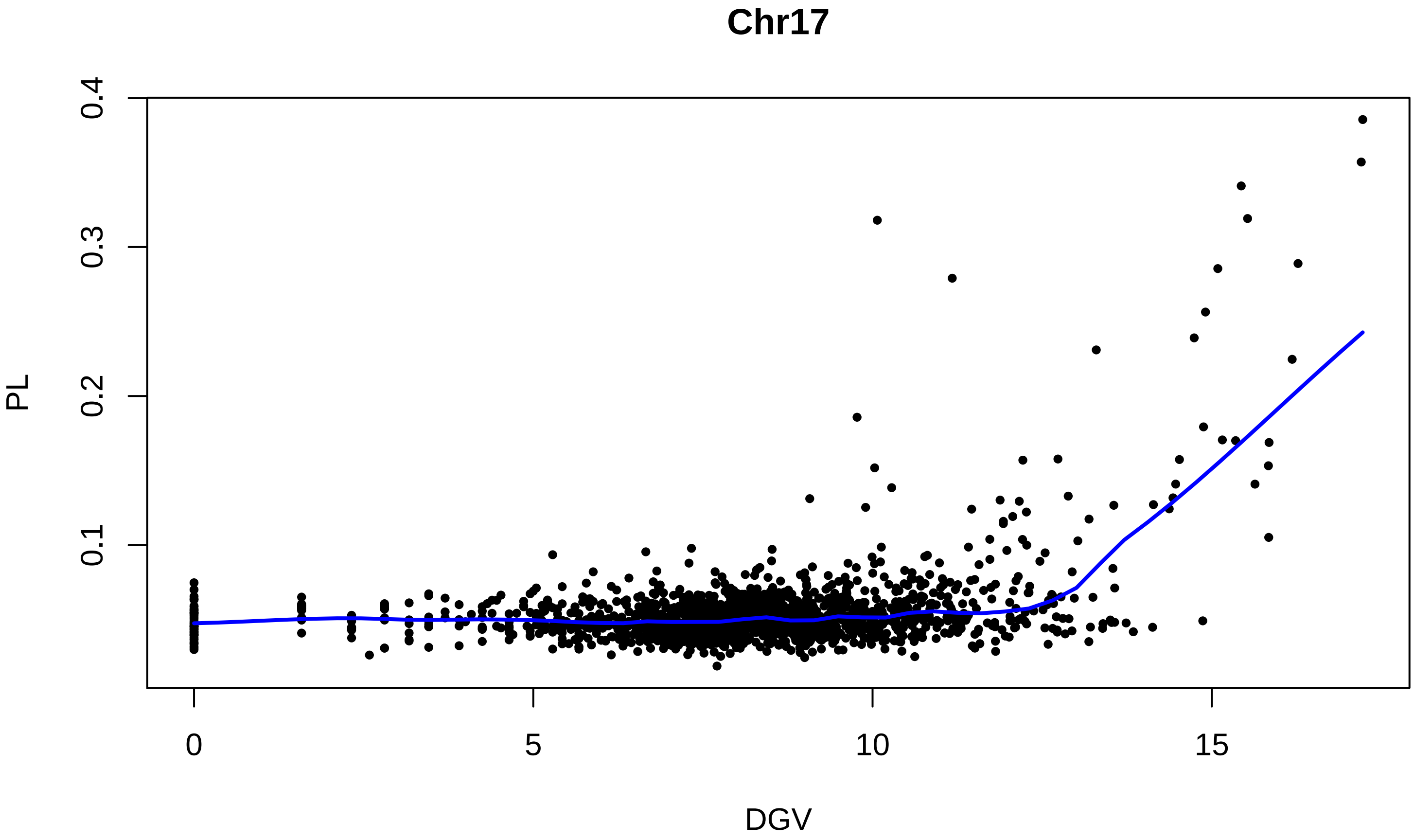
Chr17
PL
0.1
0.2
0.3
0.4
0
5
10
15
DGV

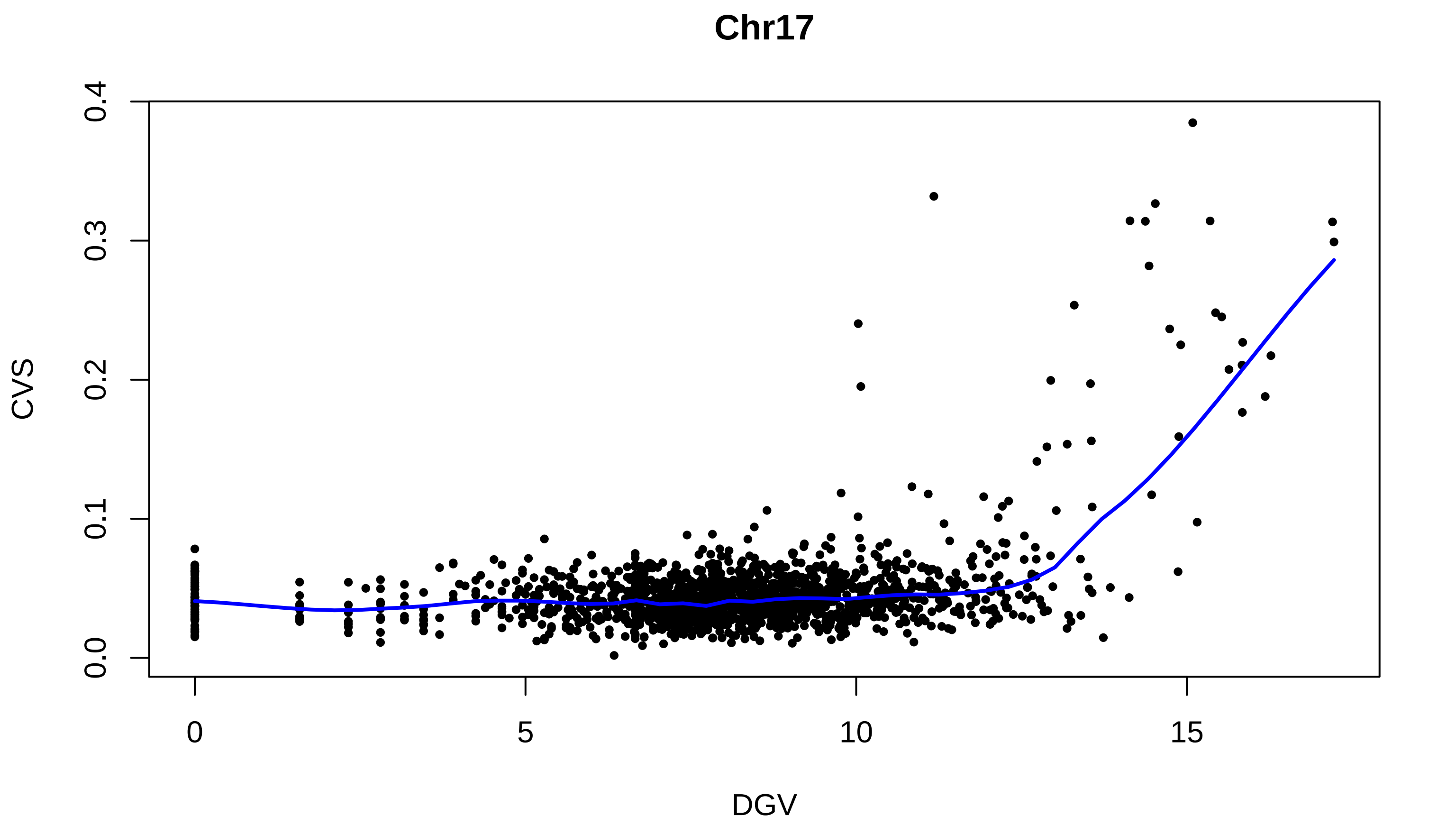

Chr17
CVS
0.0
0.1
0.2
0.3
0.4
0
5
10
15
DGV

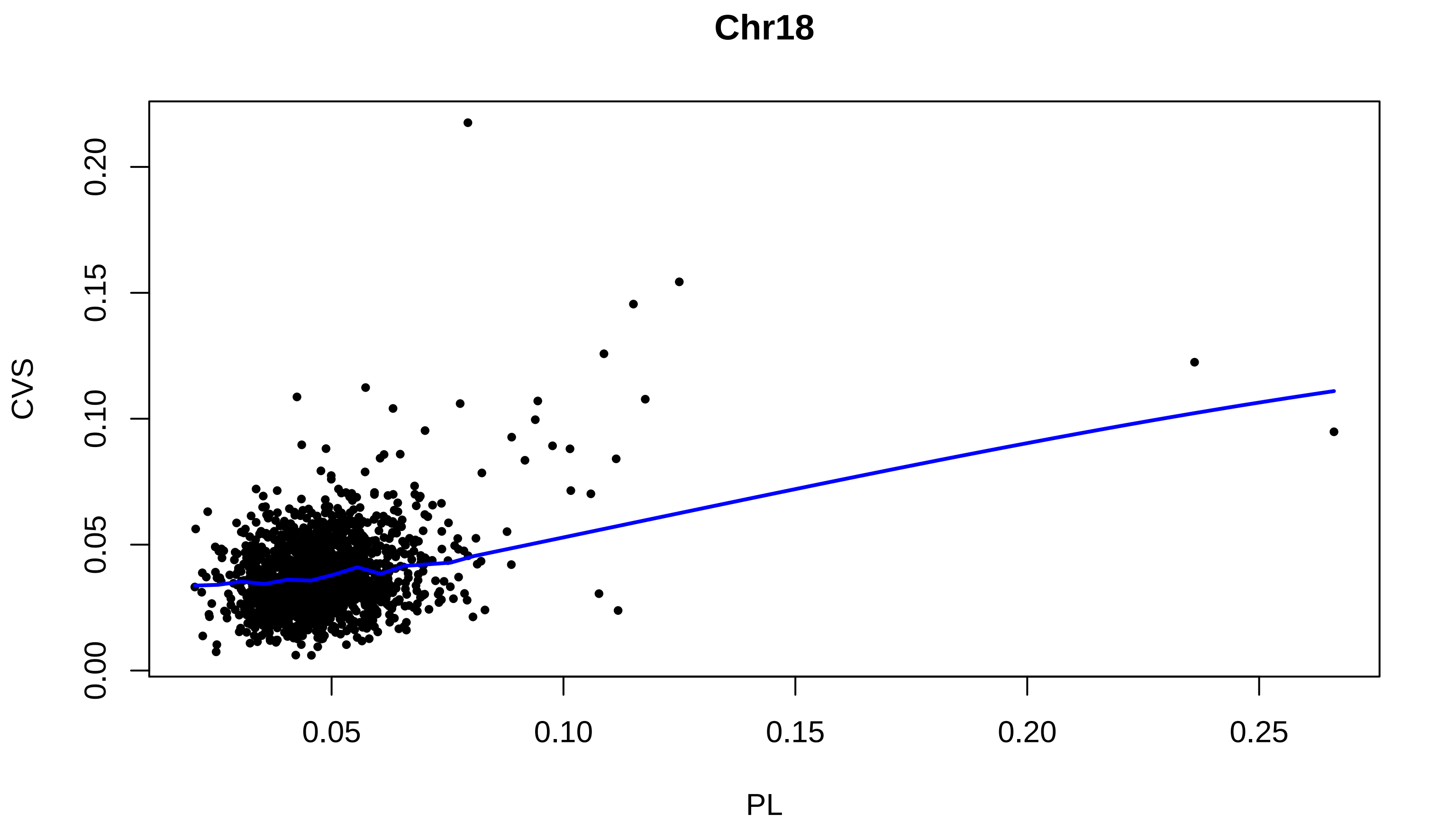

Chr18
CVS
0.00
0.05
0.10
0.15
0.20
0.05
0.10
0.15
0.20
0.25
PL

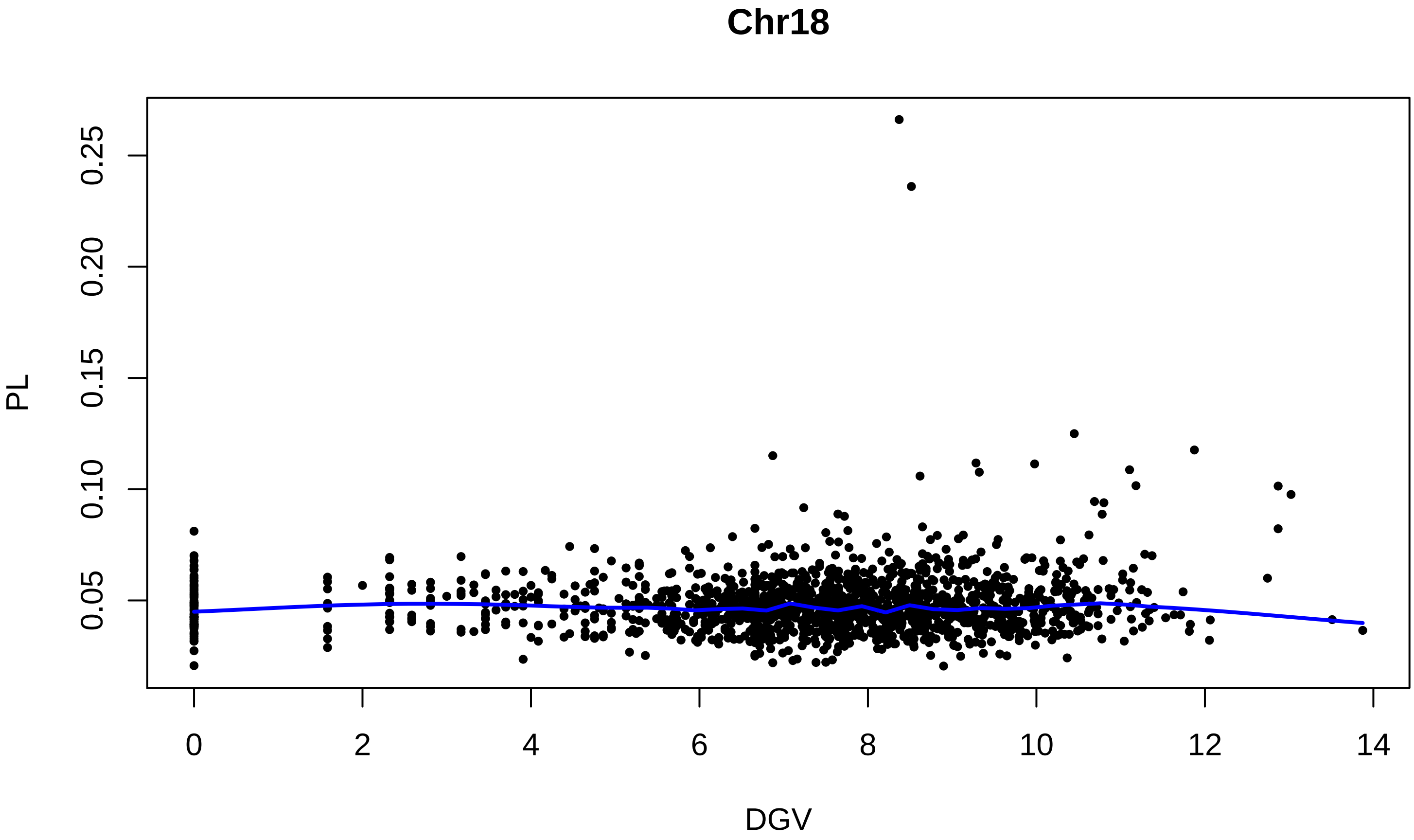
Chr18
PL
0.05
0.10
0.15
0.20
0.25
0
2
4
6
8
10
12
14
DGV

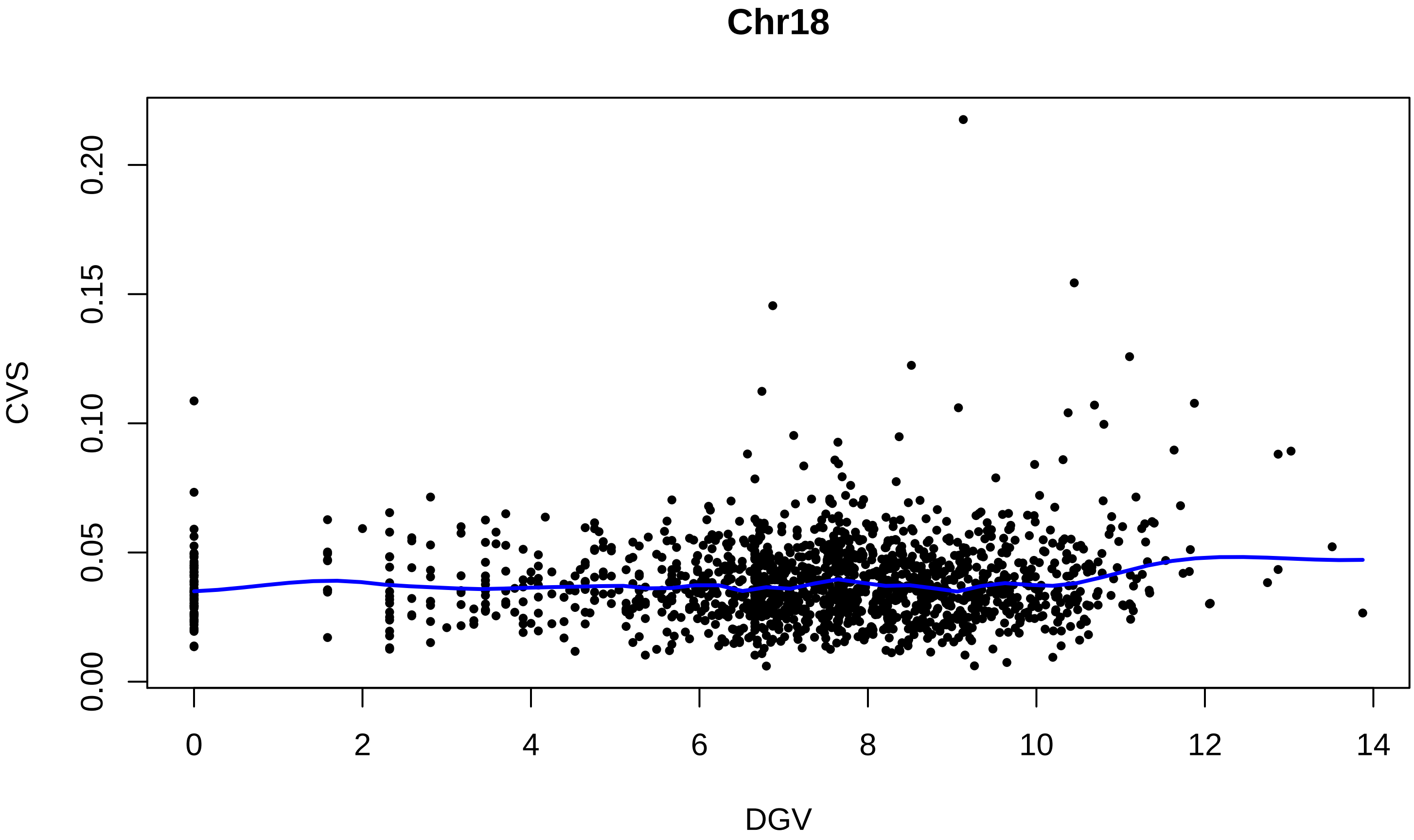
Chr18
CVS
0.00
0.05
0.10
0.15
0.20
0
2
4
6
8
10
12
14
DGV

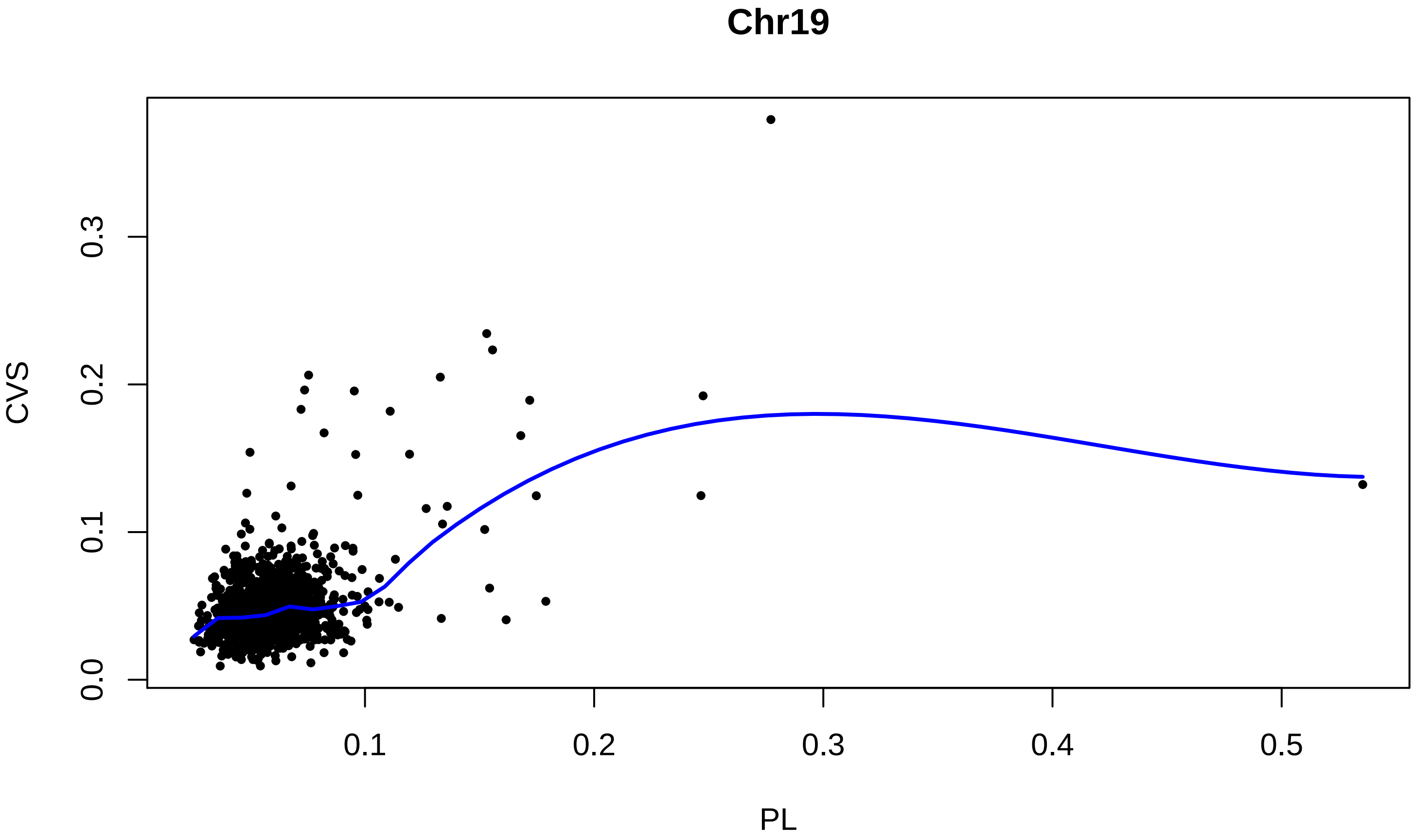

Chr19
CVS
0.0
0.1
0.2
0.3
PL
0.1
0.2
0.3
0.4
0.5

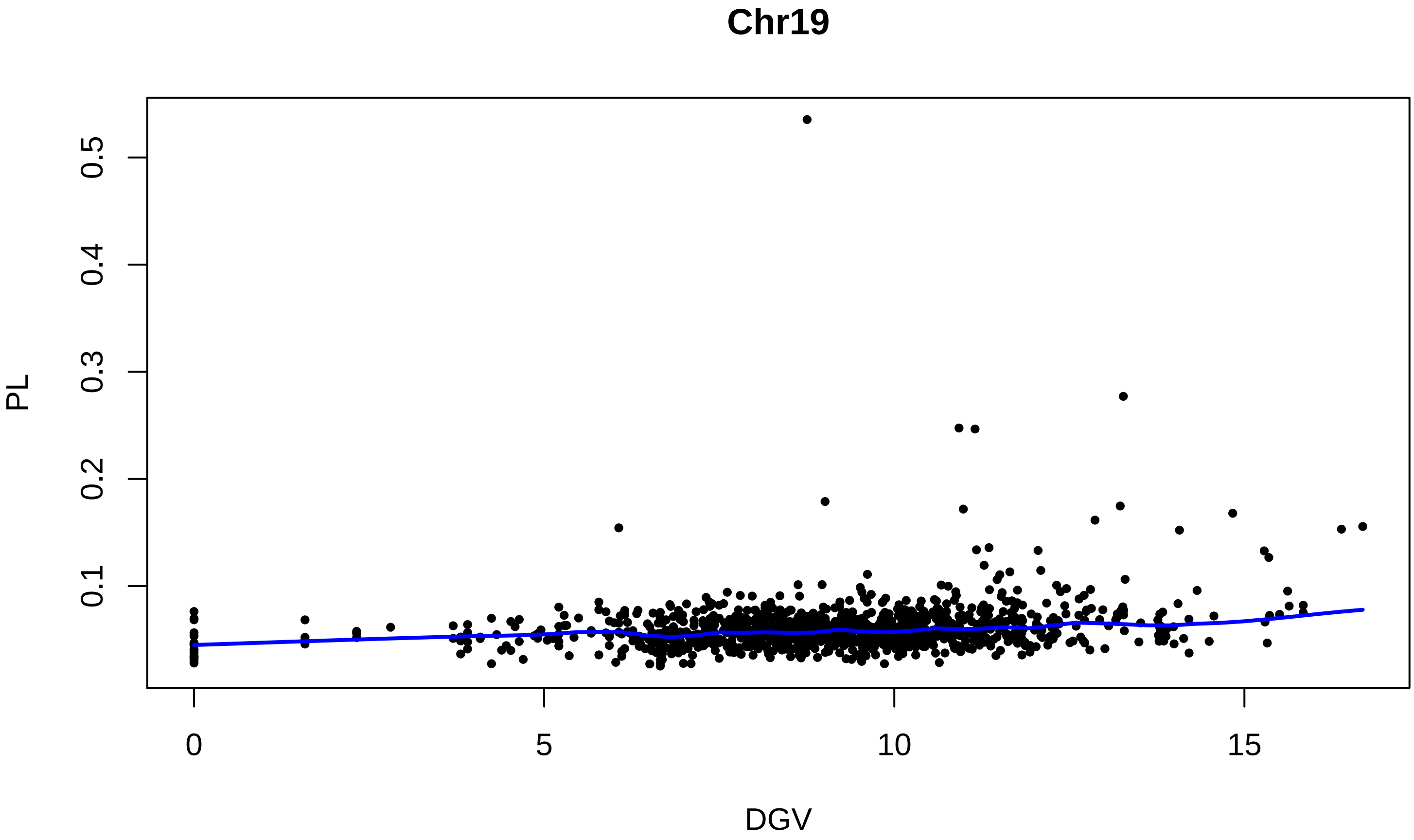
Chr19
PL
DGV
0.1
0.2
0.3
0.4
0.5
0
5
10
15

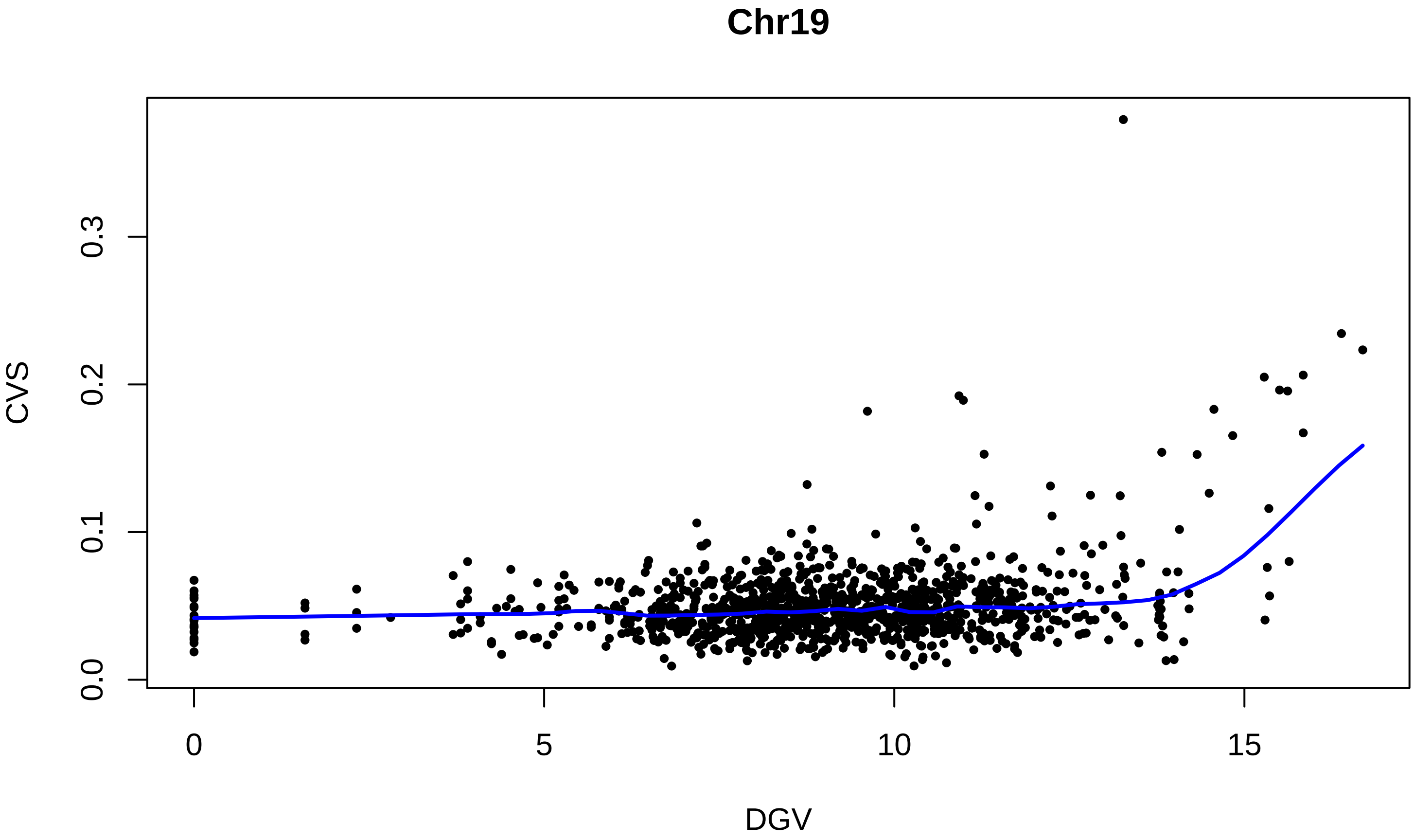
Chr19
CVS
DGV
0.0
0.1
0.2
0.3
0
5
10
15

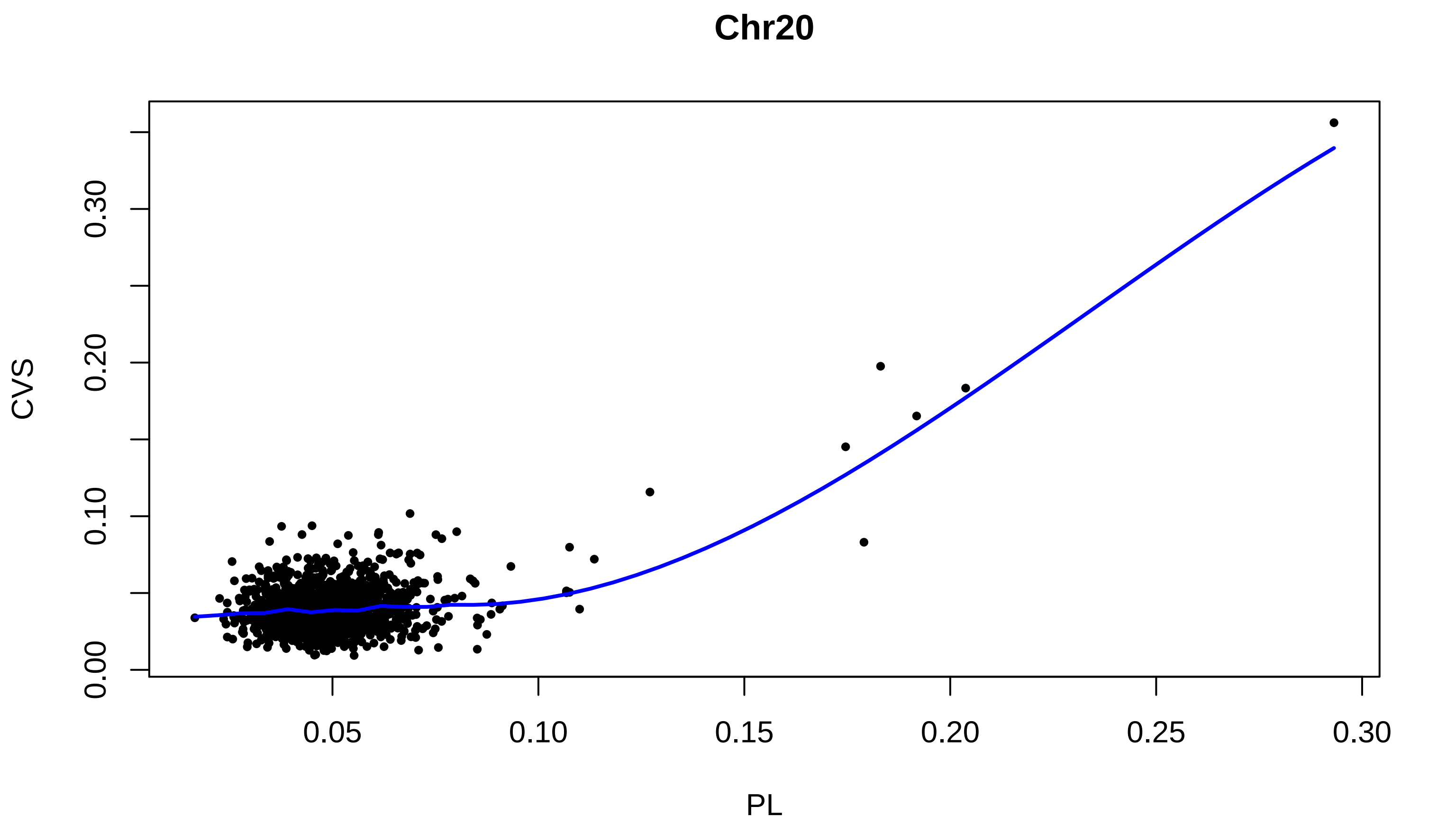

Chr20
CVS
PL
0.00
0.10
0.20
0.30
0.05
0.10
0.15
0.20
0.25
0.30

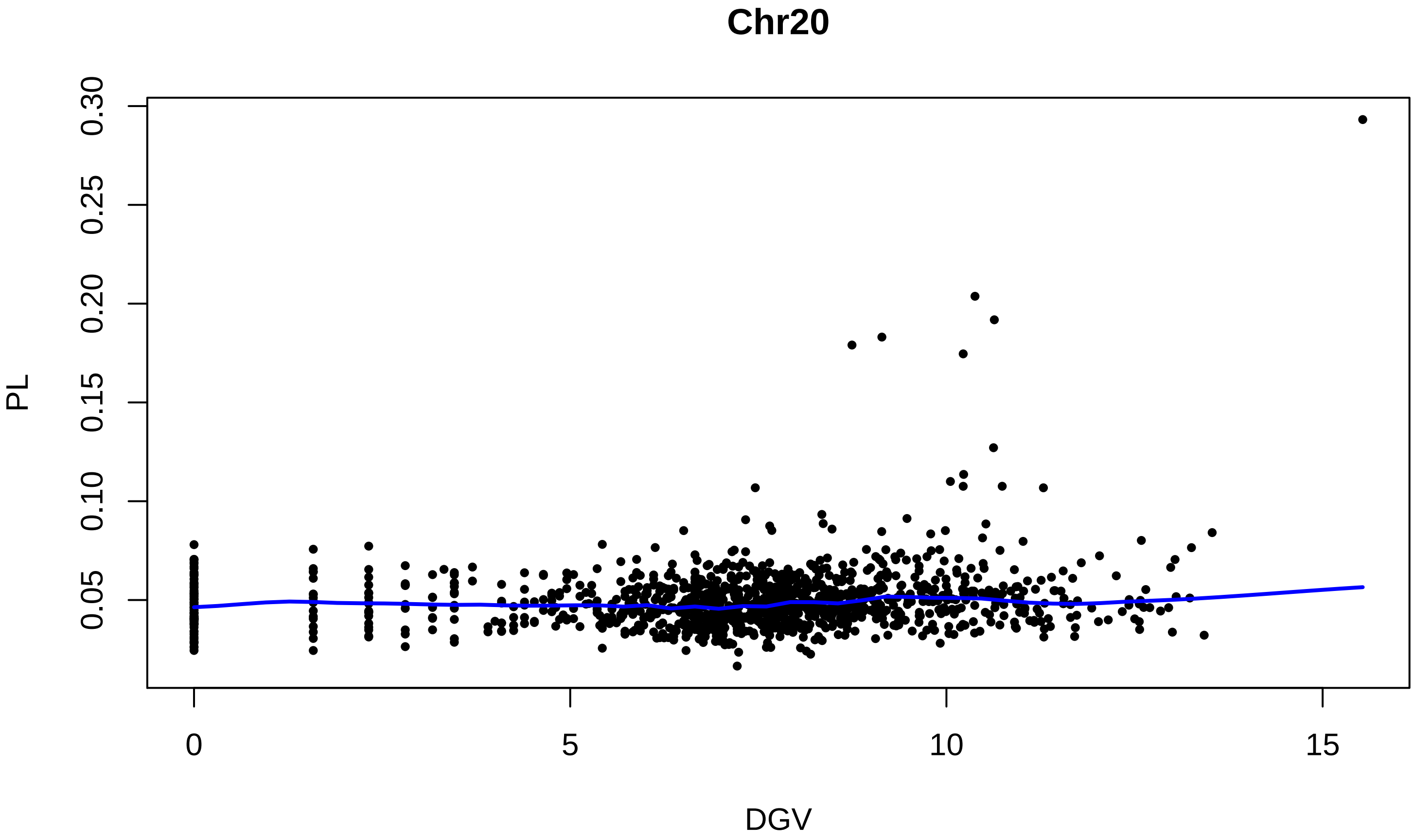

Chr20
PL
DGV
0.05
0.10
0.15
0.20
0.25
0.30
0
5
10
15

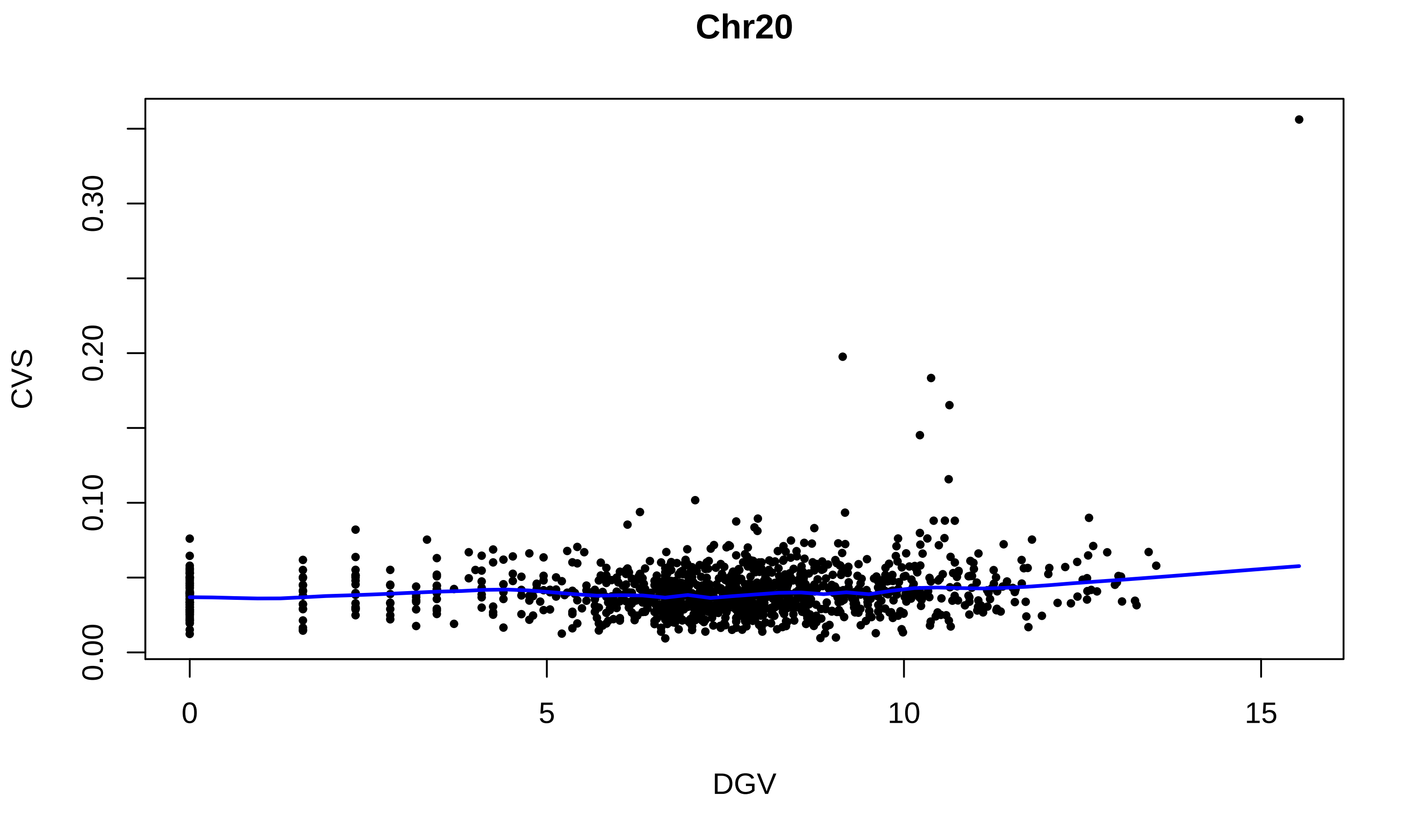

Chr20
CVS
DGV
0.00
0.10
0.20
0.30
0
5
10
15

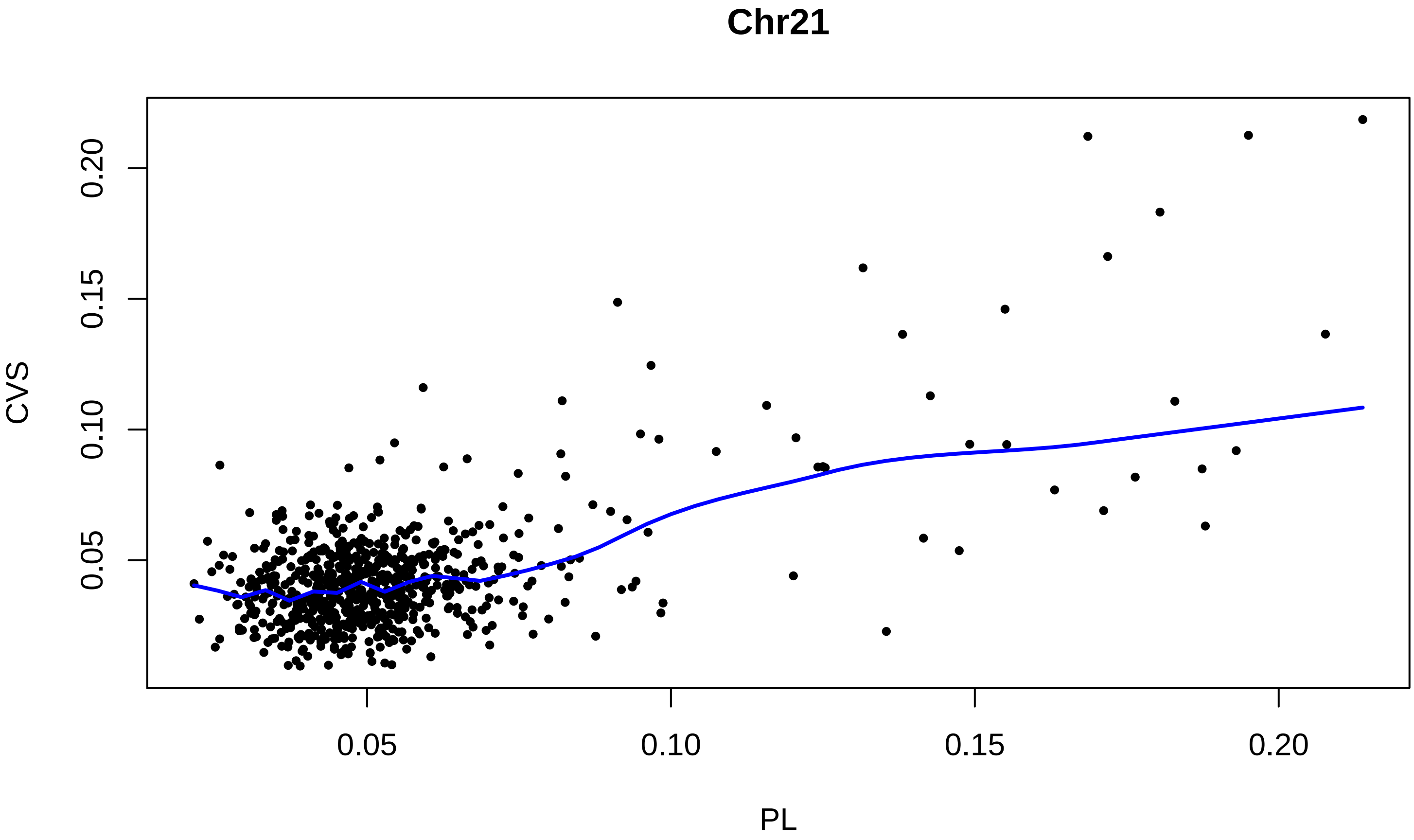
Chr21
CVS
0.05
0.10
0.15
0.20
PL
0.05
0.10
0.15
0.20

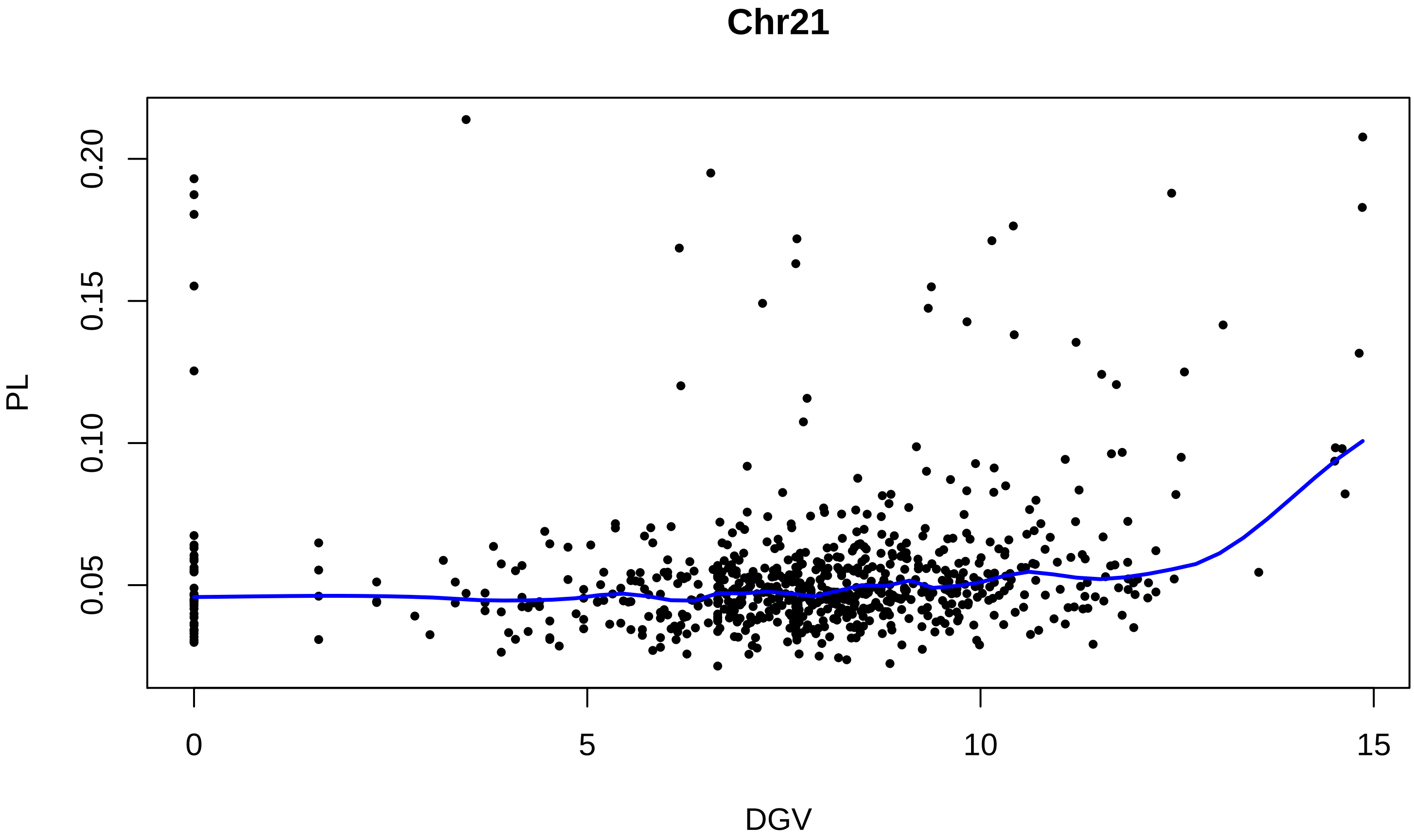

Chr21
PL
0.05
0.10
0.15
0.20
0
5
10
15
DGV

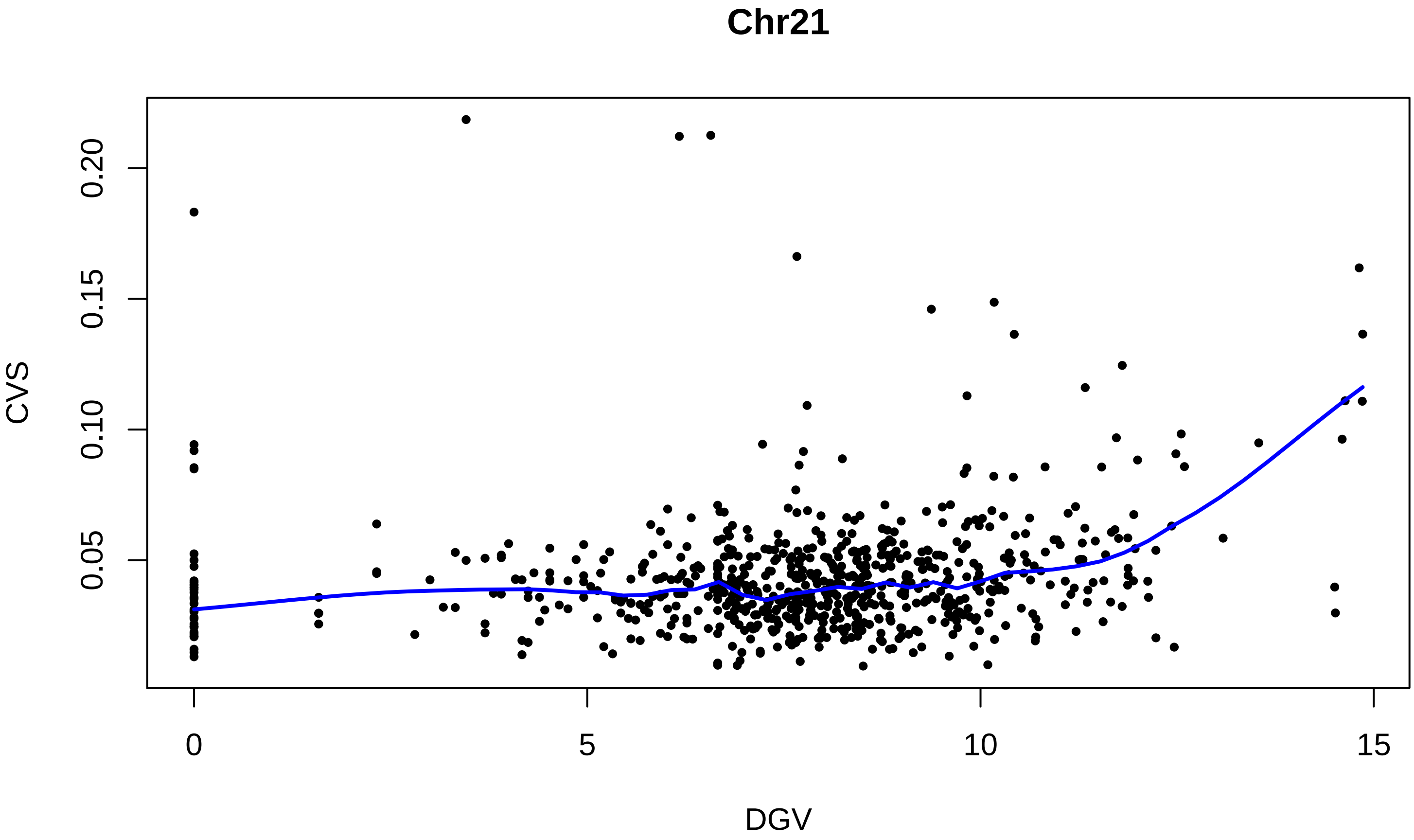

Chr21
CVS
0.05
0.10
0.15
0.20
DGV
0
5
10
15

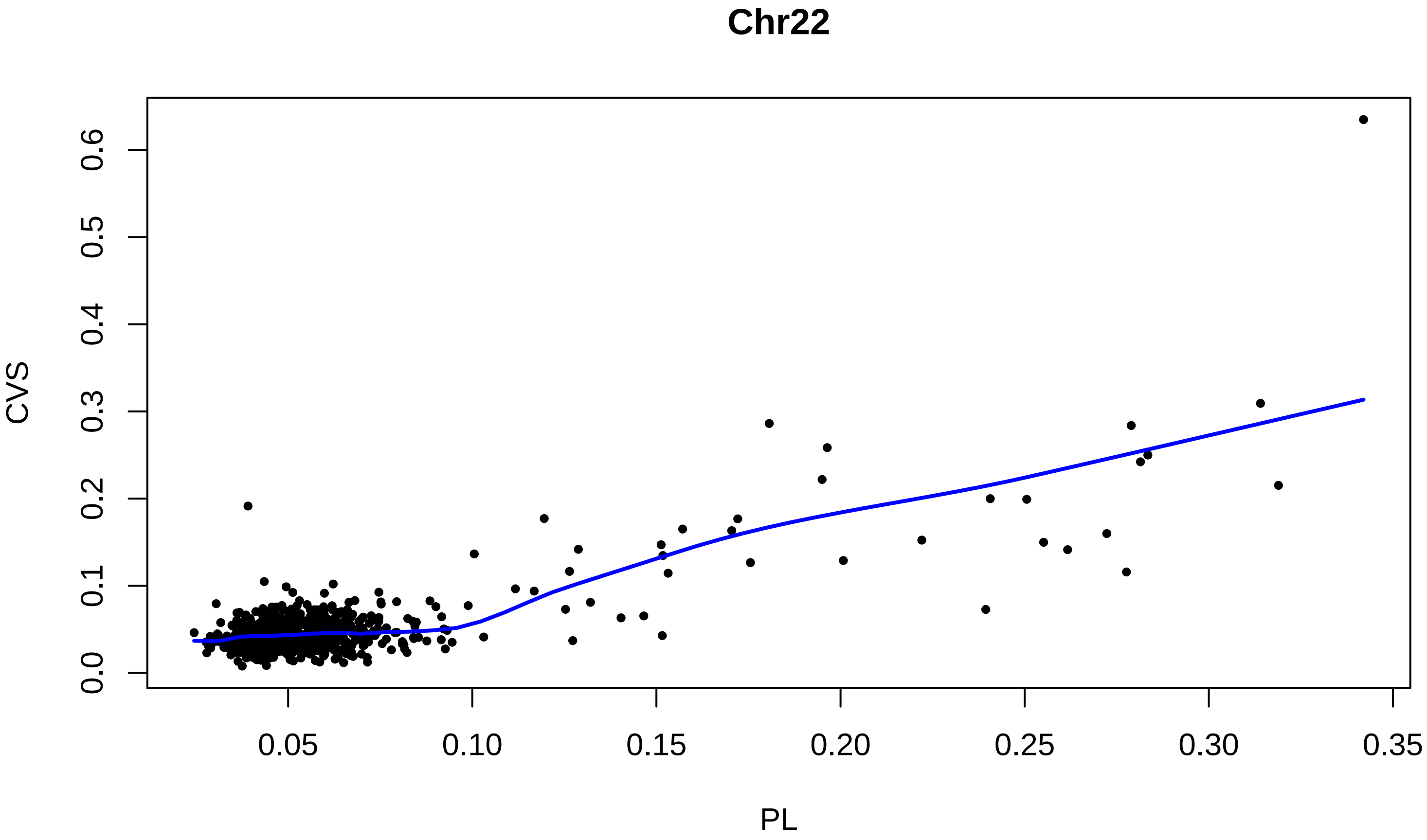

Chr22
CVS
PL
0.0
0.1
0.2
0.3
0.4
0.5
0.6
0.05
0.10
0.15
0.20
0.25
0.30
0.35

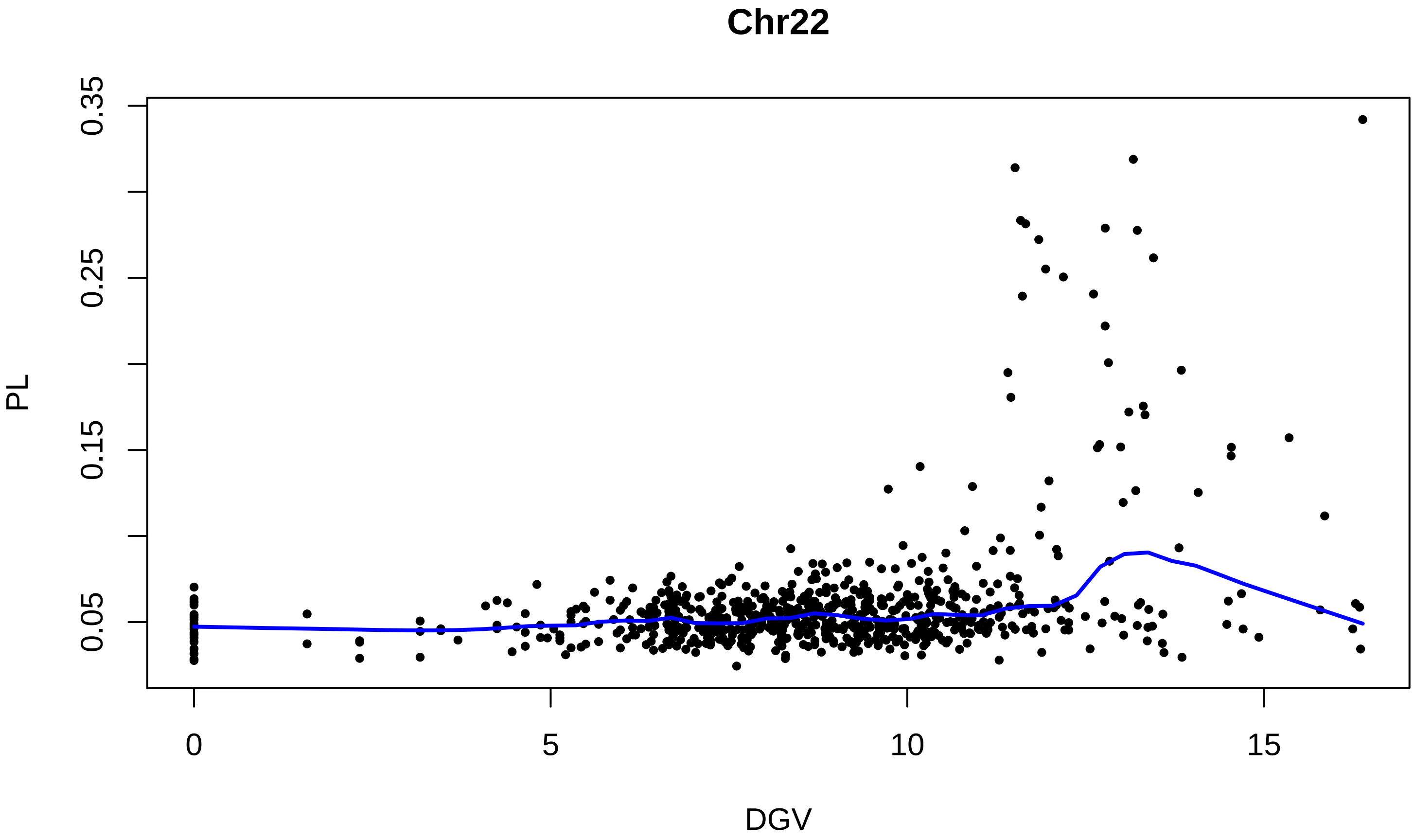

Chr22
PL
DGV
0.05
0.15
0.25
0.35
0
5
10
15

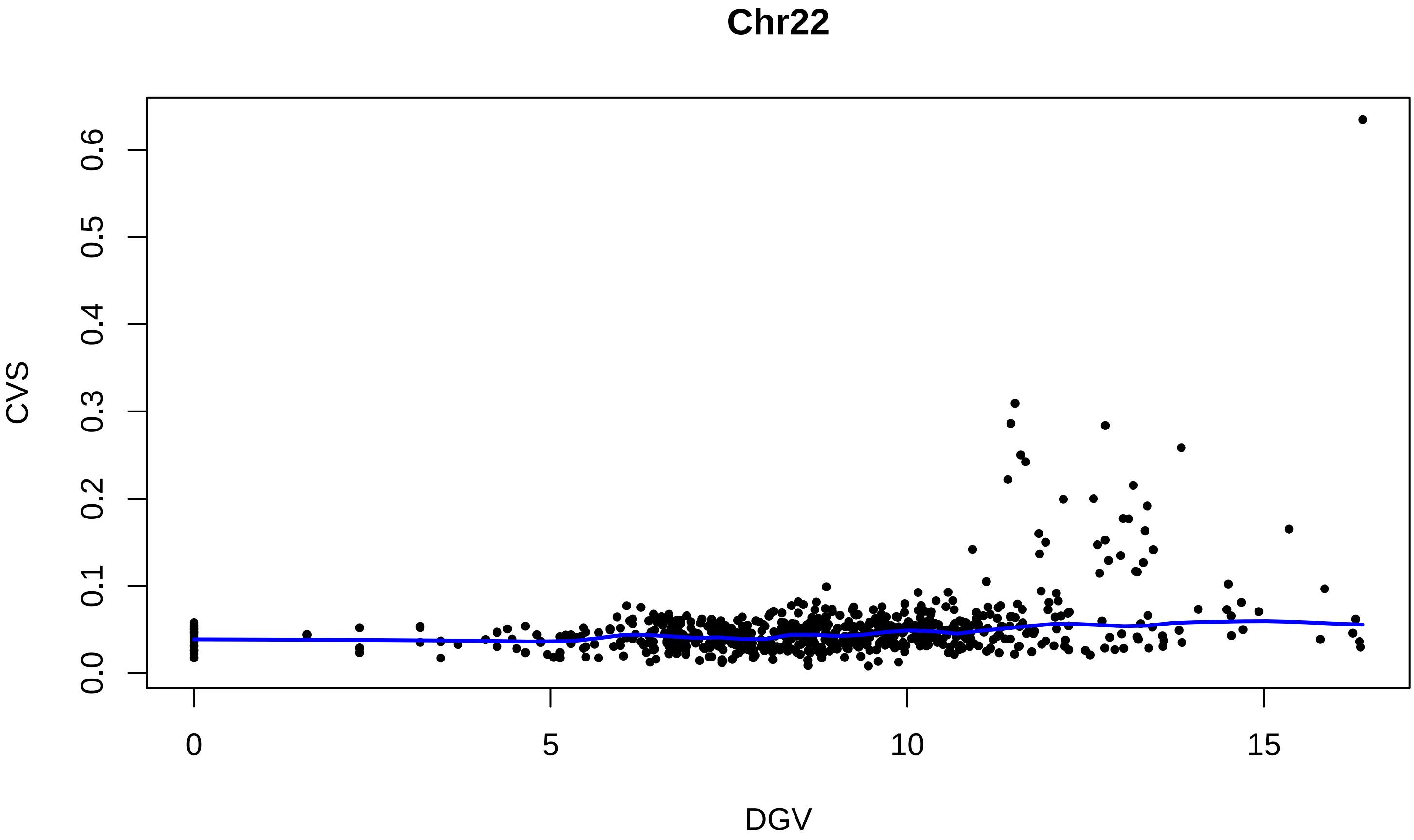
Chr22
CVS
0.0
0.1
0.2
0.3
0.4
0.5
0.6
DGV
0
5
10
15
