# Supplementary material for: High Levels of Sample-to-Sample Variation Confound Data Analysis for Non-Invasive Prenatal Screening of Fetal Microdeletions
Source: PLoS One. 2016 Jun 1;11(6):e0153182. doi: 10.1371/journal.pone.0153182 (PMC4889033; doi:10.1371/journal.pone.0153182)
Supplement: S2 Table — (A).Comparison of the number of structural variants reported in the DGV database between regions in fifteen maternal plasma control libraries displaying high and low cv.RTC (B). Comparison of the number of structural variants reported in the DGV database between regions in six CVS controls libraries displaying high and low cv.RTC. (DOCX) [file pone.0153182.s004.docx]

**Table S2A.** Comparison of the number of structural variants reported in the DGV database between regions in 15 maternal plasma control libraries displaying high and low cv.RTC. ***hi.cv****:* average log2(GVF+1) of regions with high cv.RTC [>=95% quantile of cv.RTC in that chromosome]. ***lo.cv****:* average log2(GVF+1) of regions with cv.RTC < 95% quantile. ***wilcox.pval****:* p value of the Wilcoxon rank sum test comparing log2(GVF+1) of regions with cv.RTC >=95% quantile against log2(GVF+1) of regions with cv.RTC < 95% quantile. ***t.pval****:* two sample t test p value for the same comparison. ***cv.cutoff****:* 95% quantile of cv.RTC in that chromosome

| chr | hi.cv | lo.cv | wilcox.pval | t.pval | cv.cutoff |
| --- | --- | --- | --- | --- | --- |
| 1 | 9.2679 | 7.0518 | 4.3691e-23 | 7.2571e-19 | 0.1561 |
| 2 | 9.1660 | 7.1676 | 1.0788e-27 | 1.5030e-17 | 0.0582 |
| 3 | 8.7296 | 7.1134 | 2.4083e-11 | 1.1157e-10 | 0.0542 |
| 4 | 9.3420 | 7.2594 | 3.2147e-19 | 6.5947e-14 | 0.0554 |
| 5 | 9.5891 | 7.0383 | 3.1126e-25 | 4.2648e-17 | 0.0560 |
| 6 | 8.6984 | 6.7870 | 1.2089e-10 | 2.6169e-09 | 0.0561 |
| 7 | 10.7027 | 7.6536 | 3.4272e-40 | 6.8033e-34 | 0.0623 |
| 8 | 10.4139 | 7.1563 | 1.4946e-30 | 1.9768e-22 | 0.0578 |
| 9 | 11.1271 | 8.0355 | 4.2863e-36 | 1.0885e-20 | 0.1144 |
| 10 | 10.7865 | 7.0729 | 3.6682e-35 | 3.8056e-27 | 0.0610 |
| 11 | 9.3442 | 7.2600 | 2.2695e-16 | 3.6135e-14 | 0.0602 |
| 12 | 8.3065 | 6.9143 | 5.4397e-05 | 0.00015744 | 0.0592 |
| 13 | 8.4579 | 7.2505 | 8.5257e-06 | 0.00039566 | 0.0553 |
| 14 | 11.0547 | 7.0450 | 9.6492e-21 | 4.9047e-15 | 0.0613 |
| 15 | 14.3831 | 7.4276 | 7.5239e-49 | 1.2402e-45 | 0.0980 |
| 16 | 12.9721 | 7.9685 | 2.5215e-40 | 2.4411e-38 | 0.0949 |
| 17 | 11.9556 | 7.8278 | 4.3143e-29 | 7.7205e-22 | 0.0717 |
| 18 | 7.8297 | 7.1439 | 0.0062096 | 0.021781 | 0.0560 |
| 19 | 10.8837 | 8.9570 | 1.3536e-07 | 4.0818e-07 | 0.0771 |
| 20 | 8.0272 | 7.0619 | 3.0410e-05 | 0.032253 | 0.0603 |
| 21 | 8.8285 | 7.6136 | 0.0040725 | 0.11931 | 0.0848 |
| 22 | 12.3549 | 8.5463 | 1.1885e-16 | 8.1422e-19 | 0.1201 |

**Table S2B.** Comparison of the number of structural variants reported in the DGV database between regions in 6 CVS controls libraries displaying high and low cv.RTC. ***hi.cv****:* average log2(GVF+1) of regions with high cv.RTC [>=95% quantile of cv.RTC in that chromosome]. ***lo.cv****:* average log2(GVF+1) of regions with cv.RTC < 95% quantile. ***wilcox.pval****:* p value of the Wilcoxon rank sum test comparing log2(GVF+1) of regions with cv.RTC >=95% quantile against log2(GVF+1) of regions with cv.RTC < 95% quantile. ***t.pval****:* two sample t test p value for the same comparison. ***cv.cutoff****:* 95% quantile of cv.RTC in that chromosome

| chr | hi.cv | lo.cv | wilcox.pval | t.pval | cv.cutoff |
| --- | --- | --- | --- | --- | --- |
| 1 | 10.8468 | 6.9683 | 5.8521e-63 | 2.9434e-45 | 0.0602 |
| 2 | 8.8436 | 7.1845 | 2.0879e-18 | 3.8204e-14 | 0.0515 |
| 3 | 8.3456 | 7.1336 | 1.1180e-06 | 5.6316e-07 | 0.0489 |
| 4 | 9.2539 | 7.2640 | 5.0471e-15 | 5.9690e-13 | 0.0496 |
| 5 | 9.1999 | 7.0589 | 3.1586e-15 | 3.8729e-14 | 0.0510 |
| 6 | 8.7338 | 6.7851 | 3.8008e-11 | 9.1957e-11 | 0.0506 |
| 7 | 10.1487 | 7.6828 | 7.3485e-26 | 7.5892e-19 | 0.0563 |
| 8 | 10.4422 | 7.1548 | 1.8327e-30 | 2.1539e-22 | 0.0524 |
| 9 | 11.1810 | 8.0326 | 3.5712e-38 | 8.7859e-25 | 0.0677 |
| 10 | 9.4629 | 7.1429 | 3.18215e-18 | 1.0018e-15 | 0.0534 |
| 11 | 9.3602 | 7.2591 | 6.6635e-16 | 5.7751e-12 | 0.0530 |
| 12 | 7.9654 | 6.9323 | 0.00021363 | 0.0049263 | 0.0519 |
| 13 | 7.9916 | 7.2752 | 0.045510 | 0.012122 | 0.0486 |
| 14 | 10.9850 | 7.0487 | 1.7108e-18 | 5.8681e-14 | 0.0544 |
| 15 | 14.5519 | 7.4187 | 9.8688e-50 | 7.1265e-49 | 0.0900 |
| 16 | 13.1100 | 7.9612 | 5.8538e-42 | 4.2579e-41 | 0.0845 |
| 17 | 11.6497 | 7.8440 | 7.0143e-24 | 6.2000e-17 | 0.0618 |
| 18 | 8.2927 | 7.1195 | 0.00012163 | 0.00026707 | 0.0495 |
| 19 | 11.5253 | 8.9232 | 8.7997e-10 | 8.4315e-09 | 0.0671 |
| 20 | 8.3249 | 7.0462 | 0.00013026 | 0.0017341 | 0.0537 |
| 21 | 9.1390 | 7.5969 | 0.00041636 | 0.053673 | 0.0627 |
| 22 | 12.8436 | 8.5204 | 2.2163e-19 | 1.0255e-23 | 0.0895 |
