# Supplementary material for: High Levels of Sample-to-Sample Variation Confound Data Analysis for Non-Invasive Prenatal Screening of Fetal Microdeletions
Source: PLoS One. 2016 Jun 1;11(6):e0153182. doi: 10.1371/journal.pone.0153182 (PMC4889033; doi:10.1371/journal.pone.0153182)
Supplement: S3 Table — (A).Comparison of the coefficient of variation of relative read count between regions in fifteen plasma controls libraries displaying high and low numbers of reported structural variants in the DGV database. (B). Comparison of the coefficient of variation of relative read count between regions in six CVS controls libraries displaying high and low numbers of reported structural variants in the DGV database. (DOCX) [file pone.0153182.s005.docx]

**Table S3A:** Comparison of the coefficient of variation of relative read count between regions in 15 plasma controls libraries displaying high and low numbers of reported structural variants in the DGV database. ***hi.dgv****:* average cv.RTC of regions with high GVF [>=95% quantile of the GVF in that chromosome]. ***lo.dgv****:* average cv.RTC of regions with GVF < 95% quantile. wilcox.pval: p value of the Wilcoxon rank sum test comparing cv.RTC of regions with GVF >=95% quantile against cv.RTC of regions with GVF < 95% quantile. ***t.pval****:* two sample t test p value for the same comparison. ***dgv.cutoff****:* 95% quantile of the log2(GVF+1) in that chromosome

| chr | hi.dgv | lo.dgv | wilcox.pval | t.pval | dgv.cutoff |
| --- | --- | --- | --- | --- | --- |
| 1 | 0.1209 | 0.0637 | 1.6504e-30 | 8.7919e-18 | 11.6475 |
| 2 | 0.0586 | 0.0412 | 2.5977e-18 | 4.6825e-11 | 10.8597 |
| 3 | 0.0572 | 0.0395 | 4.8491e-13 | 2.8327e-07 | 10.7673 |
| 4 | 0.0673 | 0.0399 | 3.2488e-14 | 2.4354e-08 | 11.1087 |
| 5 | 0.0716 | 0.0399 | 6.4425e-22 | 2.0821e-13 | 11.1724 |
| 6 | 0.0598 | 0.0403 | 1.1202e-18 | 1.0076e-08 | 10.9848 |
| 7 | 0.0692 | 0.0420 | 2.6274e-16 | 3.2067e-09 | 11.8331 |
| 8 | 0.0970 | 0.0410 | 3.7637e-23 | 3.9490e-11 | 11.4012 |
| 9 | 0.1059 | 0.0464 | 8.9171e-29 | 1.3932e-13 | 11.9397 |
| 10 | 0.0777 | 0.0414 | 1.0675e-29 | 8.0814e-12 | 11.4779 |
| 11 | 0.0573 | 0.0420 | 4.6167e-09 | 4.8857e-05 | 11.3922 |
| 12 | 0.0619 | 0.0414 | 1.1880e-12 | 5.9112e-06 | 11.0287 |
| 13 | 0.0501 | 0.0400 | 9.0830e-09 | 9.9267e-05 | 10.8639 |
| 14 | 0.1207 | 0.0414 | 7.0707e-23 | 3.1329e-11 | 11.4048 |
| 15 | 0.1668 | 0.0438 | 9.0157e-38 | 7.6305e-19 | 13.1476 |
| 16 | 0.1219 | 0.0459 | 1.9162e-36 | 1.1803e-16 | 12.7238 |
| 17 | 0.1047 | 0.0451 | 7.1816e-15 | 3.3135e-08 | 12.1142 |
| 18 | 0.0467 | 0.0404 | 0.0018916 | 0.0037401 | 10.4502 |
| 19 | 0.0729 | 0.0523 | 6.6800e-05 | 0.00084806 | 12.8249 |
| 20 | 0.0457 | 0.0419 | 0.83990 | 0.38759 | 11.0420 |
| 21 | 0.0700 | 0.0448 | 0.00017350 | 0.0010344 | 11.5548 |
| 22 | 0.0985 | 0.0508 | 0.010301 | 0.0031624 | 13.1214 |

**Table S3B.** Comparison of the coefficient of variation of relative read count between regions in 6 CVS controls libraries displaying high and low numbers of reported structural variants in the DGV database. ***hi.dgv****:* average cv.RTC of regions with high GVF [>=95% quantile of the GVF in that chromosome]. ***lo.dgv****:* average cv.RTC of regions with GVF < 95% quantile. wilcox.pval: p value of the Wilcoxon rank sum test comparing cv.RTC of regions with GVF >=95% quantile against cv.RTC of regions with GVF < 95% quantile. ***t.pval****:* two sample t test p value for the same comparison. ***dgv.cutoff****:* 95% quantile of the log2(GVF+1) in that chromosome

| chr | hi.dgv | lo.dgv | wilcox.pval | t.pval | dgv.cutoff |
| --- | --- | --- | --- | --- | --- |
| 1 | 0.0801 | 0.0320 | 5.6593e-41 | 2.1364e-18 | 11.6475 |
| 2 | 0.0478 | 0.0309 | 7.7245e-15 | 2.2019e-11 | 10.8597 |
| 3 | 0.0466 | 0.0298 | 4.2384e-06 | 0.00012693 | 10.7673 |
| 4 | 0.0608 | 0.0299 | 6.4519e-18 | 4.8215e-08 | 11.1087 |
| 5 | 0.0616 | 0.0306 | 7.5945e-12 | 4.3997e-10 | 11.1724 |
| 6 | 0.0508 | 0.0306 | 3.1475e-11 | 1.6434e-06 | 10.9848 |
| 7 | 0.0590 | 0.0320 | 1.9762e-14 | 1.3449e-09 | 11.8331 |
| 8 | 0.0873 | 0.0307 | 1.0992e-22 | 1.9197e-08 | 11.4012 |
| 9 | 0.0882 | 0.0340 | 2.1133e-22 | 5.4755e-10 | 11.9397 |
| 10 | 0.0479 | 0.0321 | 2.9137e-12 | 9.3595e-08 | 11.4779 |
| 11 | 0.0487 | 0.0318 | 1.2073e-10 | 1.0174e-06 | 11.3922 |
| 12 | 0.0535 | 0.0313 | 1.0181e-09 | 6.9874e-06 | 11.0287 |
| 13 | 0.0366 | 0.0300 | 0.030447 | 0.031628 | 10.8639 |
| 14 | 0.0854 | 0.0308 | 1.5332e-20 | 7.4708e-12 | 11.4048 |
| 15 | 0.2385 | 0.0341 | 7.6304e-31 | 1.4838e-15 | 13.1476 |
| 16 | 0.1555 | 0.0354 | 2.7254e-32 | 1.9739e-15 | 12.7238 |
| 17 | 0.1139 | 0.0356 | 1.3089e-16 | 1.2815e-09 | 12.1142 |
| 18 | 0.0395 | 0.0306 | 7.6884e-05 | 0.00035277 | 10.4502 |
| 19 | 0.0803 | 0.0398 | 7.4090e-05 | 8.6660e-05 | 12.8249 |
| 20 | 0.0414 | 0.0324 | 0.0038751 | 0.092227 | 11.0420 |
| 21 | 0.0596 | 0.0338 | 1.0776e-07 | 9.9733e-05 | 11.5548 |
| 22 | 0.0885 | 0.0401 | 0.0018230 | 0.013429 | 13.1214 |
